# Homoleptic complexes of titanium(iv) fused with O^N^O Schiff base derivatives: design, BSA–DNA interaction, molecular docking, DFT and cytotoxicity†

**DOI:** 10.1039/d5ra03821a

**Published:** 2025-07-16

**Authors:** Shivabasayya V. Salimath, Kavita B. Hiremath, Mahabarathi Subramaniyan, Arjita Ghosh, Evangeline Lawrence, Anbalagan Moorthy, Murugesh Shivashankar, Madhvesh Pathak

**Affiliations:** a Department of Chemistry, School of Advanced Sciences, Vellore Institute of Technology (VIT) Vellore Tamil Nadu India madhveshpathak@vit.ac.in; b Department of Integrative Biology, School of Bioscience and Technology (SBST), Vellore Institute of Technology (VIT) Vellore Tamil Nadu India

## Abstract

A set of six Ti(iv) complexes (Ti-1-IS, Ti-2-IN, Ti-3-IO, Ti-4-IF, Ti-5-ICl and Ti-6-IBr) associated with (*Z*)-*N*-((*E*)-2-hydroxybenzylidene) isonicotinohydrazonic acid derivatives were developed while treating titanium(iv) isopropoxide with the appropriate ligands in a stoichiometry of 1 : 2 using anhydrous tetrahydrofuran (THF). Later, the purified products were characterized by employing the spectral techniques FTIR, UV-vis, NMR and HRMS. Then, these newly established complexes were subjected to biomedical applications such as DNA–BSA interaction and *in vitro* cytotoxic investigations. Afterwards, the DNA/BSA binding results of these six complexes revealed that complexes Ti-4-IF and Ti-5-ICl displayed greater binding constant values with DNA and BSA of 2.97 × 10^5^ M^−1^ and 0.065 × 10^4^ M^−1^, respectively. Ethidium bromide (EtBr) was competitively displaced from DNA by the groove-binding mechanism on employing titanium(iv) complexes, and this observation was well supported by viscosity, cyclic voltammetry and *in silico* investigations. Electronic characteristics and molecular representation of Ti(iv) complexes were ascertained using the DFT technique. Subsequently, to explore the anticancer potential of these titanium complexes, an MTT assay was executed against non-cancerous HEK (human embryonic kidney), HeLa (cervical carcinoma) and MCF7 (breast adenocarcinoma) cells, wherein Ti-3-IO (27.17 μM) and Ti-5-ICl (24.25 μM) exhibited low IC_50_ values, to emerge as noteworthy cytotoxic agents against the latter two cancer cell lines. Interestingly, the viability of the HeLa cell line was found to have decreased significantly by the pronounced activity of these complexes. Further, acridine orange–ethidium bromide (AO–EB) staining, cell cycle analysis by propidium iodide (PI) staining and determination of reactive oxygen species (ROS) were also carried out to determine the potency and credibility of titanium(iv) derivatives.

## Abstract 2

A set of six Ti(iv) complexes were examined for *in vitro* DNA and BSA binding investigations and their cytotoxic assays were steered to ascertain the antiproliferative capability of these Ti(iv) derivatives.

## Graphical abstract

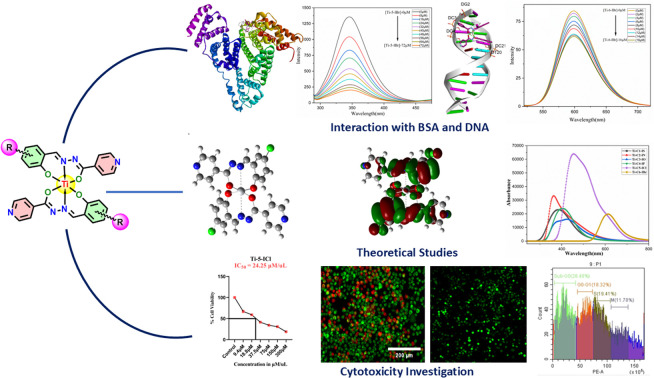

## 1 Introduction

The incidental discovery of *cis*-platin has steered the focus of medicinal chemistry towards the promising field of metal-based therapeutics. In the present scenario, metallodrugs based on platinum are used to treat almost half of all cancer cases. The search for effective non-platinum medications was encouraged by the fact that platinum-based metallodrugs are linked to certain unfavourable side effects, such as lack of selectivity and inherent or acquired resistance toward the drug. Over time, it has been reported that compounds containing titanium, copper, nickel, zinc, cobalt, iron, iridium, ruthenium, rhenium, osmium, gold, *etc.* demonstrate remarkable anticancer properties compared with *cis*-platin 1–5.

Schiff bases (azomethines) are a class of organic compound formed by amino and carbonyl compounds to appear as multidentate ligands that could be used to create extremely significant complexes with distinct metal ions. They use azomethine nitrogen to coordinate with metal ions. The Schiff base reaction in organic synthesis is crucial in creating C–N bonds. They exhibit chelation properties *via* O, N and S donors and the resulting metal complexes emerge with a broad range of biological activity against different types of infections and malignancies.^6,7^

They also possess a variety of clinical, pharmacological and biochemical properties. These compounds exhibit numerous biological characteristics, mostly due to the presence of the imine group. These azomethine compounds are also utilized as polymer stabilizers,^8^ corrosion inhibitors, pigments, dyes, catalysts,^8,9^ antioxidants,^10^ antimicrobials^11^ and anticancer agents.^12–16^ Every metal ion is likely to be chelated by Schiff base complexes due to their multidentate ligand property. These ligands have great potential for an attractive new therapeutic strategy that could help us understand disorders better and aid in their cure. It has been mentioned that the Schiff base complexes of both inner and outer transition metals, which include NO or NOS donor atoms, play a crucial role in biological processes.^17–19^

Arylhydrazones play a pivotal role in the advancement of transition metal coordination chemistry, owing to their tautomeric form that contributes to the coordination mode and denticity variety.^20^ A further point of stabilization towards complexation is provided by the inclusion of distinct donor functional groups in aryl hydrazones at the ortho position concerning the azomethine scaffold (HC=N), which also controls keto–enol tautomerism. As a result, these ligands can form 1 : 1 or 1 : 2 complexes by acting neutrally, and mono- or bi-deprotonated. Although aryl hydrazones produce stable metal complexes mostly in their enol forms, they can also exhibit keto–enol tautomerism in solution.^21^

At the beginning of the previous century, *i.e.* 1912, isoniazid (nicotinic acid hydrazide) was identified and used widely in treating tuberculosis (TB). Isoniazid works by preventing the production of mycolic acid, a crucial component of the TB cell wall.^22–25^ Significant interest has also been observed recently in the chemistry of isoniazid Schiff bases and their metal complexes. As isoniazid has an amino group, it can react with other carbonyl substances (ketones and aldehydes) to generate Schiff bases or hydrazones possessing distinctive imine groups supported by the carbonyl group. In certain instances, tautomerism can occur between the amino and carbonyl groups in the isoniazid Schiff base moiety. The inclusion of OH or NH groups in a suitable orientation has greater potential to enhance intramolecular and intermolecular interactions *via* the HC=N– group. This suggests that, because of its enhanced interactions with the binding sites, the isoniazid Schiff base might exhibit substantially stronger bioactivity. Transition metal complexes of isoniazid Schiff bases have been studied and apprehended in cytotoxic studies.^26^

Titanium is a desirable metal because of its relative abundance in the earth's crust and its low toxicity. For example, food packaging and wraps often include titanium dioxide, which is proven to have no negative effects. Nevertheless, titanium is prone to rapid and spontaneous hydrolysis, which might result in the uncontrollable production of titanium(iv) oxide because of its strong polarizing ability as well as minimal electronegativity.^27^

The strength of the complexing ligand while modifying pH and careful selection of titanium precursors, such as titanium(iv) chloride or titanium(iv) alkoxides, also contribute to the stability of Ti^4+^ in solution form and ease of the synthetic process. As titanium(iv) alkoxides are less volatile than tetrahalides and include hydrophobic alkoxy groups, they are frequently chosen as potential precursors for safe experimental conditions.^28,29^

Due to their rapid hydrolysis and poor efficacy, titanocene dichloride and budotitane could not qualify for phase II clinical trials in the cancer field despite showing good efficacy in *in vivo* studies and less toxicity than *cis*-platin. In order to overcome these challenges, many researchers across the globe have developed a few cytotoxic polydentate complexes based on titanium that have demonstrated both *in vitro* and *in vivo* cytotoxicity against diverse cancer cells along with a comparatively high degree of hydrolytic stability. Hexadentate Ti(iv) derivatives have also been exploited to stabilize the situation, resulting in extremely cytotoxic complexes that remained stable even in water. These observations suggested that labile ligands are not necessary for antiproliferative activities, according to recent findings on inert Ti(iv) complexes with regard to anticancer capabilities.^30–35^

**Fig. 1:**
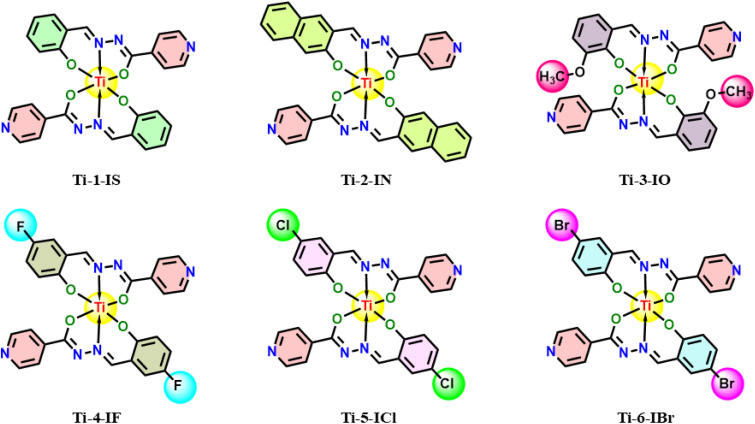
Structures of newly synthesized Ti(iv) complexes.

Thus, in line with the abovementioned reports, the synthesis of a series of half a dozen new Ti(iv) complexes (Fig. 1) incorporating ONO ligands derived from isoniazid-salicylaldehyde Schiff-bases was carried out while performing reactions of isoniazid with salicylaldehyde derivatives and Ti(O^i^Pr)_4_. Complexes Ti-1-IS to Ti-6-IBr appeared as a new class of homoleptic mononuclear derivatives of titanium(iv) and were well characterized using relevant spectroscopic techniques. Subsequently, binding interactions were carried out with BSA and CT-DNA, and molecular docking studies were also performed to support the binding studies and DFT computations. Eventually, cytotoxic investigations were also conducted against HeLa, MCF7 and HEK-293 cell lines using an MTT assay. HeLa cells were subjected to cell cycle analysis while performing PI staining for apoptosis detection. Later, ROS quantification was carried out with a DCFH-DA staining assay.

## 2 Experimental section

### 2.1 Physical measurements

A Jasco V-670 UV-visible spectrometer with range 600–200 nm, a Thermo Fisher Scientific Nicolet iS50 spectrophotometer, an ATR mode infrared spectrometer (4000–400 cm^−1^) and a Hitachi F-7000 200–800 nm fluorescence spectrophotometer were used to record the absorption, transmittance and luminescence spectra of the compounds at 293 K. Regarding ligands and complexes, ^1^H and ^13^C NMR spectra were recorded on a Bruker Avance 400 MHz spectrometer using Si(CH_3_)_4_ as internal standard and DMSO-d_6_ as solvent. Electrospray ionization mass spectrometry (ESI-MS) was run on a Waters Xevo G2-XS mass spectrometer. Cyclic voltammograms were obtained with a CH Instruments machine, and an Elico CM 180 conductivity meter was used for conductivity analysis. A Bio-Rad xMARK™ Microplate Spectrophotometer was used to record the intensity of the plates, an Olympus Confocal Laser Scanning Microscope—Fluoview FV 3000—was used to capture the images, and a FACS scanner and the Becton Dickinson cell search program were incorporated to determine cell cycle analysis.

### 2.2 Materials and methods

For synthesizing the complexes, the aldehydes, salicylaldehyde, 5-chloro-salicylaldehyde, 5-fluoro-salicylaldehyde, 3-methoxysalicylaldehyde and isoniazid were procured from AVRA Synthesis and SRL Chemicals, and 2-hydroxy-1-naphthaldehyde, 5-bromo-salicylaldehyde and titanium isopropoxide Ti(O^i^Pr)_4_ were purchased from Sigma-Aldrich and were used as obtained. Calf thymus DNA (CT-DNA) and 2,2-diphenyl-1-picrylhydrazyl (DPPH) were obtained from Sigma-Aldrich, and BSA was purchased from Himedia Chemicals. Analytical and spectroscopic grades of chemicals and solvents were purchased from SRL, Avra and SD Fine-Chem Ltd and underwent standard purification involving drying and distillation methods before use. The cancer cell lines, cervical cancer (HeLa) passage number 30, human breast cancer cell line (MCF-7) passage number 35 and human kidney normal cell line (HEK-293) passage number 50 were procured from the National Centre for Cell Science (NCCS), Pune, India.

### 2.3 Synthesis of ligands

#### 2.3.1 (*Z*)-*N*-((*E*)-2-Hydroxybenzylidene)isonicotinohydrazonic acid (IS)

To a methanolic solution of salicylaldehyde (0.500 g, 4.1 mmol), isoniazid (0.560 g, 4.1 mmol) was added dropwise at a 1 : 1 molar ratio by stirring at room temperature. The reaction mixture turned into pale-yellow color and was further stirred at 40 °C for 8 h. The reaction was monitored by TLC for completion. The reaction mixture was filtered to yield a yellow-coloured product and repeatedly washed with cold methanol and further dried *in vacuo*, which afforded the product ligand (IS) in the form of a pale-yellow powder,^36^ which was characterized by FTIR, NMR (^1^H and ^13^C) and electrospray ionization mass spectrometry (ESI-MS), as displayed in [ESI†].

Yield: 95%; pale yellow solid; m.p. 243–244 °C. ^1^H NMR (DMSO-d6, 400 MHz, *δ*, ppm): 6.922–6.972 (q, 2H, *j* = 6.0 Hz, CHAr), 7.330–7.335 (t, 1H, *j* = 1 Hz, CHAr), 7.602–7.627 (dd 1H, *j* = 4.4 Hz, CHAr), 7.855–7.870 (d, 2H, *j* = 3 Hz, CHAr), 8.699 (s, 1H, N=CH), 8.807–8.822 (d, 1H, *J* = 3 Hz, CHAr), 11.101 (s, 1H, C–OH), 12.310 (s, 1H, C–NH) (ESI Fig. S1†). ^13^C NMR (DMSO-d6, 100 MHz, *δ*, ppm): 116.91, 119.15, 119.90, 121.98, 123.18, 129.68, 131.79, 132.21, 140.44, 143.59, 150.14, 150.86, 157.94 (C=N), 161.81 (–NHC=O) (ESI Fig. S2†). FTIR (ATR, cm^−1^): 3181.97 w (NH), 3002.63 w (OH), 2834.83 w (CH), 1678 s (C=N amide), 1566.87, 1488.76, 1272.78, 1157.08, 849.48, 764.63, 638.64 (br, broad; s, strong; m, medium; w, weak) (ESI Fig. S3†). Electrospray ionization (ESI)-MS: 242.0930 (M+) (ESI Fig. S4†).

#### 2.3.2 (*Z*)-*N*-(*E*)-(2-Hydroxynaphthalen) isonicotinohydrazonic acid (IN)

The ligand (IN) was synthesized by adding to a methanolic solution of 2-hydroxy-1-naphthaldehyde (0.500 g, 2.9 mmol), isoniazid (0.398 g, 2.9 mmol), which was added dropwise in 1 : 1 molar ratio by stirring at room temperature. The reaction mixture was further stirred at 40 °C for 8 h to yield a lime yellow product. The reaction was monitored by TLC for completion. The reaction mixture was filtered to yield a yellow-colored product and repeatedly washed with cold methanol and further dried *in vacuo* to obtain the product in the form of a yellow powder.^37^

Yield: 89%; yellow solid; m.p. 248–250 °C. ^1^H NMR (DMSO-d6, 400 MHz, *δ*, ppm): 7.251–7.273 (d, 1H, *j* = 4.4 Hz, CHAr), 7.411–7.448 (t, 1H, *j* = 7.4 Hz, CHAr), 7.615–7.653 (t 1H, *j* = 7.6 Hz, CHAr), 7.899–7.974 (q, 4H, *j* = 14.2 Hz, CHAr), 8.318–8.339 (d, 1H, *J* = 4.2 Hz, CHAr), 8.848–8.61 (d, 3H, *J* = 2.6 Hz, CHAr), 9.497 (s, 1H, N=CH), 12.420 (s, 1H, C–OH), 12.556 (s, 1H, C–NH) (ESI Fig. S5†). ^13^C NMR (DMSO-d6, 100 MHz, *δ*, ppm): 108.99, 119.31, 121.43, 121.92, 122.63, 124.10, 128.33, 128.39, 129.47, 132.07, 133.62, 140.28, 148.45, 150.27, 150.96, 158.68 (C=N), 161 (–NHC=O) (ESI Fig. S6†). FTIR (ATR, cm^−1^): 3221.50 w (OH), 3037.33, 1673.90 s (C=N amide), 1548.55, 1468.52, 1274.71, 951.69, 740.56, 684.60 (br, broad; s, strong; m, medium; w, weak) (ESI Fig. S7†). Electrospray ionization (ESI)-MS: 292.1089 (M+) (ESI Fig. S8†).

#### 2.3.3 (*Z*)-*N*-((*E*)-3-Methoxy-2-hydroxybenzylidene)isonicotinohydrazonic acid (IO)

The ligand (IO) was synthesized by treating a methanolic solution of 3-methoxysalicylaldehyde (0.500 g, 3.24 mmol), to which isoniazid (0.450 g, 3.24 mmol) was added dropwise in 1 : 1 molar ratio by stirring at room temperature. The reaction mixture was stirred at 40 °C for 8 h. The reaction was monitored by TLC for completion. The reaction mixture was filtered and repeatedly washed with cold methanol and further dried *in vacuo*, which yielded the product in the form of a white powder.^38^

Yield: 82%; white solid; m.p. 234–236 °C. ^1^H NMR (DMSO-d6, 400 MHz, *δ*, ppm): 3.835 (s, 1H, O–CH_3_), 6.865–6.905 (t, 1H, *j* = 8 Hz, CHAr), 7.053–7.071 (d, 1H, *j* = 3.6 Hz, CHAr), 7.210–7.229 (t 1H, *j* = 3.8 Hz, CHAr), 7.856–7.871 (d, 2H, *j* = 3 Hz, CHAr), 8.718 (s, 1H, N=CH), 8.806–8.821 (d, 2H, *J* = 3 Hz, CHAr), 10.723 (s, 1H, C–OH), 12.283 (s, 1H, C–NH) (ESI Fig. S9†). ^13^C NMR (DMSO-d6, 100 MHz, *δ*, ppm): 114.42, 118.82, 119.44, 119.61, 120.88, 121.98, 123.22, 140.48, 143.43, 146.56, 147.63, 148.45, 149.29, 150.12, 150.85 (C=N), 161.78 (–NHC=O) (ESI Fig. S10†). FTIR (ATR, cm^−1^): 3141.98 w (NH), 2957.30 w (OH), 2804.95 w (CH), 1676 s (C=N amide), 1551.45, 1475.27, 1265.07, 929.59, 796.68, 677.89 (br, broad; s, strong; m, medium; w, weak) (ESI Fig. S11†). Electrospray ionization (ESI)-MS: 272.1038 (M+) (ESI Fig. S12†).

#### 2.3.4 (*Z*)-*N*-((*E*)-5-Fluoro-2-hydroxybenzylidene)isonicotinohydrazonic acid (IF)

To a methanolic solution of 5-chloro-salicylaldehyde (0.500 g, 3.2 mmol), isoniazid (0.438 g, 3.2 mmol) was added dropwise in 1 : 1 molar ratio by stirring at room temperature and was further stirred at 40 °C for 8 h. The reaction was monitored by TLC for completion. The reaction mixture was filtered and repeatedly washed with cold methanol and further dried *in vacuo*, which afforded the product ligand (IF) in the form of a lime yellow powder.^39^

Yield: 91%; lime yellow solid; m.p. 252–254 °C. ^1^H NMR (DMSO-d6, 400 MHz, *δ*, ppm): 6.943–6.977 (q, 1H, *j* = 6.8 Hz, CHAr), 7.150–7.179 (q, 1H, *j* = 5.8 Hz, CHAr), 7.459–7.490 (dd 1H, *j* = 6.2 Hz, CHAr), 7.854–7.869 (d, 2H, *j* = 3 Hz, CHAr), 8.690 (s, 1H, N=CH), 8.806–8.821 (d, 2H, *J* = 3 Hz, CHAr), 10.860 (s, 1H, C–OH), 12.348 (s, 1H, C–NH) (ESI Fig. S13†). ^13^C NMR (DMSO-d6, 100 MHz, *δ*, ppm): 113.84, 114.07, 118.10, 118.17, 118.73, 118.97, 120.23, 120.31, 121.99, 123.30, 140.41, 147.39, 150.10, 150.85, 154.11, 154.68, 157.01 (C=N), 161.93 (–NHC=O) (ESI Fig. S14†). FTIR (ATR, cm^−1^): 3157.86 w (NH), 2930.36 w (OH), 2802.86 w (CH), 1681.62 s (C=N amide), 1554.32, 1483.95, 1284.35, 1140.68, 779.90, 679.78 (br, broad; s, strong; m, medium; w, weak) (ESI Fig. S15†). Electrospray ionization (ESI)-MS: 260.0839 (M+) (ESI Fig. S16†).

#### 2.3.5 (*Z*)-*N*-((*E*)-5-Chloro-2-hydroxybenzylidene)isonicotinohydrazonic acid (ICl)

The reactants 5-fluoro-salicylaldehyde (0.500 g, 3.56 mmol) and isoniazid (0.488 g, 3.56 mmol) were added in a 1 : 1 molar ratio in methanol solution and stirred at 40 °C for 8 h. The reaction was monitored by TLC for the completion. The reaction mixture was filtered and repeatedly washed with cold methanol and further dried *in vacuo*, which afforded the product ligand (ICl) in the form of a white powder.^39^

Yield: 85%; white solid; m.p. 268–269 °C. ^1^H NMR (DMSO-d_6_, 400 MHz, *δ*, ppm): 6.965–6.987 (d, 1H, *j* = 4.4 Hz, CHAr), 7.329–7.358 (dd, 1H, *j* = 5.8 Hz, CHAr), 7.711–7.718 (d 1H, *j* = 1.4 Hz, CHAr), 7.853–7.868 (dd, 2H, *j* = 3 Hz, CHAr), 8.674 (s, 1H, N=CH), 8.805–8.820 (d, 2H, *J* = 3 Hz, CHAr), 11.136 (s, 1H, C–OH), 12.374 (s, 1H, C–NH) (ESI Fig. S17†). ^13^C NMR (DMSO-d6, 100 MHz, *δ*, ppm): 118.73, 121.16, 121.99, 123.55, 127.74, 131.15, 140.37, 147.01, 150.07, 150.85, 156.53 (C=N), 161.97 (–NHC=O) (ESI Fig. S18†). FTIR (ATR, cm^−1^): 3197.81 w (NH), 3002.62 w (OH), 2841.59 w (CH), 1687.40 s (C=N amide), 1563.98, 1462.72, 1244.82, 1064.51, 738.60, 685.57 (br, broad; s, strong; m, medium; w, weak) (ESI Fig. S19†). Electrospray ionization (ESI)-MS: 276.0544 (M+) (ESI Fig. S20†).

#### 2.3.6 (*Z*)-*N*-((*E*)-5-Bromo-2-hydroxybenzylidene)isonicotinohydrazonic acid (IBr)

The reactants 5-bromo-salicylaldehyde (0.500 g, 2.48 mmol) and isoniazid (0.334 g, 2.48 mmol) were added in a 1 : 1 molar ratio in methanol solvent by stirring at room temperature, at 40 °C for 8 h. The reaction was monitored by TLC for completion. The reaction mixture was filtered and repeatedly washed with cold methanol and further dried *in vacuo*, which afforded the product (IBr) in the form of a white powder.^40^

Yield: 79%; white solid; m.p. 255–258 °C. ^1^H NMR (DMSO-d6, 400 MHz, *δ*, ppm): 6.917–6.938 (d, 1H, *j* = 4.2 Hz, CHAr), 7.442–7.470 (dd, 1H, *j* = 5.6 Hz, CHAr), 7.841–7.869 (m 3H, *j* = 5.6 Hz, CHAr), 8.805 (s, 1H, N=CH), 8.817–8.820 (d, 2H, *J* = 0.6 Hz, CHAr), 11.150 (s, 1H, C–OH), 12.375 (s, 1H, C–NH) (Fig. S21†). ^13^C NMR (DMSO-d6, 100 MHz, *δ*, ppm): 111.02, 119.17, 121.99, 123.27, 130.59, 134.37, 140.36, 141.28, 146.82, 150.06, 150.84, 156.93 (C=N), 161.97 (–NHC=O) (Fig. S22†). FTIR (ATR, cm^−1^): 3178.89 w (NH), 3007.44 w (OH), 2803.99 w (CH), 1672.94 s (C=N amide), 1553.38, 1472.38, 1265.07, 1188.59, 793.56, 673.92 (br, broad; s, strong; m, medium; w, weak) (Fig. S23†). Electrospray ionization (ESI)-MS: 320.0037 (M+) (ESI Fig. S24†).

### 2.4 Synthesis of complexes

#### 2.4.1 Synthesis of complex Ti-1-IS

The Ti(iv) complex Ti-1-IS was synthesized by treating titanium isopropoxide Ti (O^i^Pr)_4_ (0.5 mmol, 135 milligrams) with 2 equivalents of synthesized ligand (L1) (1.0 mmol, 500 milligrams) in dry THF. The reaction mixture was refluxed for 8 h and allowed to cool at room temperature and repeatedly washed with dry hexane and dried under vacuum to obtain a dark orange complex. The obtained complex was characterized by FTIR, NMR (^1^H and ^13^C), HPLC and HR-MS [for data see ESI†].

Yield: 90%; orange solid; m.p. > 300 °C. ^1^H NMR (DMSO-d6, 400 MHz, *δ*, ppm): 2.256 (S 3H, CH3), 6.715–6.736 (d, 1H, *j* = 4.2 Hz, CHAr), 6.856–6.926 (m, 3H, *j* = 14 Hz, CHAr), 7.035–7.073 (t, 1H, *j* = 7.6 Hz, CHAr), 7.248–7.284 (t, 1H, *j* = 7.2 Hz, CHAr), 7.519–7.560 (t, 2H, *j* = 8.2 Hz, CHAr), 7.802–7.824 (d, 1H, *J* = 4.4 Hz, CHAr), 8.238–8.255 (t, 1H, *J* = 3.4 Hz, CHAr), 8.427–8.443 (d, 2H, *J* = 1.2 Hz, CHAr), 8.701 (s, 1H, CHAr), 8.874–8.890 (d, 2H, *J* = 3.2 Hz, CHAr), 9.085–9.101 (d, 2H, *J* = 3.2 Hz, CHAr), 9.150 (s, 1H, N=CH) (ESI Fig. S25†). ^13^C NMR (DMSO-d6, 100 MHz, *δ*, ppm): 111.07, 113.93, 115.87, 116.79, 116.83, 118.75, 119.65, 119.68, 120.96, 122.52, 125.21, 125.39, 126.56, 128.47, 129.75, 131.82, 132.29, 135.19, 137.70, 142.87, 143.31, 143.87, 145.44, 147.83, 150.95, 158.14, 158.31, 158.69, 159.08, 159.26, 159.47, 162.14, 162.38, 164.42 (C=N) (ESI Fig. S26†). FTIR (ATR, cm^−1^): 3023.86 w (CH), 1588.09 s (C=N amide), 1544.73, 817.67, 697.15, 584.32 (Ti–N), 496.58, 450.29 (Ti–O) (br, broad; s, strong; m, medium; w, weak) (ESI Fig. S27†). Electrospray ionization (ESI)-MS: 527.2437 (M + H) (ESI Fig. S28†). Purity was analyzed by HPLC (ESI Fig. S29†).

#### 2.4.2 Synthesis of complex Ti-2-IN

The Ti(iv) complex Ti-2-IN was synthesized by treating titanium isopropoxide Ti(O^i^Pr)_4_ (0.5 mmol, 135 milligrams) with 2 equivalents of synthesized ligand (L2) (1.0 mmol, 595 mg) in dry THF. The reaction mixture was refluxed for 8 h and allowed to cool at room temperature, repeatedly washed with dry hexane and dried under vacuum to obtain a brownish-black complex. The obtained complex was characterized by FTIR, NMR (^1^H and ^13^C), HPLC and HR-MS.

Yield: 78%; brownish-black solid; m.p. > 300 °C. ^1^H NMR (DMSO-d6, 400 MHz, *δ*, ppm): 7.567–7.587 (d, 1H, *j* = 4 Hz, CHAr), 7.759–7.779 (d, 2H, *j* = 4 Hz, CHAr), 7.859–7.946 (t, 2H, *j* = 7.6 Hz, CHAr), 8.016–8.050 (t, 1H, *j* = 6.8 Hz, CHAr), 8.107–8.144 (t, 2H, *j* = 7.4 Hz, CHAr), 8.371–8.41 (d, 4H, *J* = 2.5 Hz, CHAr), 8.618–8.643 (t, 1H, *J* = 5 Hz, CHAr), 8.797–8.866 (m, 2H, *J* = 7 Hz, CHAr), 9.083–9.100 (m, 2H, *J* = 3.4 Hz, CHAr), 9.404 (s, 1H, CHAr), 9.675–9.688 (d, 2H, *J* = 2.6 Hz, CHAr), 10.101 (s, 1H, N=CH), 10.390 (s, 1H, CHAr) (ESI Fig. S30†). ^13^C NMR (DMSO-d6, 100 MHz, *δ*, ppm): 108.52, 110.92, 113.77, 116.62, 119.05, 119.48, 120.77, 123.79, 125.00, 125.40, 127.98, 128.21, 129.13, 129.31, 129.44, 132.09, 133.61, 142.69, 143.41, 148.01, 149.74, 158.25, 158.64, 159.03, 159.42 (ESI Fig. S31†). FTIR (ATR, cm^−1^): 3022.68 w (CH), 1590.98 s (C=N amide), 1540.85, 822.68, 692.31, 580.46 (Ti–N), 496.58, 420.40 (Ti–O) (br, broad; s, strong; m, medium; w, weak) (ESI Fig. S32†). Electrospray ionization (ESI)-MS: 627.1335 (M+) (ESI Fig. S33†). Purity was analyzed by HPLC (ESI Fig. S34†).

#### 2.4.3 Synthesis of complex Ti-3-IO

The Ti(iv) complex Ti-3-IO was synthesized by treating titanium isopropoxide Ti(O^i^Pr)_4_ (0.5 mmol, 135 milligrams) with 2 equivalents of synthesized ligand (L3) (1.0 mmol, 557 milligrams) in dry THF. The reaction mixture was refluxed for 8 h and allowed to cool at room temperature and repeatedly washed with dry hexane and dried under vacuum to obtain an orange-coloured complex. The obtained complex was characterized by FTIR, NMR (^1^H and ^13^C), HPLC and HR-MS.

Yield: 83%; orange solid; m.p. > 300 °C. ^1^H NMR (DMSO-d6, 400 MHz, *δ*, ppm): 3.701 (s, 3H, O–CH_3_), 3.798 (s, 3H, O–CH_3_), 6.813–6.854 (t, 1H, *j* = 8.2 Hz, CHAr), 6.993–7.038 (d, 2H, *j* = 9 Hz, CHAr), 7.185–7.235 (q, 2H, *j* = 10 Hz, CHAr), 7.389–7.409 (d, 1H, *j* = 4 Hz, CHAr), 8.217–8.233 (d, 2H, *j* = 3.2 Hz, CHAr), 8.420–8.437 (d, 2H, *J* = 3.4 Hz, CHAr), 8.743 (s, 1H, CHAr), 8.877–8.894 (d, 2H, *J* = 3.4 Hz, CHAr), 9.095–9.111 (d, 2H, *J* = 3.2 Hz, CHAr), 9.168 (s, 1H, N=CH) (ESI Fig. S35†). ^13^C NMR (DMSO-d6, 100 MHz, *δ*, ppm): 55.93, 55.96, 111.13, 114.00, 114.41, 116.86, 119.72, 120.70, 121.32, 122.61, 125.19, 125.32, 126.33, 126.59, 128.53, 142.99, 143.54, 144.11, 145.11, 146.12, 146.67, 147.68, 147.77, 148.41, 150.41, 152.50, 158.34, 158.72, 159.11, 159.36, 159.49, 162.47, 164.43 (C=N) (ESI Fig. S36†). FTIR (ATR, cm^−1^): 3033.58 w (CH), 1590.57 s (C=N amide), 1554.35, 840.08, 697.14, 593.00 (Ti–N), 498.50, 427.15 (Ti–O) (br, broad; s, strong; m, medium; w, weak) (ESI Fig. S37†). Electrospray ionization (ESI)-MS: 587.1223 (M+) (ESI Fig. S38†). Purity was analyzed by HPLC (ESI Fig. S39†).

#### 2.4.4 Synthesis of complex Ti-4-IF

The Ti(iv) complex Ti-4-IF was synthesized by treating titanium isopropoxide Ti(O^i^Pr)_4_ (0.5 mmol, 135 milligrams) with 2 equivalents of synthesized ligand (L4) (1.0 mmol, 534 mg) in dry THF. The reaction mixture was refluxed for 8 h and allowed to cool at room temperature and repeatedly washed with dry hexane and dried under vacuum to obtain a red complex. The obtained complex was characterized by FTIR, NMR (^1^H, ^13^C and ^19^F) and HPLC and HR-MS.

Yield: 88%; red solid; m.p. > 300 °C. ^1^H NMR (DMSO-d6, 400 MHz, *δ*, ppm): 6.917–6.939 (t, 2H, *j* = 4.4 Hz, CHAr), 7.082–7.090 (d, 2H, *j* = 1.6 Hz, CHAr), 7.408–7.439 (dd, 2H, *j* = 6.2 Hz, CHAr), 8.270–8.286 (d, 1H, *j* = 3.2 Hz, CHAr), 8.422–8.438 (d, 3H, *j* = 3.2 Hz, CHAr), 8.707 (s, 2H, N=CH), 9.098–9.114 (d, 4H, *J* = 3.2 Hz, CHAr) (ESI Fig. S40†). ^13^C NMR (DMSO-d6, 100 MHz, *δ*, ppm): 111.14, 111.46, 113.67, 113.91, 114.00, 116.86, 117.74, 117.82, 117.99, 118.07, 118.35, 118.58, 118.86, 119.09, 119.72, 119.88, 119.96, 120.91, 120.99, 125.33, 126.65, 128.53, 142.77, 142.96, 143.54, 144.14, 145.45, 147.58, 148.64, 150.94, 153.45, 154.25, 154.67, 157.00, 158.35, 158.73, 159.12, 159.29, 159.50, 165.21, 165.83 (C=N) (ESI Fig. S41†). ^19^F NMR (DMSO-d_6_, 100 MHz, *δ*, ppm): −75.86 (ESI Fig. S42†). FTIR (ATR, cm^−1^): 3043.11 w (CH), 1593.87 s (C=N amide), 1552.46, 838.88, 697.14, 570.82 (Ti–N), 498.50, 440.65 (Ti–O) (br, broad; s, strong; m, medium; w, weak) (ESI Fig. S43†). Electrospray ionization (ESI)-MS: 563.3981 (M+) (ESI Fig. S44†). Purity was analyzed by HPLC (ESI Fig. S45†).

#### 2.4.5 Synthesis of complex Ti-5-ICl

The Ti(iv) complex Ti-5-ICl was synthesized by treating titanium isopropoxide Ti(O^i^Pr)_4_ (0.5 mmol, 135 mg) with 2 equivalents of synthesized ligand (L5) (1.0 mmol, 564 mg) in dry THF. The reaction mixture was refluxed for 8 h and allowed to cool at room temperature and repeatedly washed with dry hexane and dried under vacuum to obtain a brown complex. The obtained complex was characterized by FTIR, NMR (^1^H and ^13^C), HPLC and HR-MS.

Yield: 76%; brown solid; m.p. > 300 °C. ^1^H NMR (DMSO-d6, 400 MHz, *δ*, ppm): 6.931–6.953 (d, 1H, *j* = 4.4 Hz, CHAr), 7.264–7.302 (m, 2H, *j* = 7.6 Hz, CHAr), 7.682–7.688 (d, 2H, *j* = 1.2 Hz, CHAr), 8.432–8.449 (d, 5H, *j* = 3.4 Hz, CHAr), 8.695 (s, 2H, N=CH), 9.102–9.119 (d, 4H, *J* = 3.4 Hz, CHAr) (ESI Fig. S46†). ^13^C NMR (DMSO-d6, 100 MHz, *δ*, ppm): 115.86, 118.72, 121.58, 123.34, 124.44, 125.54, 126.40, 128.37, 130.12, 131.39, 132.41, 133.28, 136.46, 148.92, 152.38, 153.13, 160.65, 161.42, 163.08, 163.46, 163.85, 164.23 (C=N) (ESI Fig. S47†), FTIR (ATR, cm^−1^): 3033.51 w (CH), 1588.34 s (C=N amide), 1537.23, 823.21, 684.55, 563.11 (Ti–N), 494.65, 458.01 (Ti–O) (br, broad; s, strong; m, medium; w, weak) (ESI Fig. S48†). Electrospray ionization (ESI)-MS: 594.5966 (M+) (ESI Fig. S49†). Purity was analyzed by HPLC (ESI Fig. S50†).

#### 2.4.6 Synthesis of complex Ti-6-IBr

The Ti(iv) complex Ti-6-IBr was synthesized by treating titanium isopropoxide Ti(O^i^Pr)_4_ (0.5 mmol, 135 mg) with 2 equivalents of synthesized ligand (L6) (1.0 mmol, 648 mg) in dry THF. The reaction mixture was refluxed for 8 h and allowed to cool at room temperature and repeatedly washed with dry hexane and dried under vacuum to obtain a reddish-brown complex. The obtained complex was characterized by FTIR, NMR (^1^H and ^13^C) HPLC and HR-MS.

Yield: 92%; reddish brown solid; m.p. > 300 °C. ^1^H NMR (DMSO-d6, 400 MHz, *δ*, ppm): 6.881–6.904 (d, 2H, *j* = 4.6 Hz, CHAr), 7.383–7.411 (m, 2H, *j* = 5.6 Hz, CHAr), 7.813–7.819 (d, 2H, *j* = 1.2 Hz, CHAr), 8.426–8.441 (d, 4H, *j* = 3 Hz, CHAr), 8.687 (s, 2H, N=CH), 9.100–9.116 (d, 4H, *J* = 3.2 Hz, CHAr) (ESI Fig. S51†). ^13^C NMR (DMSO-d6, 100 MHz, *δ*, ppm): 110.91, 111.10, 113.96, 116.81, 119.04, 119.67, 121.41, 125.39, 126.64, 130.56, 134.53, 142.51, 142.97, 144.03, 147.68, 148.13, 156.33, 157.09, 158.32, 158.70, 159.09, 159.47, 159.50, 165.85 (C=N) (ESI Fig. S52†). FTIR (ATR, cm^−1^): 3022.73 w (CH), 1589.57 s (C=N amide), 1536.98, 822.49, 653.75, 562.48 (Ti–N), 472.47, 459.55 (Ti–O) (br, broad; s, strong; m, medium; w, weak) (ESI Fig. S53†). Electrospray ionization (ESI)-MS: 681.4745 (M+) (ESI Fig. S54†). Purity was analyzed by HPLC (ESI Fig. S55†).

### 2.5 DNA binding studies

The UV-vis titration technique was employed to conduct DNA binding investigations of the newly synthesized Ti(iv)complexes. The binding interactions between Ti(iv) complexes and CT-DNA 5 mM (5 × 10^−5^ M) produced in PBS buffer were assessed in a 5 mM phosphate buffer (PBS) with a pH of 7.4. The ratio of UV absorbance of CT-DNA at each position, 260 and 280 nm, was confirmed. The concentration of each nucleotide was calculated using the molar absorption coefficient (6600 m^−1^) of CT-DNA at 260 nm. Additional dilutions were made in 5 mM PBS buffer solution, and stock solutions of metal complexes were prepared in DMSO. DNA binding experiments were performed with a fixed concentration of the complex (20 μM) and increasing concentrations of CT-DNA (0–80 μM) in sample solutions to detect the CT-DNA absorbance peak.^41,42^

Using fluorescence spectroscopy, the competitive binding relationship between metal complexes and CT-DNA bound to ethidium bromide was investigated. EtBr and CT-DNA solutions at a 5 mM concentration were made in PBS buffer (pH 7.4) for the fluorescence studies. After that, the reference solutions were allowed to settle at room temperature for an hour and the CT-DNA reference solution (200 μM) was coupled to ethidium bromide (50 μM) and allowed to rest for the specified period. The excitation and emission wavelengths were set at 460 nm and incremental addition of Ti(iv) complex concentrations ranging from 0 to 80 μM were used to titrate constant concentrations of EtBr-CT-DNA solution.^43,44^

### 2.6 Viscosity measurements

Using an Ostwald viscometer kept at 298 K, viscosity measurements were utilized to examine the type of DNA binding at constant CT-DNA concentrations (5 × 10^−5^ mol L^−1^) with and without different concentrations of the Ti(iv) complexes (0–9 × 10^−5^ mol L^−1^). The viscometer measurements and the flow time of each sample were carried out in triplicate. The measurements were averaged to determine the viscosity of the samples by utilizing the equation (*η*/*η*^0^)^1/3^*versus* [the compound]/[DNA] = 0.0–1.8, where *η*^0^ and *η* stand for the viscosities of the DNA solution by itself and with the complex present, respectively.^45,46^

### 2.7 Cyclic voltametric studies

Cyclic voltammetry (CV) trials were conducted in a DMSO medium with 0.1 M tetrabutylammonium hexafluorophosphate as the supporting electrolyte at room temperature. Platinum wire served as the counter electrode, while glassy carbon served as the working electrode. The Ag/AgCl reference electrode (double junction, 3 M KCl within a Luggin capillary) was employed for nonaqueous systems. After dissolving the weighed amount of Ti(iv) complexes in the electrolyte to create solutions of 1 mM (1 × 10^−3^ M) concentration, their cyclic voltammograms were then recorded. CT-DNA was dissolved in 0.1 M PBS buffer with a pH adjustment of 7.4 to produce a 5 mM DNA solution. Additionally, throughout the potential range of −1.6 to 1.6 V *vs.* Ag/AgCl, the complexes were titrated discretely with incremental additions of CT-DNA (0–50 μM) at a scan rate of 50 mV s^−1^. OriginPro software was used to plot the curves for current potential values in the CV studies.^47,48^

### 2.8 Gel electrophoresis

The interaction and effectiveness of the Ti(iv) complex on CT-DNA were investigated using the agarose gel electrophoresis technique. This was accomplished by incubating the reaction mixtures separately, each of which contained 20 μL of CT-DNA and 20 μL of titanium complexes in PBS buffer solution, at 310 K for one hour. The tracking bleomycin dye (1 μL) and all of the prepared solutions (5 μL) were then combined and applied to the wells of a 1% agarose gel containing 1 mM ethidium bromide that was prepared well in advance. Then the reaction mixture of complexes and CT-DNA was loaded in the wells separately using 0.25% bleomycin dye. The electrophoresis mobility experiment was then carried out in an EDTA-PBS buffer for 60 minutes at a constant electrical voltage of 100 V until the dye reached 50% of the gel distribution area. In order to evaluate the degree of DNA binding capacity of the Ti(iv) complexes, the resultant bands were finally captured on a camera using the AXYGEN digital camera Gel Documentation System under UV light.^49–51^

### 2.9 BSA binding studies

BSA stock solution (5 mM) was prepared in 5 mM phosphate buffer (pH 7.4) and then kept at 2–6 °C. The binding mechanism of the Ti(iv) complexes with BSA was established using fluorescence titration at 200–500 nm. Metal complexes with BSA were quenched by tracking changes in the fluorescence intensity at a certain excitation wavelength of 285 nm and emission value at 345 nm, respectively.^52^ Identical excitation and emission scan rates and slit widths were employed in all investigations. In order to titrate against different metal complex concentrations ranging from 0 to 80 μM, a constant protein concentration of BSA was used. Similarly, the synchronous fluorescence spectra were also acquired by titrating different metal complex concentrations ranging from 0 to 80 μM while maintaining a constant BSA at the specified excitation wavelengths, *λ* = 15 nm and 60 nm.^53,54^

Fluorescence emission spectroscopy was used for site marker investigation, using an excitation wavelength of 280 nm. As they bind to the two most prevalent drug-binding sites, Sudlow sites I and II, two distinct site marker medications, warfarin (WAR) and ibuprofen (IBU), were utilized as particular site markers (probes) in this experiment. Separately, 10 mg of the site marker probes (WAR) and (IBU) were dissolved in PBS buffer. Subsequently, a known quantity of BSA was added to each probe stock solution to achieve a 5 mM concentration, and the mixture was maintained at 2 to 6 °C. The BSA + site marker binary solution was then titrated with different metal complex concentrations ranging from 0 to 80 μM, while maintaining constant binary solutions of BSA + IBU and BSA + WAR.^55,56^

### 2.10 Density functional theory

Gaussian 16 (G 16W) was utilized to perform electronic structure computations for the new Ti(iv) complexes. The geometries of the titanium(iv) complexes were optimized by applying Lee–Yang–Parr non-local correlation functionals (B3LYP) and Becke's three-parameter hybrid exchange, employing density functional theory (DFT). The DFT and TD-DFT calculations were carried out in the gas phase by using the 6-311G** basis set for lighter atoms (C, H, N, O, F and Cl) and Los Alamos effective core potentials plus the Double Zeta (LanL2DZ) basis set for heavier (Ti and Br) atoms. To ensure that the optimized geometries represent only local minima connected to positive eigenvalues, vibration frequency computations were carried out using the conductor-like polarizable continuum model (CPCM), and vertical electronic excitations based on B3LYP were carried out in DMSO using the time-dependent density functional theory (TD-DFT).^57,58^

### 2.11 Molecular docking studies

When predicting the ideal orientation and precise binding site of metal complexes on macromolecules, particularly those that bind covalently, noncovalently and electrostatically, molecular docking is a highly effective tool for comprehending the interactions between metal complexes and macromolecules. This technique is frequently utilized for the logical design of novel chemotherapy medications and the molecular recognition of nucleic acids.^59^

The software AutoDock Vina and the Lamarckian Genetic Algorithm (LGA) were utilized to establish the binding affinity of the newly synthesized Ti(iv) complexes towards DNA and BSA macromolecules. The stages of molecular docking include protein identification and preparation, ligand preparation and grid generation. Initially, the structural properties of the titanium complexes were optimized using the Gaussian 16W software package in combination with the B3LYP method, incorporating the 6-311+G(d,p) basis set and the LANL2DZ basis set for Ti (including effective core potential).

The 3D X-ray crystallographic structures of the BSA protein (PDB ID: 4F5S) and DNA (PDB ID: 1BNA) were obtained from the RCSB (http://www.rcsb.org/pdb) Protein Data Bank. Using AutoDock techniques, the water molecules from proteins were eliminated. The complexes were then docked with BSA and DNA with defined grid parameters, grid boxes measuring 34.885 Å × 23.976 Å × 98.792 Å and 14.780 Å × 20.976 Å × 8.807 Å, respectively, enclosed BSA and DNA, with a grid spacing of 0.375 Å between each. The docked conformation with the lowest energy was selected for additional examination of the docking simulations, and the outcomes were examined and visualized using BIOVIA Discovery Studio 4.0.^60,61^

### 2.12 DPPH assay

In the current study, 1,1-diphenyl 2-picrylhydrazyl (DPPH) radical scavenging screening determined the antioxidant properties of the synthesized Ti(iv) complexes. The spectrophotometric principle that led to the quenching of stable-colored radicals (DPPH) is the foundation of DPPH assays.^62^ By detecting the drop in absorbance at 517 nm, which corresponds to the DPPH free radical, the scavenging activity of the produced complexes can be determined. The result of the reaction between the scavenger and colored free radical is 1,1-diphenyl-2-picrylhydrazine (DPPH-H), which lacks color. The newly synthesized Ti(iv) complexes were dissolved at a ratio of 1 mg mL^−1^ in DMSO to create the stock solution. Similarly, methanol was used to prepare a stock solution of a 0.05 Mm DPPH solution. The Ti(iv) complexes were dissolved in DPPH stock solution to make (9.5 μM, 18.75 μM, 37.5 μM, 75 μM, 100 μM, and 300 μM) concentrations of the complexes and kept in a state of rest for 40 minutes under closed conditions. The absorbance of the Ti(iv) complexes was measured at 517 nm after 40 minutes for each solution loaded in 96-well plates using a Bio-Rad xMARK™ Microplate Spectrophotometer. For comparison, the absorbance of a control solution containing DPPH and methanol as blank, devoid of test samples, was also measured at the same level. Three sets of experiments were conducted for each concentration, using ascorbic acid as the standard reference. Using eqn (1), the proportion of DPPH free radical scavenging activity was calculated.^63^1
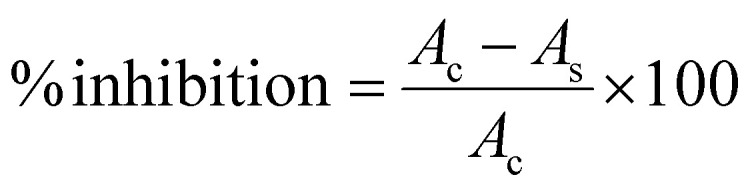
where *A*_c_ and *A*_s_ are the absorbance of the control and the mixture. The necessary concentration of the Ti(iv) complexes to scavenge DPPH radicals was determined by employing linear regression to plot a curve between concentration and % inhibition. The concentration of the sample that could scavenge 50% of the DPPH free radicals was determined by calculating the IC_50_ (half maximal inhibitory concentration) value from the graph for each compound that displayed the critical activity.^64^

### 2.13 MTT assay

The *in vitro* cytotoxicity of the Ti(iv) complexes under investigation was investigated using MTT, a micro-titration colorimetric method. The *in vitro* cytotoxicity test of the Ti(iv) complexes was evaluated against the cervical cancer cell line (HeLa), human breast cancer cell line (MCF-7) and human kidney normal cell line (HEK-293).^65,66^

In the cytotoxicity assay, tetrazolium salt 3-[4,5-dimethylthiazol-2-yl]-2,5diphenyl tetrazolium bromide (MTT) was utilized to assess cell viability. The MTT powder was diluted in Dulbecco's PBS (stock solution of MTT (5 mg mL^−1^)) to attain a final concentration of 0.5 mg mL^−1^ and a 0.25 μM filter was used to filter and sterilize the stock solution, which was then kept at 2–6 °C. In metabolically active cells, MTT is reduced to produce an insoluble purple formazan product. In the exponential phase, cells were taken out of the stored cultures after the 48-hour recovery period, following seeding onto 96-well plates at a density of 1 × 10^4^ cells per well in 100 μL medium at 37 °C in a 95% air and 5% CO_2_ environment, and the cells were incubated for 12 hours. Cell lines were treated for 48 hours with Ti(iv) complexes at varying doses (9.5–300 μM) and *cis*-platin as a control (apart from normal cells). After 48 hours of incubation at 37 °C, the medium was removed and 100 μL of DMSO was added to dissolve the control wells, and the culture plates were shaken for five minutes. A Bio-Rad xMARK™ multi-well plate reader was used to measure the absorbance of each well at 570 nm. Using eqn (2), the percentage viability, which represents the relative viability of the treated cells in comparison to the control cells, was calculated, and the IC_50_ values and standard deviation of the substances under investigation were calculated in triplicate.2
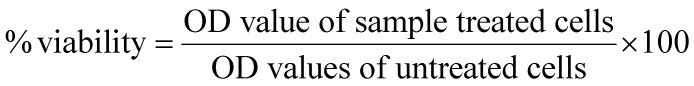


### 2.14 AO–EB staining assay

A dual AO–EB staining fluorescent labelling technique was performed utilizing the Gohel *et al.* protocol, HeLa cells were seeded in 6-well plates at a density of 5 × 10^4^. They were grown in a humidified CO_2_ incubator at 37 °C until they reached 70–80% confluency. Subsequently, IC_50_ concentrations of complexes Ti-3-IO and Ti-5-ICl were used to treat the cells for 48 h. The media were removed from each well, and the cells underwent two gentle room-temperature PBS rinses. Following that, the cells were combined with 100 μL of ethidium bromide and acridine orange dye and incubated separately for 15 minutes at 37 °C after staining separately, and the plates were observed and images were captured with an Olympus Confocal Laser Scanning Microscope—Fluoview Fv3000.^67,68^

### 2.15 Flow cytometry for the detection of cell cycle arrest

A flow cytometer was also used to do the apoptotic assessment. HeLa cells (5 × 10^5^) were seeded onto 6-well plates using Dulbecco's Modified Eagle Medium paper (DMEM) with 10% FBS. The plates were incubated overnight at 37 °C with 95% air and 5% CO_2_. Cells were treated with *cis*-platin at 5 μM with Ti-3-IO and Ti-5-ICl at the appropriate IC_50_ values and then incubated for 48 hours at 37 °C in a CO_2_ incubator. Following trypsinization, the cells were collected, cleaned with PBS, and fixed for 24 hours at 2–6 °C in ice-cold 70% ethanol. Centrifugation was used to remove the ethanol, and cold PBS was used twice to wash the cell pellets. Later the cells were stained with propidium iodide (PI, 1 mg mL^−1^) and incubated for 30 min. Flow cytometric analysis was performed using a FACS scanner and the Becton Dickinson cell search program at 595 nm and the distribution of cells in various cell cycle phases was determined.^69,70^

### 2.16 Generation of reactive oxygen species (ROS)

HeLa cells of density 5 × 10^4^ were seeded in six-well plates with high glucose DMEM and kept overnight until cell confluency was attained. Later, the cells were treated with an IC_50_ concentration of Ti-3-IO and Ti-5-ICl Ti(iv) complexes for 48 hours. The cells were treated with 10 mM 20,70-dichlorodihydrofluorescein diacetate (DCHF-DA) dye after washing twice with PBS buffer. Later, cells were incubated at 37 °C in complete darkness for 30 minutes. Subsequently, an inverted fluorescent microscope (Olympus Confocal Laser Scanning Microscope Fluoview Fv3000) was used to assess the ROS level of the cells. The excitation wavelength was 485 nm, and the fluorescence was observed at 528 nm. The amount of intracellular ROS was expressed as the average fluorescence intensity.^71,72^

## 3 Results and discussion

### 3.1 Synthesis

**Scheme 1:**
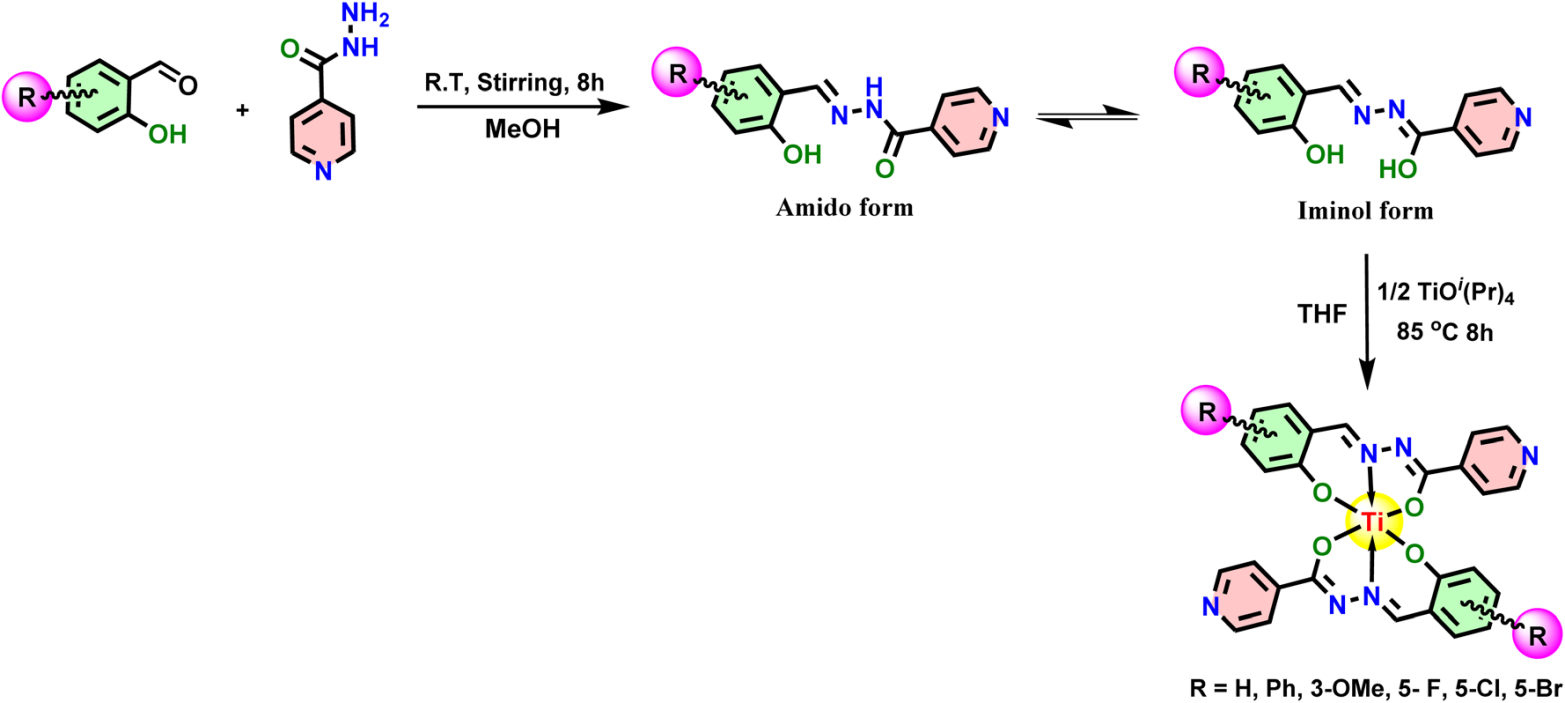
Synthetic route for the synthesis of ligands and Ti(iv) complexes.

The ligand derivatives used in this study, IS, IN, IO, IF, ICl and IBr, were synthesized by slightly altering a previously reported process.^36–40^ These ligands were then characterized using FTIR, ESI-MS, ^1^H and ^13^C NMR spectroscopic techniques and their spectra are made available in ESI.† Then, ligands were treated with a THF (10 mL) solution of titanium isopropoxide (Ti(O^i^Pr)_4_) in a 2 : 1 stoichiometry ratio (Scheme 1) for 8–10 hours to produce the bis-titanium(iv) derivatives (Ti-1-IS to Ti-6-IBr). Following completion of the procedure, the excess solvent was disposed of, to obtain the final product in its neat form as a reddish brown solid in remarkable yield (>75%). The resulting crude product was then washed thrice with hexane and ethyl acetate/hexane (1 : 9) medium.

The newly produced compounds were structurally elucidated using appropriate spectroscopic techniques, such as FTIR, HRMS, and NMR (^1^H and ^13^C), with all spectral data made available in ESI.†

### 3.2 NMR-spectra Ti-1-IS to Ti-6-IBr

**Fig. 2:**
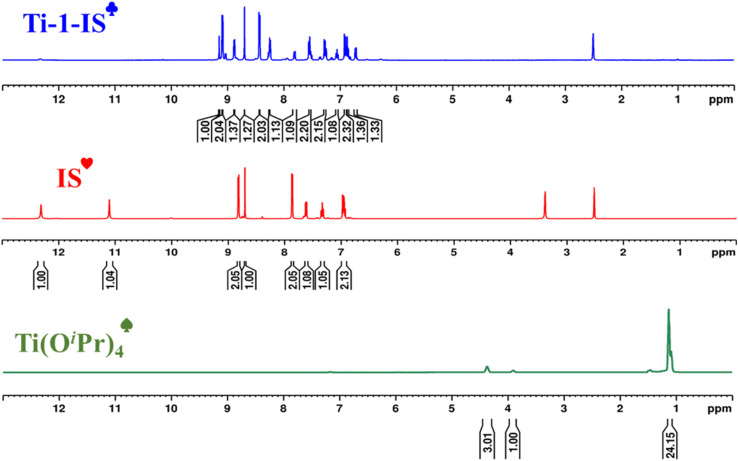
Comparative study of ^1^H NMR spectra of ^♠^Ti(O^i^Pr)_4_, ^♥^ligand IS and complex ^♣^Ti-1-IS.

Proton NMR spectra of Ti-1-IS to Ti-6-IBr showing the disappearance of a couple of free OH and NH protons from the ligands (IS–IBr) in the range of 11.15 and 12.37 ppm provide signature evidence for bond formation between the phenolic oxygen and the central metal, and the conversion of iminol from the amido form of the ligand while participating in complexation with a titanium metal ion. Fig. 2 displays the proton NMR spectra of ^♠^Ti(O^i^Pr)_4_, ^♥^ligand alone (IS) and complex ^♣^Ti-1-IS for comparative study, where the methyl and methylene protons of Ti(O^i^Pr)_4_ were located from 1.17 to 1.18 ppm and from 4.40 to 4.43 ppm. After complexation, isopropoxy groups and free NH and OH protons at 12.37 and 11.15 ppm had completely disappeared, suggesting formation of the complex. Further, the imine protons of Ti-1-IS to Ti-6-IBr were observed as singlet peaks in the range 8.31–10.42 ppm, and the rest of the aromatic protons were found as expected (6.83–8.26 ppm), to authenticate the formation of new complexes.

### 3.3 FTIR spectra of Ti-1-IS to Ti-6-IBr

The dependable vibrational stretching frequencies of these compounds were investigated by comparing the freshly synthesized titanium(iv) derivatives with the genuine bonding pattern of the pertinent free ligands, IS–IBr (ESI Fig. S3, 7, 11, 15, 19, 23†). The establishment of Ti–O bonds in the corresponding derivatives was shown by deprotonation of the wide OH peaks (3141–3221 cm^−1^) ranging from 420 to 460 cm^−1^ and NH peaks (2937–3002 cm^−1^) of the free ligands generating matching new strong peaks in all complexes, which suggests that the ligands are in keto form, since significant stretching frequencies of C=O peaks from the ligand moieties were detected at about 1672–1689 cm^−1^. On the other hand, the carbonyl stretching peak in the FTIR spectra of the complexes shifted towards a lower wavenumber, suggesting that ligands underwent iminol formation by deprotonation of the NH proton to the carbonyl group while building complexes with a Ti^4+^ metal centre. The stretching frequencies of the Ti–N and C–halogen bonds were then found to be at 560–590 cm^−1^ and 697–793 cm^−1^, respectively. The removal of all four isopropoxy moieties from Ti(O^i^Pr)_4_ by coordination with matching ligands to form new complexes was also confirmed by these vibrational spectra. ESI Fig. S27, 32, 37, 43, 48, and 53† display the FTIR spectra of the Ti(iv)complexes.

### 3.4 UV-visible and fluorescence study

A UV-visible experiment was conducted in DMSO and DMSO : water (1 : 9, v/v) media in the 200–800 nm range to determine the photophysical characteristics of the newly synthesized Ti(iv) complexes, (3 × 10^−5^ M). Complexes Ti-C1-IS, Ti-C2-IN, Ti-C3-IO, Ti-C4-IF, Ti-C5-ICl and Ti-C6-IBr displayed two major electronic changes, exhibiting three different peaks, The bands occurring at 240–320 nm can be attributed to the charge transfer that takes place between the ligand π-bonding molecular orbitals and the π* antibonding molecular orbitals, or between the highest occupied molecular orbital (HOMO) and the lowest unoccupied molecular orbital (LUMO). These transitions are referred to as the n–π* and π–π * transitions. Similarly, for the bands occurring in the ranges 340–400 nm and 360–490 nm, respectively, as given in ESI Fig. S56† and Table 1. The three absorption bands visible in the UV-vis spectra of the Ti(iv) complexes correspond the metal-to-ligand charge transfer (MLCT) transition and the ILCT transition, respectively. After being excited at 220 nm, the emission intensities of the Ti(iv) complexes were measured in the 230–500 nm range.^73–75^Eqn (3) was used to calculate the quantum yields of the compounds with the aid of fluorescence data (ESI Fig. S57†)3
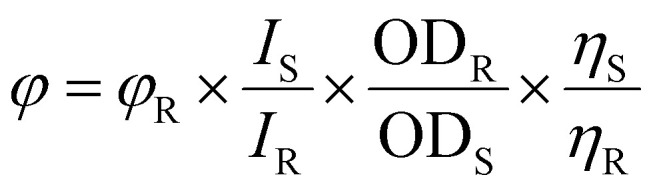
where *φ* = quantum yield, *I* = peak area, OD = absorbance at the *λ*_max_ of the sample (S) and reference (R), *η* = the refractive index of the solvent (S) and reference (R). Ti-5-IBr stands out among them, with a high quantum yield of 0.029 (Table 1).

**Table 1:** Photophysical characterization of all Ti(iv) complexes

Complex	*λ*_max_ a (nm)		*λ*_f_ b (nm)	Stokes shift^c^	OD^d^	*ε* e (M^−1^ cm^−1^)	(*φ*_f_)^f^	Log *P*_o/w_^g^	Λ*M*^h^ (S cm^2^ mol^−1^)	
	π–π*	LMCT							DMSO	10% DMSO
Ti-1-IS	273	333	489	156	0.41	12 500	0.121	0.87	4.5	26
Ti-2-IN	272	436	510	74	1.63	32 500	0.024	0.78	4.9	32
Ti-3-IO	271	340	517	177	2.53	45 200	0.020	0.13	5.2	33
Ti-4-IF	282	340	497	157	3.06	34 150	0.016	0.12	6.5	37
Ti-5-ICl	279	340	514	174	2.31	30 650	0.022	0.31	6.3	35
Ti-6-IBr	278	342	491	149	1.65	24 600	0.029	0.15	7.2	41

a Absorption maxima.

b Wavelength of emission spectra.

c Stokes shift.

d Optical density.

e Extinction coefficient.

f Quantum yield.

g Partition coefficient of *n*-octanol/water.

h Conductance in DMSO and DMSO : H_2_O (1 : 9) media.

### 3.5 Stability studies

Stability studies were carried out using DMSO : H_2_O medium (1 : 9, v/v) and aqueous GSH medium to perform stability tests on the freshly synthesized Ti(iv) complexes over 72 hours. With up to 24 hours of hypochromism in the π–π* and MLCT areas, the Ti(iv) complexes were found to be stable for up to 72 hours of observation, as indicated in Table 1 and ESI Fig. S58 and 59.† Nonetheless, a little deviation was noted in the MLCT region and complexes Ti-3-IO, Ti-4-IF, and Ti-6-IBr displayed a small degree of hyperchromism. For 72 hours, compound Ti-5-ICl remained constant, although complexes Ti-1-IS and Ti-2-IN did not change much.

### 3.6 Solubility, partition coefficient determination (lipophilicity) and conductivity studies

Lipophilicity and hydrophilicity determine the pharmacotherapeutic effectiveness and tumor-inhibiting potential of metal complexes. The solubility studies showed that the Ti(iv) complexes were completely soluble in aprotic solvents (DMSO and DMF), essentially insoluble in chloroform and moderately soluble in protic solvents. These complexes demonstrated a solubility range of 5 mg mL^−1^ in DMSO : H_2_O medium (1 : 9, v/v).

Molar conductivity studies and the ionic character of the Ti(iv) complexes offer valuable insight into the distribution of drugs in the targeted site in a pure and slightly aqueous DMSO environment. In DMSO medium, the Ti(iv) complexes display molar conductivities ranging from 4.5 to 41 S m^2^ M^−1^. Whereas the molar conductance increased in DMSO : H_2_O medium (1 : 9, v/v). The ionization of the Ti and halides associated with the ligands of the complex could account for this rise in conductance. Hence, it is said that their potential for treating cancer cells is undoubtedly demonstrated by their increased conductivity in acidic pH environments. These findings demonstrate the cationic behaviour of the Ti(iv) complexes, which is a crucial characteristic feature for intervening in cancer cells.^76^

Drugs were measured for oral bioavailability and cellular accumulation using their lipophilicity. This can be stated as the log *P* value of the *n*-octanol/water partition coefficient, which is an essential prerequisite for many *in silico* medicinal chemistry approaches. The *n*-octanol/water partition coefficient (log *P*_o/w_), where *P*_o/w_ = the octanol/water partition coefficient, was analyzed using the shake flask method. The experimental log *P*_o/w_ values of these complexes were discovered to be between 0.12 and 0.87 (ESI Fig. S60† and Table 1). The pyridine groups associated with the complexes are hydrophobic, causing the Ti(iv) complexes to exhibit lipophilic properties, among which Ti-1-IS displayed the highest log *P*_o/w_ values.^77^

### 3.7 DNA binding studies

#### 3.7.1 UV-vis abssssorption titrations

DNA is the pioneer transmitter of genetic information. Understanding the pathogenicity or carcinogenesis of novel drugs through the study of interactions between metal complexes and DNA is crucial in drug discovery. Metal complexes interact with double-stranded DNA through different mechanisms. In the current work, we have examined the DNA-binding characteristics of the newly synthesized Ti(iv) complexes. The UV-vis absorption titration method is an electronic spectroscopic techniques that is used in determining the manner of DNA binding to metal complexes.

**Fig. 3:**
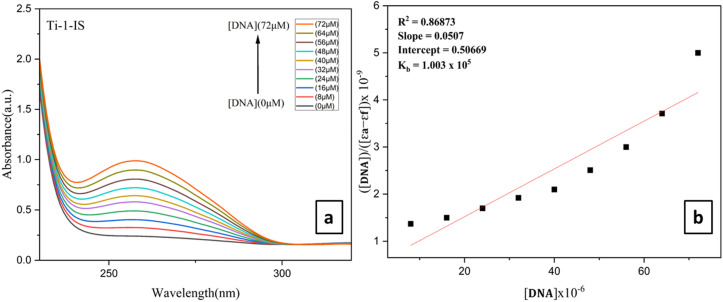
(a) UV absorption titration for Ti(iv) Ti-1-IS with increasing CT-DNA (0–72 μM) concentration in DMSO medium, (b) linear plot of Ti-1-IS.

UV-vis spectra typically show a shift in wavelength and a change in absorption spectra as a result of the interaction of the Ti(iv) complexes with DNA. The UV-vis spectra obtained during the titration of fixed concentrations of the Ti(iv) complexes with increasing concentrations of CT-DNA 5 mM (5 × 10^−5^ M) (0 to 80 μM) are shown in Fig. 3 and ESI Fig. S61.† As DNA is added to the Ti(iv) complexes, their absorption spectra exhibit hyperchromic shifts in both charge transfer zones. As a result, blue shifts of 1–3 nm of the absorption maximum were observed in all the Ti(iv) complexes upon CT-DNA binding and all the complexes exhibited a similar kind of spectral appearance, ranging from 220 to 310 nm. While complexes Ti-4-IF and Ti-5-ICl displayed an isosbestic point supporting an unobstructed view of the binding of the complexes with CT-DNA. These modifications represent the properties of the complexes attached to DNA by covalent or noncovalent interactions. Then, using eqn (4), the binding characteristics for the aforementioned complexes, including binding constant (*K*_b_) and binding sites, were determined.4
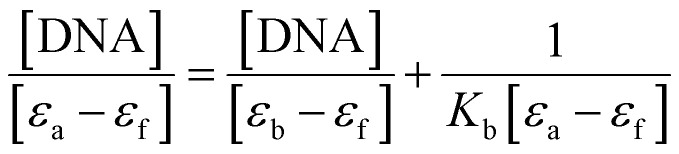
where *ε*_a_, *ε*_f_, and *ε*_b_ represent the apparent extinction coefficient of the complex, the extinction coefficient of the complex in its free form and the extinction coefficient of the complex when completely bound to DNA. Plotting [DNA]/(*ε*_a_ − *ε*_f_) *vs.* [DNA] produced the linear plot displayed in ESI Fig. S62.† The slope-to-intercept ratio was used to determine the *K*_b_ value.

Under these conditions, the CT-DNA and Ti(iv) complexes interact *via* a groove-binding mechanism. The binding constant (*K*_b_) was calculated from the linear plot and it was found that the π* orbital of the complexes coupled with the π* orbital of the DNA base pair, lowering the π–π* transition energy and producing a bathochromic shift (Fig. 3, ESI Fig. S61, 62† and Table 2). On the other hand, hyperchromism is observed if the coupling particle orbits largely due to electrons, enhancing the transition probability. Among all the Ti(iv) complexes, it was observed that Ti-4-IF and Ti-5-ICl exhibited higher *K*_b_ values of 2.97 × 10^5^ and 2.68 × 10^5^, which are displayed in Table 2.

**Table 2:** Binding factors for the CT-DNA interaction with all the Ti(iv) complexes with ct-DNA

Complex	*λ*_max_ (nm)	Change in absorbance intensity	*K*_b_ a (×10^5^ M^−1^)	*K*_SV_ b (×10^6^ M^−1^)	*K*_app_ c (×10^6^ M^−1^)	*n* d
Ti-1-IS	259	Hyperchromism	1.02	0.006	3.03	1.45
Ti-2-IN	258	Hyperchromism	1.94	0.019	3.56	1.06
Ti-3-IO	256	Hyperchromism	1.83	0.016	3.40	1.45
Ti-4-IF	262	Hyperchromism	2.97	0.014	3.41	1.02
Ti-5-ICl	259	Hyperchromism	2.68	0.026	3.43	1.38
Ti-6-IBr	257	Hyperchromism	0.80	0.016	4.24	1.01

a *K*_b_, intrinsic DNA binding constant.

b *K*_SV_, Stern–Volmer quenching constant.

c *K*_app_, apparent DNA binding constant.

d *n*, number of binding sites.

#### 3.7.2 Competitive DNA binding: EtBr fluorescence quenching studies

The popular intercalating agent ethidium bromide, or EB, illustrates the unique property of intercalation by forming soluble compounds with nucleic acids (CT-DNA). An exceptionally intense fluorescence is emitted as a result of the planar phenanthridine ring intercalating between the adjacent base pairs on the double helix structure of CT-DNA. The manner in which metal complexes interact with DNA is frequently investigated using the alterations in EB spectra that occur when it binds to CT-DNA.

**Fig. 4:**
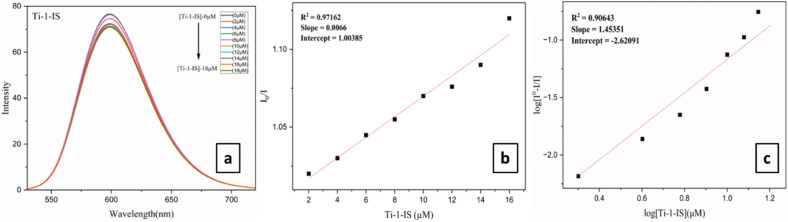
(a) Fluorometric titrations of CT-DNA 5 mM concentration in PBS buffer medium with Ti-1-IS (0–56 μM), (b) Stern–Volmer plot and (c) modified Stern–Volmer/Scatchard plot for Ti-1-IS.

A known volume of 200 μM CT-DNA (5 Mm) and 50 μM EB was mixed with an aqueous PBS buffer to make an EB-CT-DNA solution. The mixture was then kept at room temperature for 20 minutes before the complexes were titrated. Analysis of the competitive interaction mechanisms of the complexes towards CT-DNA involved the incremental addition of Ti(iv) complexes to the mixture of EB-CT-DNA. The modification in the emission spectral profile of EB-DNA indicated the impact of the addition of the complexes and there was a noticeable decrease in inflorescence intensity when the Ti(iv) complexes (0–100 μM) were added to the EB-CT-DNA system (Fig. 4(a), ESI Fig. S63† and Table 2). These decreases suggested that EB and the complexes engage in a competitive relationship for binding to CT-DNA.

The linear plot of *I*_0_/*I versus* [complex] (shown in the inset of Fig. 4(b), ESI Fig. S64† and Table 2) indicated that the quenching of EB-CT-DNA by Ti(iv) complexes was in good agreement with the linear Stern–Volmer eqn (5).

5 *I*_0_/*I* = 1 + *K*_sv_[Q]

where *I* is the fluorescence intensity of the DNA-EB adduct in the presence of the complex (quencher) and *I*_0_ is the fluorescence intensity of the adduct in its absence. *K*_SV_ is the Stern–Volmer quenching constant, and [Q] is the quencher concentration.

6 *K*_app_ × [Complex]_50_ = *K*_EtBr_ × [EtBr]

For the Ti(iv) complexes, *K*_app_ (the apparent binding constant) was calculated using eqn (6), where *K*_EtBr_ (1.0 × 10^7^ M^−1^) is the DNA binding constant of EB, [EtBr] is the concentration of EB = 10 μM and [Complex]_50_ denotes the complex concentration at which the fluorescence intensity of the DNA-EB adduct is reduced to 50%. A substantial affinity of Ti(iv) complexes for CT-DNA was suggested by the *K*_SV_ values, and it was found that Ti-5-ICl exhibited the highest value of 0.026 × 10^6^ M^−1^, which was determined from the slope of the *I*_0_/*I vs.* [Q] plot, *i.e.* a linear plot. Ti-6-IBr exhibited the highest *K*_app_ value of 4.24 × 10^6^ M^−1^ among all the Ti(iv) complexes. The values of *K*_app_ and *K*_SV_ are depicted in Table 2. Furthermore, the number of binding sites (*n*) and the equilibrium binding constant *K* were ascertained by using the Scatchard eqn (7):

7 Log(*I*_0_ − *I*/*I*) = log *K* + *n* log[Q]

where *I*_0_ is the fluorescence intensity of CT-DNA + EtBr in the absence of complex and *I* points to the fluorescence intensities of CT-DNA in the presence of a complex of concentration [Q]. The significant quenching in fluorescence intensity of all metal complexes (Table 2), the Stern–Volmer (ESI Fig. S64†) and Scatchard plots (Fig. 4(c), ESI Fig. S65† and Table 2), and other computations performed, supported the strong groove-binding interaction mode with CT-DNA.

The findings show that Ti(iv) complexes bind to CT-DNA *via* the groove-binding mechanism, as can be seen in Table 2. Among all the other Ti(iv) complexes, Ti-1-IS, Ti-3-IO and Ti-5-ICl show the highest binding, while Ti-4-IF and Ti-5-ICl show the highest intrinsic DNA binding constant bonding values, Ti-5-ICl has the highest *K*_SV_ and Stern–Volmer quenching constant, and Ti-6-IBr has the highest *K*_app_ and apparent DNA binding constant.

#### 3.7.3 Viscosity studies

In addition to the spectroscopic techniques, viscosity measurements were carried out to further clarify the manner of chemical binding of the Ti(iv) metal complexes with CT-DNA. In this case, the viscosity approach makes it easy to identify the change in DNA helix length to which hydrodynamic measurements are typically linked. Basic interactional information, including covalent and non-covalent interaction types, is provided by this method.^45,78,79^

Intercalation, a covalent method of binding, causes the molecule to insert itself into base pairs, lengthening the DNA molecule. This process might also cause the DNA helix to relax, which causes unwinding of the DNA strands. The entering molecule can press into the DNA strands as a result of the helix expanding upwards; in contrast, little change in relative viscosity could be observed in the other interactions, like groove binding where the molecule tries to push its way into the DNA pockets. However, intercalation could result in conformational alterations or hooks, leading to increased stress on the double helical strands of DNA, resulting in a reduction in effective length and a decrease in relative viscosity, which might create twists or conformational changes during intercalation. Whereas, in groove binding it appears that the double helix is bent or twisted in the presence of a groove or other nonclassical interaction, shortening the DNA and decreasing its viscosity.

The viscosity of CT-DNA significantly decreased upon titration of CT-DNA by progressively adding the Ti(iv) complexes, as shown in ESI Fig. S66,† indicating the groove-binding mode since the Ti(iv) complexes split DNA base pairs by inserting between them, shortening the DNA helix and decreasing its viscosity.

#### 3.7.4 DNA interaction study by cyclic voltammetry

Spectroscopic studies and electrochemical investigations are helpful techniques for analyzing interactions between metal complexes and DNA. Using cyclic voltammetry, the binding of Ti(iv) complexes to calf thymus-DNA has been described. Before examining the ability of the complexes to bind DNA, the electrochemical characteristics of a 0.1 M electrolytic solution of Ti(iv) complexes (1 × 10^−3^ M) were assessed in a 0.1 M electrolytic solution of tetrabutylammonium hexafluorophosphate electrolyte. This was done at room temperature using a scan rate of 50 mV s^−1^ across a potential range of −1.5 to 1.5 V at room temperature.

**Fig. 5:**
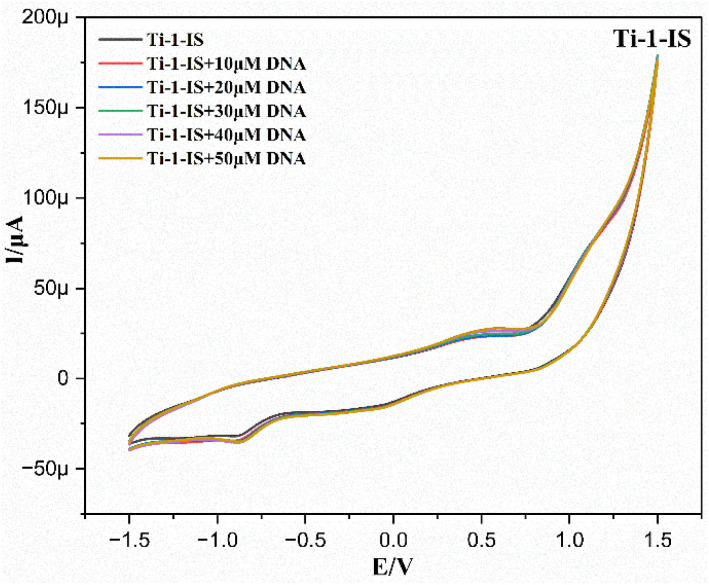
Cyclic voltammograms of Ti-1-IS in the absence and presence of CT-DNA.

Fig. 5 displays the hysteresis that was obtained from cyclic voltammograms of complexes with and without different concentrations of CT-DNA (black curve). The interaction between titanium(iv) derivatives and CT-DNA is demonstrated by the drop in individual peak currents that follows the changes in the anodic and cathodic peak potentials.

The binding of redox-active metal complexes to CT-DNA is understood by monitoring the changes in their electrochemical properties caused by DNA. The Ti(iv) complexes, on the other hand, show redox activity within the potential range under investigation. The characteristic stripping peaks for the complexes appeared at different potentials with both cathodic and anodic peaks, as can be seen in Table S1,† and Fig. 5 shows that the peaks that emerged at +0.058 and +0.75 V were comparable to the oxidation peak of Ti-1-IS at the electrode surface and its reduction peaks at −0.32 and −1.02 V, respectively.

Fig. 5, ESI Table S1 and Fig. S67† revealed that, when CT-DNA (0–80 μM) was titrated with a 0.1 M electrolytic solution of Ti(iv) complexes (1 × 10^−3^ M), there was a minor shift towards the cathodic and anodic currents. Deviation of the peak towards positive potential was also seen, indicating that the complexes preferred groove binding. Similarly, all the Ti(iv) complexes displayed comparable peak types with discrete oxidation and reduction peaks connected to the current flow toward positive potential, indicating groove binding, as displayed in ESI Table S1 and Fig. S67.†

#### 3.7.5 Gel electrophoresis

**Fig. 6:**
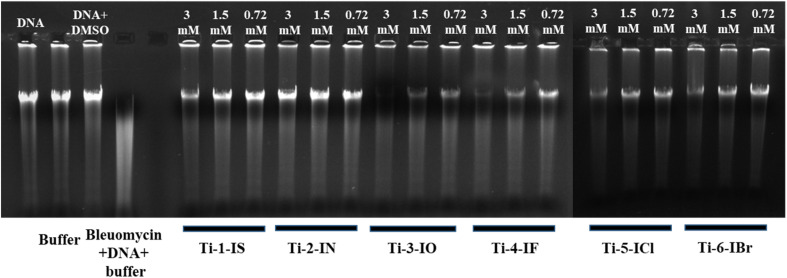
Agarose gel electrophoresis of CT-DNA with Ti(iv) complexes (1) CT-DNA in PBS buffer, (2) CT-DNA in PBS + DMSO, (3) PBS, (4) CT-DNA in PBS + bleomycin, (5) lanes 5–18 CT-DNA + (3, 1.5 and 0.75 mM) Ti-1-IS to Ti-6-IBr.

There are two types of cleavage of nucleic acid chains: enzymatic and non-enzymatic. Either the oxidative cleavage of deoxyribose residues or the hydrolysis of phosphodiester links, known as hydrolytic cleavage, can result in non-enzymatic cleavage. Strong Lewis acidic metal complexes with transition metals are the main source of hydrolytic cleavage. The hydrolysis of the phosphate backbone of the nucleic acid is facilitated by a metal–phosphate intermediate. In order to understand the catalytic mechanisms of natural nucleases and to develop medications that target nucleic acids, it is crucial to design and synthesize metal complexes that can hydrolytically cleave the phosphodiester link. Agarose gel electrophoresis was used to test the capacity of the complex to cleave CT-DNA. The electrophoretic band profile of CT-DNA incubated at various complex concentrations is displayed in Fig. 6. The figure shows that, although a complex concentration of 3 mM damaged the DNA, complex concentrations of 1.5 mM and 0.75 mM produced more than 30% and 15% degradation of CT-DNA, respectively. These findings demonstrated that titanium complexes had a concentration-dependent influence on DNA degradation, and that the nuclease activity potential of the complex was efficient even at low concentrations. Ti-1-IS and Ti-2-IN exhibited less cleavage activity with CT-DNA, Ti-5-ICl and Ti-6-IBr exhibited comparatively higher DNA binding cleavage in higher concentration, whereas Ti-3-IO and Ti-4-IF exhibited higher DNA cleavage even at the lowest concentration, *i.e.* 0.75 mM.

### 3.8 BSA binding studies

#### 3.8.1 BSA binding studies through fluorescence quenching

The fluorescence spectral titration approach is the most efficient method for determining the capacity of a drug to bind to BSA. The addition of metal complexes causes a quenching in the fluorescence spectrum of BSA, primarily caused by conformational and dynamic changes in the protein structure. The tryptophan (Trp) and tyrosine (Tyr) residues in the surrounding polar environment, as well as molecular interactions that quench groups like isoniazid-based Schiff base anions, are responsible for the intrinsic fluorescence intensity of the BSA protein.

**Fig. 7:**
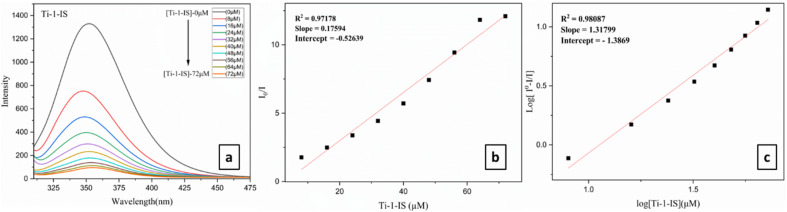
(a) Fluorometric titrations of BSA (3 × 10^−6^ M) concentration in PBS buffer with Ti-1-IS (0–56 μM), (b) Stern–Volmer plot and (c) modified Stern–Volmer/Scatchard plot for Ti-1-IS.

Ti(iv) complexes of (1 × 10^−5^ M) concentration were gradually added, ranging from 0 to 72 μM to bind with BSA (3 × 10^−6^ M) of fixed concentration in PBS buffer of pH 7.4. When the concentration of the Ti(iv) complexes is increased, the fluorescence intensity of BSA is found at 355 nm, which dramatically drops (Fig. 7(a), Table 3 and ESI Fig. S68†) and exhibits a blue shift of about 5–7 nm. This phenomenon might be caused by modifications to the secondary or tertiary structures of BSA, which could have an impact on the tryptophan residues of the BSA microenvironment. Typically, the Stern–Volmer eqn (8) can be used to characterize fluorescence quenching.

8 *I*_0_/*I* = 1+*K*_BSA_[Q] = 1 + *k*_q_*τ*_0_[Q]

where *τ*_0_ is the lifetime of the tryptophan in BSA, which is determined to be 1 × 10^−8^, *k*_q_ is the quenching constant, *I*_0_ is the fluorescence intensity of BSA in the absence of complex and *I* denotes the fluorescence intensities of BSA in the presence of a complex of concentration [Q]. The quenching rate constant, dynamic quenching constant, average lifetime without quencher, and quencher concentration of the biomolecule are represented by the values of *k*_q_, *K*_BSA_, *τ*_0_ and [Q], respectively. Of all Ti(iv) derivatives Ti-3-IO exhibited the best *k*_q_ (2.77 × 10^13^ M^−1^ s^−1^) and *K*_BSA_ (0.277 × 10^6^ M^−1^) values (Fig. 7(b), Table 3 and ESI Fig. S69†).

**Table 3:** Binding factors for BSA interaction with Ti(iv) complexes

Complex	*K*_BSA_ a (×10^6^ M^−1^)	*K*_q_ b (×10^13^ M^−1^ s^−1^)	*K*_b_ c (×10^4^ M^−1^)	*n* d
Ti-1-IS	0.175	1.75 × 10^13^ M^−1^ s^−1^	0.040	1.31
Ti-2-IN	0.105	1.05 × 10^13^ M^−1^ s^−1^	0.047	1.26
Ti-3-IO	0.277	2.77 × 10^13^ M^−1^ s^−1^	0.036	1.42
Ti-4-IF	0.197	1.97 × 10^13^ M^−1^ s^−1^	0.011	1.60
Ti-5-ICl	0.112	1.12 × 10^13^ M^−1^ s^−1^	0.065	1.07
Ti-6-IBr	0.085	0.85 × 10^13^ M^−1^ s^−1^	0.014	1.35

a *K*_BSA_, Stern–Volmer quenching constant.

b *K*_q_, quenching rate constant.

c *K*_b_ binding constant.

d *n*, number of binding sites.

The binding constant (*K*_b_) and the number of binding sites (*n*) for the static quenching interaction can be determined using the Scatchard eqn (9),

9 Log(*I*_0_ − *I*/*I*) = log *K* + *n* log[Q]

where *I*_0_ and *I* are the fluorescence intensities of BSA with and without quenchers (complexes). Fig. 7(c), Table 3 and ESI Fig. S70† display the double logarithmic plot of log[*I*_0_ − *I*/*I*] *vs.* log[complex]. The values of *K*_b_ and *n* at 298 K were calculated for the Ti(iv) complexes with BSA and it was found that complex Ti-5-ICl exhibited the highest *K*_b_ (0.046 × 10^6^ M^−1^) value and Ti-4-IF exhibited the highest *n* (1.60) value.

#### 3.8.2 BSA binding studies through synchronous fluorescence quenching

Investigating the interactions between Ti-1-IS to Ti-6-IBr complexes and BSA under physiologically representative conditions is the goal of this study. The number of binding sites, binding constants, and quenching processes were measured using fluorescence analysis. The findings facilitate investigation into the transport, processing and certain significant bioactivities of protein-carrying drug molecules, as well as how they get to their intended sites in the human body without losing their pharmacological characteristics.

**Fig. 8:**
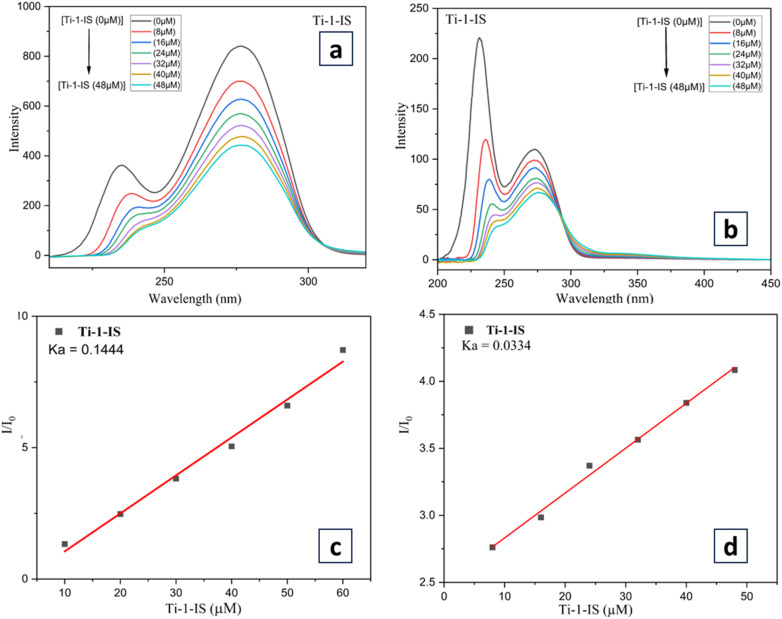
Synchronous titrations of BSA (1 × 10^−5^ M) with Ti-1-IS complex of concentration (0–56 μM); (a) at Δ*λ* = 15 nm; (b) at Δ*λ* = 60 nm at 298 K; (c) and (d) are the Stern–Volmer plots for Ti-1-IS at Δ*λ* = 15 nm and 60 nm.

In synchronous fluorescence spectroscopy, the wavelength difference between excitation and emission (Δ*λ* = *λ*_emi_ − *λ*_exc_) represents the spectra of chromophores of various natures. Depending on the chromophore type, Ti(iv) complexes are gradually added to BSA in this investigation using fixed Δ*λ* (*λ*_emi_ − *λ*_exc_). Tryptophan exhibits synchronous fluorescence at Δ*λ* = 60 nm, while the tyrosine residue exhibits synchronous BSA fluorescence at Δ*λ* = 15 nm. The fluorescence intensity of tyrosine at 275 nm and tryptophan at 279 nm was reduced upon the incorporation of complexes, as can be seen in Fig. 8a, b, ESI Fig. S71 and S73.†

The amplitude of emission corresponding to tyrosine (at 295 nm) was observed to drop moderately (10 to 62% for all complexes) without a change in emission wavelength when the Ti(iv) complexes were titrated incrementally (0–48 μM) with BSA (3 × 10^−6^ M) as constant. With negligible changes in the emission wavelength, an immoderate drop in fluorescence emission was observed in the intensity (at 340 nm) of around 50 to 80% for all new complexes of Ti(iv) when BSA (3 × 10^−6^ M) was kept constant with incremental addition of the titanium(iv) complexes (0–48 μM), as displayed in Fig. 8a, b, S71 and S73.†

The results above demonstrated unequivocally how the metal complexes affected the polarity and microenvironments of the tryptophan and tyrosine residues throughout the binding process. The hydrophobicity shown in fluorescence and synchronous measurements confirmed that all the Ti(iv) complexes are effectively bound with BSA and the binding site *K*_a_ was obtained from Stern–Volmer plots for these complexes, which were plotted from the data obtained from fluorescence data that are highlighted in Fig. 8c, d, ESI Fig. S72, 74 and Table S2.†

#### 3.8.3 BSA site marker fluorescence quenching studies

The heart-shaped protein known as bovine serum albumin is separated into three domains (I–III), each of which has two subdomains (A and B). Transition metal complexes can attach to the BSA molecule at two different locations: the subdomain IIA site and/or the subdomain IIIA site. The site marker fluorescent probes, namely warfarin (site I) and ibuprofen (site II) were incorporated in the site marker displacement assay to identify the competitive binding site of metal complexes inside BSA.

In our experiment, fluorescence titration techniques were employed to determine the binding position of the BSA molecule. For the fluorescence titration, the excitation wavelength was 295 nm, and the emission was monitored between 300 and 500 nm where a fixed concentration of BSA (3 × 10^−6^ M) and site markers (BSA + WAR) (BSA + IBU) were taken separately and titrated with the incremental addition of Ti-1-IS to Ti-6-IBr (0–100 μM). By comparing the Ksv values shown with and without site markers, the binding site of Ti(iv) complexes inside BSA was identified (ESI Table S3†).

The fluorescence intensity of the BSA solution decreases upon the addition of the site marker, indicating that the site marker has attached to the BSA molecule. When the complex is introduced to the site marker–BSA solution, it must compete with the appropriate marker if it binds to the same site to bind to BSA. When compared to the *K*_SV_ values in the absence of the site marker, the value changes significantly as a result of the complex and the site marker competing for the same binding site.

The Scatchard eqn (9) was used to examine the emission quenching data. ESI Table S3† shows that the presence of the site markers ibuprofen and warfarin has the greatest impact on the binding constant. According to the findings of this experiment, each complex under investigation has a single binding site on the BSA molecule; hence, the complexes can attach themselves to either site I or site II of the BSA molecule, but not both. The inclusion of ibuprofen and warfarin as site markers significantly alters the binding constant for complexes Ti-1-IS to Ti-6-IBr, as can be seen in ESI Table S3,† with the constant value being almost doubled.

**Fig. 9:**
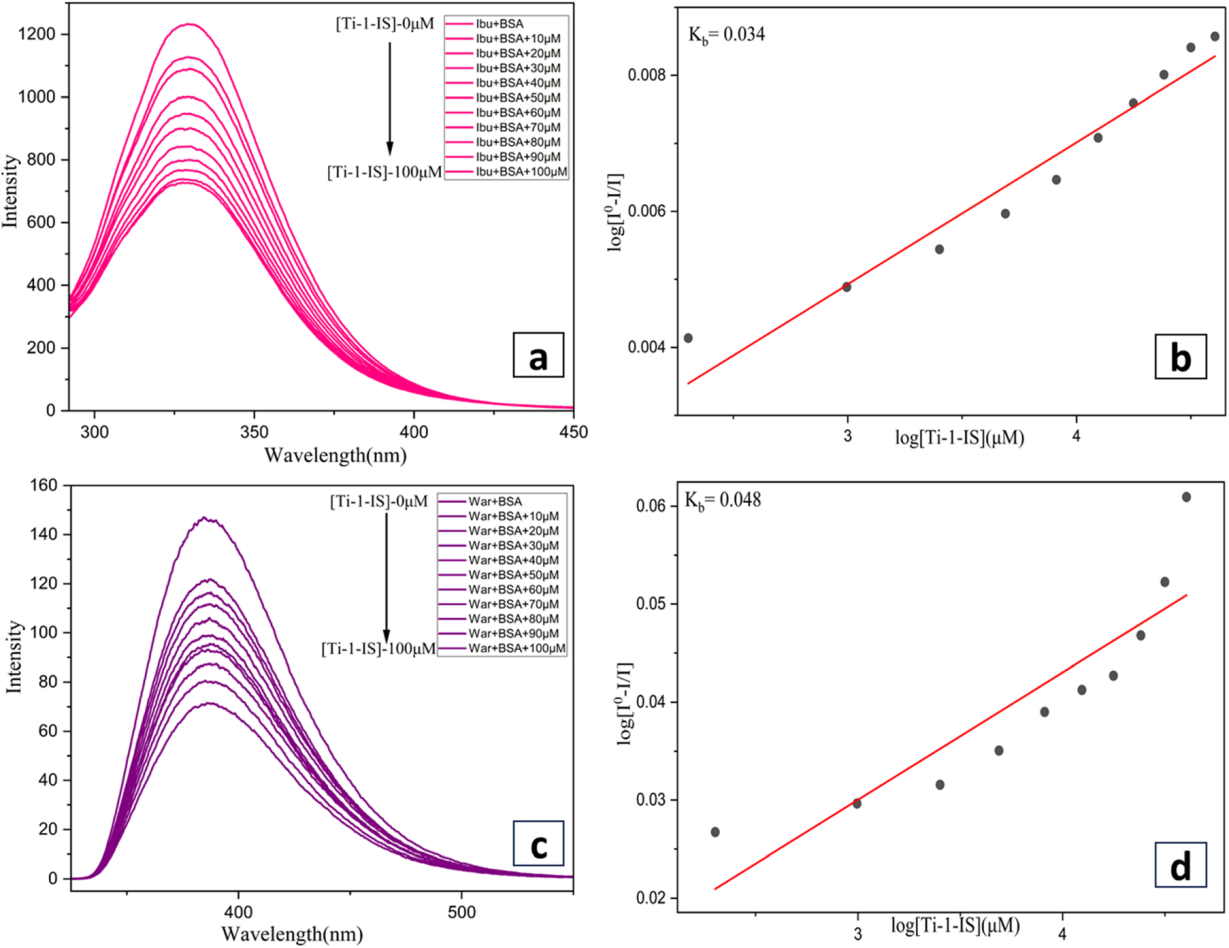
(a) Site marker study of BSA + ibuprofen (1 × 10^−5^ M) with Ti-1-IS complex of concentration (0–56 μM); (b) double log plot for BSA + ibuprofen with Ti-1-IS; (c) BSA + warfarin (1 × 10^−5^ M) with Ti-1-IS of concentration (0–56 μM); (d) double log plot for BSA + warfarin with Ti-1-IS complex.

However, there is only a minor change in the *K*_SV_ value when ibuprofen is present. These findings suggest that site I of the BSA molecule should be the primary location for the binding of the complexes under investigation. In order to bind to BSA, the complexes under investigation must compete with ibuprofen (Fig. 9(a) and ESI Fig. S75†). However, it was not possible to completely rule out a little variation in the constant value when warfarin (Fig. 9(b) and ESI Fig. S77†) was present. The *K*_SV_ values for both WAR + BSA and IBU + BSA are given in Table S3† and their Scatchard plots are displayed in Fig. 9c, d, ESI Fig. S76, S78 and Table S3.†

Docking studies for BSA were conducted to determine their binding efficacy. It is clear from the experimental and docking data that the complexes under investigation attach to the site I of the BSA molecule. Although the hydrophobic interaction is the most likely cause of this binding, other interactions, including electrostatic, hydrogen bonding, van der Waals interactions, and steric interactions, cannot be completely ruled out.

### 3.9 Molecular docking with DNA and BSA

**Fig. 10:**
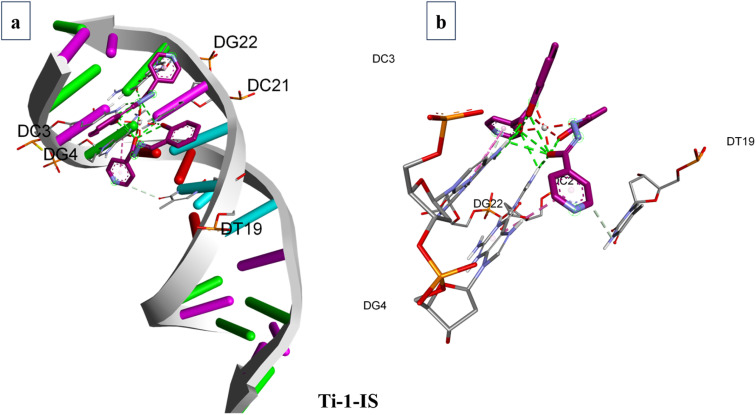
Binding sites of Ti-1-IS with (a) DNA (b) highlighted molecule; purple indicates carbon atoms, blue indicates nitrogen atoms, grey indicates titanium(iv) ions, and red indicates oxygen atoms.

Molecular docking is a preliminary computational approach for determining appropriate locations for the interaction of Ti(iv) complexes with DNA and BSA. Specifically, we aim to examine the degree to which the new titanium complexes might bind to BSA (PDB ID: 4F5S) and DNA (PDB ID: 1BNA) dodecamer ACCGACGTCGGT,^80,81^ by modifying the DNA active sites. In the docking calculations, the DNA structure was restricted to being rigid, but the geometry of the complexes was deemed to be variable. For the docking computations, the Lamarckian Genetic Algorithm (LGA) was employed and a grid box with *XYZ* dimensions of 14.780 Å × 20.976 Å × 8.807 Å was created with a grid point spacing of 0.375 Å. AutoGrid was used to construct electrostatic and affinity maps of the current atoms. By means of AutoDock Vina software,^67^ free energies of binding for the Ti(iv) complexes were calculated based on these findings and came out to be in the range of −8.4 to −10.3, and it was found that Ti-1-IS exhibited the highest binding energy. The binding site and molecular docking of the complexes interacting with DNA are displayed in Fig. 10 and ESI Fig. S79–84.†

Ti-2-IN and Ti-1-IS possess more hydrogen-bond acceptors than their fellow complexes and due to their bulkiness, they could not exhibit higher binding properties with higher binding energy, stronger hydrogen bonding or the highest degree of flexibility. Notably, Ti-4-IF, Ti-5-ICl and Ti-6-IBr have the phenyl-moieties supported by halogen substituents in a location that can support stacking interactions (SI), yet all the Ti(iv) complexes exhibit groove binding. Despite having two SI, in Ti-4-IF, Ti-5-ICl and Ti-6-IBr, several atoms are stretched apart from the double coils. Compared to the halogen atoms in Ti-4-IF, Ti-5-ICl and Ti-6-IBr, Ti-3-IO possessing methoxy atoms appears to be less involved in the bonding compared to their fellow complexes. It is tempting to argue that none of the Ti(iv) complexes looks to be at ease within the active site; rather, they seem to be fleeing rather than settling into the double strand, allowing them to participate in groove binding.

The binding poses, docking scores and binding amino acids of DNA to Ti-1-IS to Ti-6-IBr are highlighted in Fig. 10, ESI Table S4 and Fig. S79–84.†

**Fig. 11:**
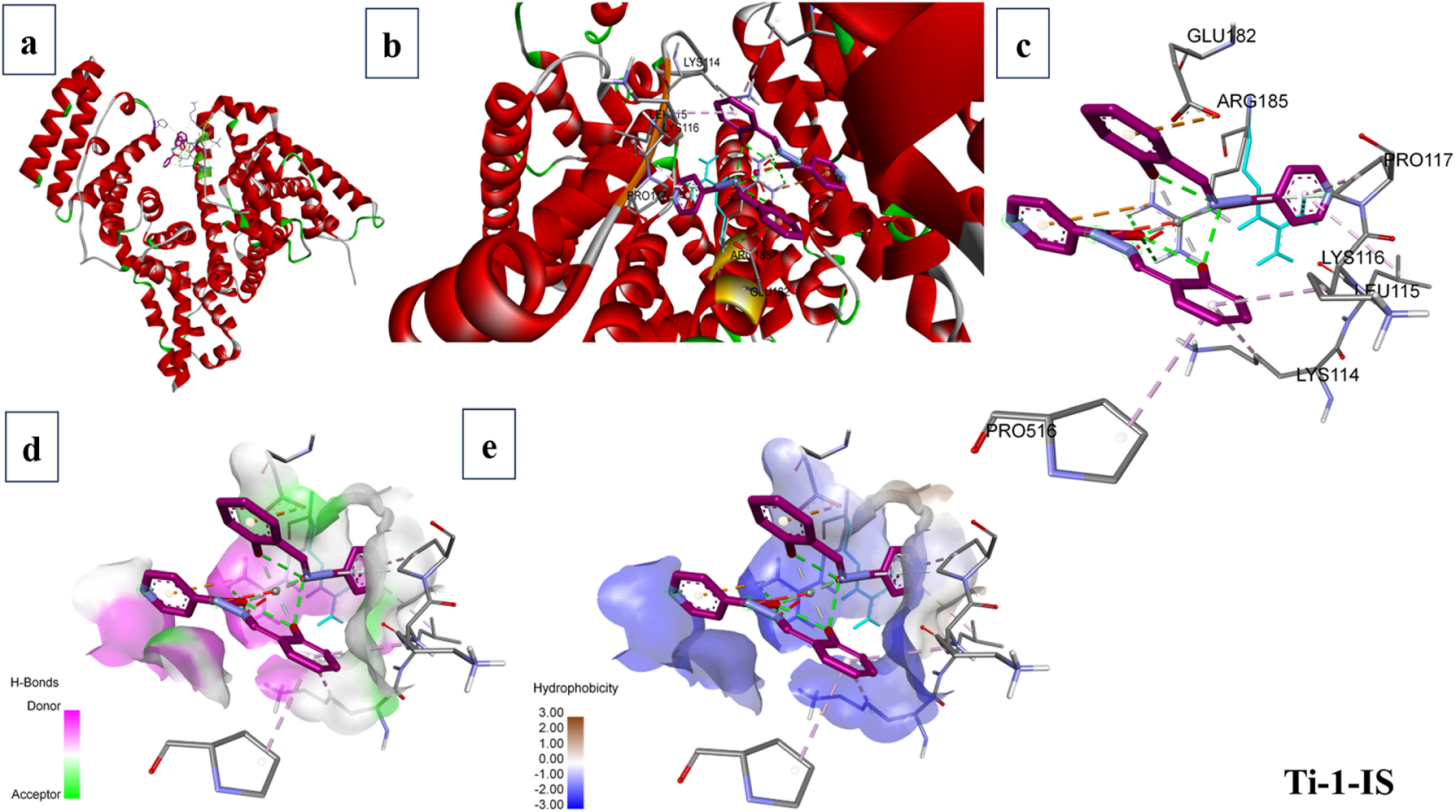
Binding sites of Ti(iv) complex TI-1-IS with BSA; (a) binding site on BSA; (b) elaborated binding site; (c) expanded view of interactions; (d) hydrogen bonding around the complex and (e) hydrophobicity around the complex; purple indicates carbon atoms, blue indicates nitrogen atoms, grey indicates titanium(iv) ions, red indicates oxygen atoms.

The three primary domains of BSA are I, II, and III. Among them, the residues of domain II are the zones with the greatest electrostatic surfaces. By keeping the coordinates of the central grid point fixed at *x* = 34.885, *y* = 23.946, and *z* = 98.792 with a grid point spacing of 0.375 Å, the complexes were docked with BSA. The two significant BSA binding sites are close to Trp134 and Trp213, which are on the surface of the hydrophilic region while Trp213 is a component of a hydrophobic site. All of the produced Ti(iv) complexes fill the anticipated binding sites, as the docking energies clearly show their binding specificity. The hydrophobic pharmacophores of each complex are located close to Trp134 (Fig. 11 and ESI Fig. S85–S90†), in line with the observation that all of the compounds have high affinity for BSA.

When the Ti(iv) complexes underwent molecular docking with BSA, their docked scores were found to be −9.2 to −11.5, indicating the formation of stable protein–ligand complexes. It is evident from Fig. 11 and ESI Fig. S85–S90† that Ti-1-IS to Ti-6-IBr bind adjunctly to sites IA and IIIB. The numerous noncovalent interactions that take place between the produced complexes and the amino acid residues of BSA stabilize the docked structures.

Ti-2-IN exhibited the highest docking score among the complexes and it was found that Ti-1-IS also, in a slight way, displayed a similar docking score to Ti-2-IN, whereas all the halogen-substituted complexes *i.e.*Ti-4-IF, Ti-5-ICl and Ti-6-IBr, revealed appreciable docking scores, and Ti-3-IO exhibited the lowest docking score. All docking scores, binding amino acids and docking poses are displayed in Fig. 11, ESI Fig. S85–S90 and Table S5.†

### 3.10 DFT studies

In order to plot the 3D structure of the Ti(iv) complexes and investigate their molecular insights and photophysical properties, a computational investigation was carried out employing the Gaussian 16 (G 16W) computational program. These complexes exhibit a variety of electronic excited-state configurations, including metal-to-ligand charge transfer (MLCT) and ligand-to-metal charge transfer (LMCT).

Numerous quantum-chemical properties, such as geometric optimization, electrostatic potential (ESP) charges, the energy of frontier molecular orbitals, the bandgap, and molecular descriptors, were determined.

Time-dependent density functional theory (TD-DFT) and vertical electronic excitations based on B3LYP were generated to depict the local minima connected to positive eigenvalues to determine the geometry of the Ti(iv) complexes tuned to zero negative vibration frequency. Using ground-state optimized geometry, both DFT and TD-DFT investigations were linked to the conductor-like polarizable continuum model (CPCM) in a dimethyl sulfoxide (DMSO) medium to consider the impact of the solvent.

**Fig. 12:**
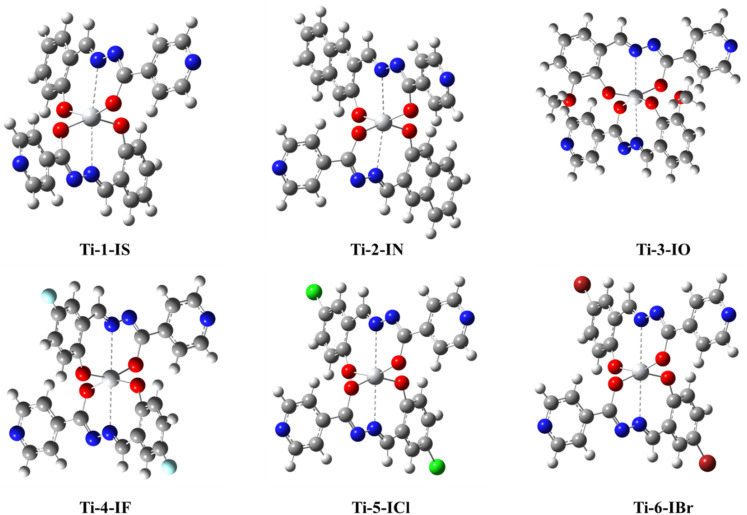
Optimized structures of Ti(iv) complexes by B3LYP and LANL2DZ methods; dark grey indicates carbon atoms, blue indicates nitrogen atoms, light grey indicates titanium(iv) ions, red indicates oxygen atoms and white indicates hydrogen atoms.

The combined DFT-B3LYP method with the Gaussian 16 computational tool was used to conduct computational studies of synthesized isoniazid-based Schiff base derivatives and their Ti(iv) complexes. Utilizing B3LYP/6-31G**/LanL2DZ ECP techniques, many quantum-chemical parameters were computed, including bandgap, molecular energy, ESP charges, frontier molecular orbital energy, and geometry optimization. The corresponding critical bond lengths for all of the Ti(iv) complexes proved that an octahedral geometry had been established, as shown in Fig. 12 and ESI Table S6.†

**Fig. 13:**
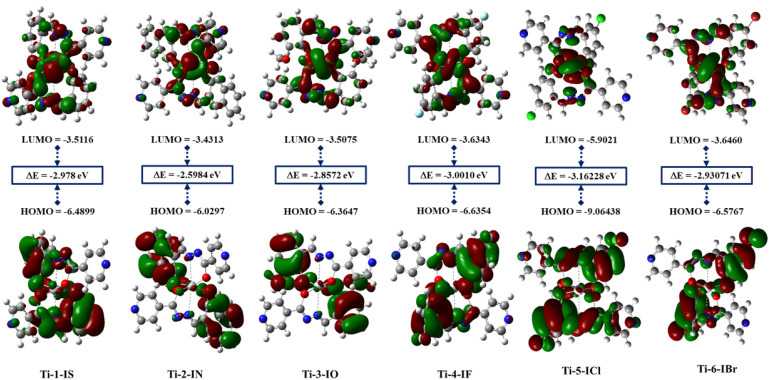
Frontier molecular orbitals and energy gap (Δ*E*) of the Ti(iv) complexes.

The frontier molecular orbitals (FMOs) were compared and the HOMO and the LUMO with the energy gap (Δ*E*) of complexes Ti-1-IS to Ti-6-IBr were computed and are displayed in ESI Fig. S44.† All complexes exhibited the bandgap energies in the range from 2.59 to 3.16 eV. The HOMO–LUMO energy gaps (Δ*E*) of chloro- and fluoro-substituted complexes (Ti-5-ICl and Ti-6-IBr) are higher than for the other derivatives. The chemical reactivity and kinetic stability of the complexes can be determined from the energy gap (Δ*E*). Energy gaps indicate that charge transfer occurs easily within them, whereas the biological activity of the complexes influences them. Fig. 13 and Table 4 list the *E*_HOMO_ and *E*_LUMO_, energy bandgap (Δ*E*), energies of global softness (*S*), global hardness (*η*), global electrophilicity index (*ω*), electronegativity (*χ*), chemical potential (*μ*), global hardness and global electrophilicity index (*ω*), which were calculated by employing eqn (10)–(15). These factors are crucial for determining the stability and reactivity of a molecule. Furthermore, the biological activity of the drug–receptor interaction is quantified with the electrophilic nature of the complexes.

10 Δ*E* = *E*_LUMO_ − *E*_HOMO_

11
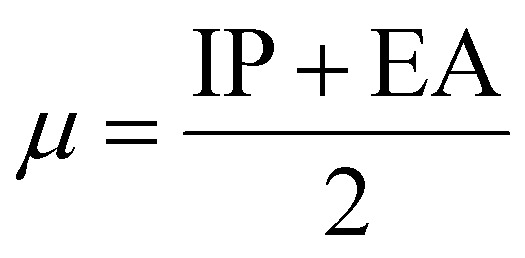
12
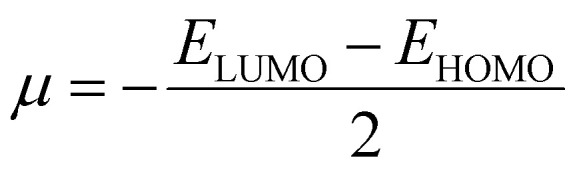
13
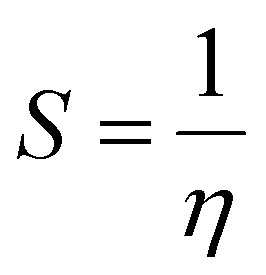
14
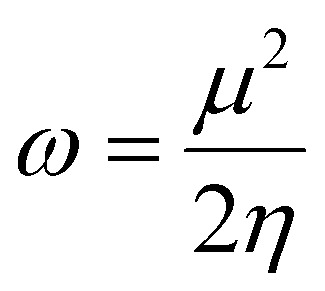

15 *χ* = −*μ*

**Table 4:** Calculated molecular electronic parameters of the Ti(iv) complexes

S. no.	Code	Energy (kcal mol^−1^)	DM	HOMO (eV)	LUMO (eV)	Δ*E* (eV)	*χ* (eV)	*μ* (eV)	*η* (eV)	*S* (eV)	*ω* (eV)
1	Ti-1-IS	2 610 725.93	7.09	−6.48	−3.51	2.97	4.99	−4.99	−1.48	−0.74	−18.42
2	Ti-2-IN	2 826 128.51	9.74	−6.02	−3.43	2.59	4.70	−4.70	−1.29	−0.64	−14.24
3	Ti-3-IO	4 156 258.55	9.07	−6.36	−3.50	2.85	4.93	−4.93	−1.43	−0.71	−17.37
4	Ti-4-IF	2 601 096.44	8.44	−6.63	−3.63	3.00	5.13	−5.13	−1.50	−0.75	−19.73
5	Ti-5-ICl	2 635 716.93	5.76	−9.06	−5.90	3.16	7.48	−7.48	−1.58	−0.79	−44.20
6	Ti-6-IBr	2 561 412.08	5.91	−6.57	−3.64	2.93	5.10	−5.10	−1.46	−0.73	−18.98

**Fig. 14:**
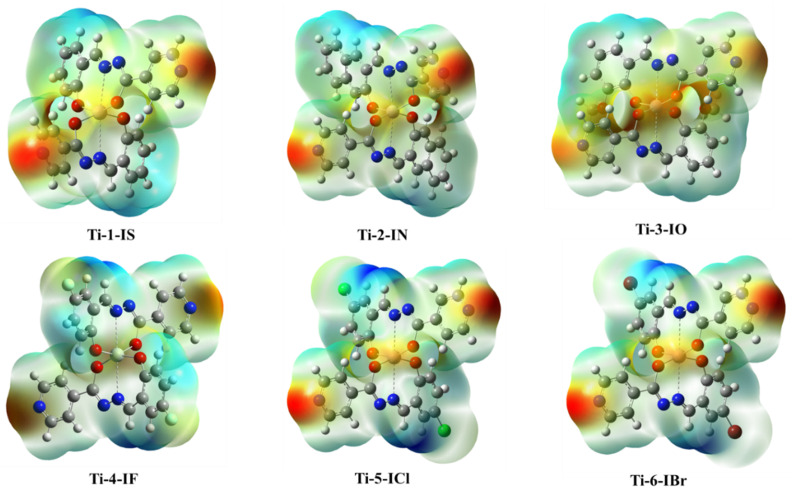
Electrostatic potential (ESP) surfaces of the Ti(iv) complexes.

The electrostatic potential mapped on the constant electron density surface for the ideal geometry on the van der Waals surface was very helpful in studying the hydrogen-bonding interactions in the Ti(iv) complexes and the relationship between photophysical properties and molecular structure. The red region indicates the most negative region, which is the place of choice for electrophilic attack. The optimal location for nucleophilic attack, the most positive zone, is shown in the blue region in Fig. 14.

**Fig. 15:**
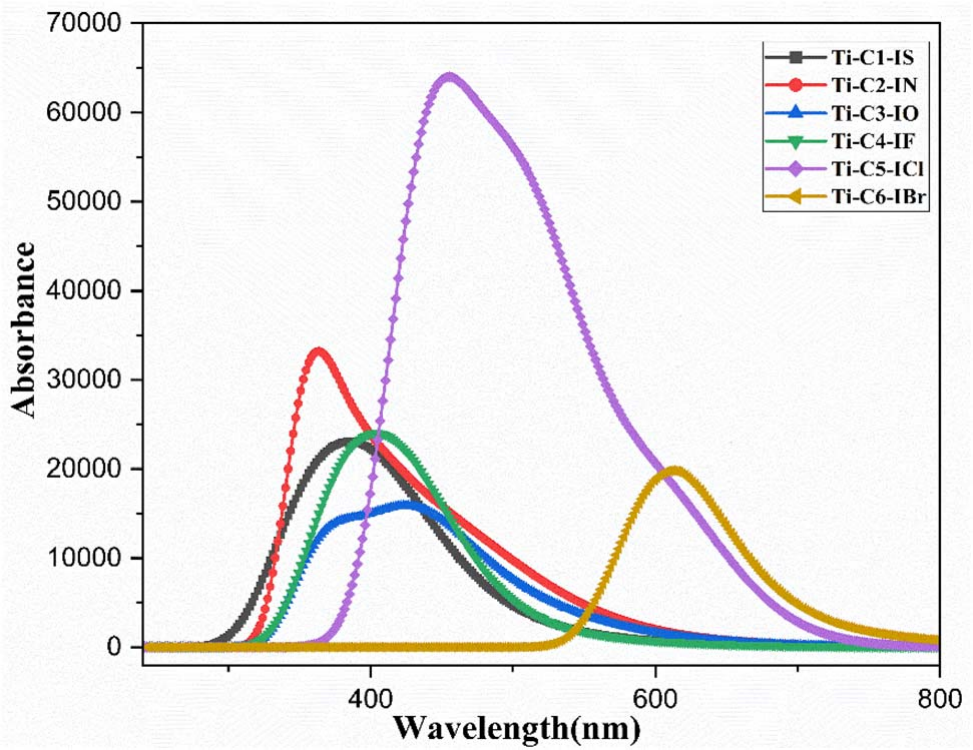
UV-vis spectra of the Ti(iv) complexes obtained from TD-DFT calculations.

In the TD-DFT calculations, ground-state optimized geometries were used, and the accompanying electronic transitions along with the related orbital contributions were calculated. The Ti(iv) complexes displayed a minor charge transfer band at about 500 nm and strong electronic bands occurred in the range of 350 to 370 nm for the strong π → π* transition. In contrast, Table 7 shows the anticipated and measured absorbance bands that matched the distinctive electronic transition. Furthermore, Fig. 15 and ESI Table S7† display the anticipated UV-vis spectra of the Ti(iv) complexes, which coincide with the observed absorbance band.

### 3.11 Structure–activity relationship (SAR)

**Fig. 16:**
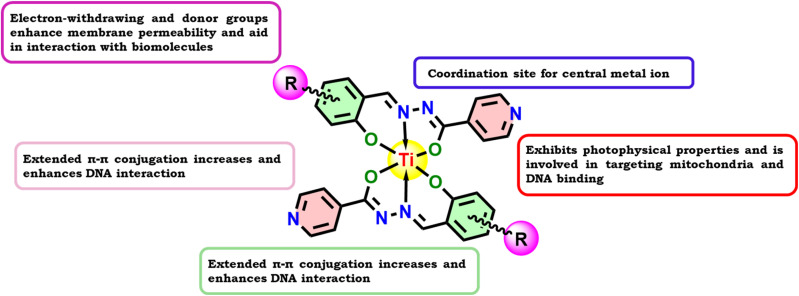
Representation of plausible structure–activity relationship of the Ti(iv) complexes.

The structure–activity relationship shows that the cytotoxic profiles of the complexes in tested cancer cell lines were notably high when they were substituted with various electron-donating groups, such as methoxy, and electron-withdrawing groups, such as chloro groups. Rapid ligand exchange is made possible by these lipophilic, interchangeable ligands, which might facilitate binding with biomolecules due to the ligand planar aromatic moiety. The presence of the (Ti^4+^) central metal ion made them appropriate for DNA binding, that in turn caused destruction of DNA replication, as displayed in Fig. 16, followed by apoptosis, aiding in targeting mitochondria as it coordinates with the negatively charged phosphate backbone of DNA base pairs. The hydrophobicity was enhanced by the aromatic rings of the ligands, which demonstrated π–π interaction with CT-DNA by the groove-binding mode. Fluorescent characteristics are magnified for cellular tracking using a long-term π-electronic approach. Because of its strong cell permeability and DNA binding sites, Ti-5-ICl outperformed other compounds in terms of selectivity against HeLa and MCF7 cell lines.

Ti-3-IO and Ti-5-ICl displayed nearly identical activity towards HeLa cell lines, according to a thorough examination of the cytotoxicity of the Ti(iv) derivatives; nevertheless, a steric effect might be responsible for the reduction in the interaction with the charged groups of CT-DNA.

### 3.12 DPPH assay

In order to ascertain the antioxidant activity of the Ti(iv) complexes, a UV-vis spectrophotometric examination was performed using the 1,1-diphenyl-2-picrylhydrazyl (DPPH) method. Since DPPH is thought to be a dependable, stable, free radical, it reacts with methanol to form a red solution. The ability of the complexes containing DPPH radicals to scavenge radicals was evaluated in the manner described. Several concentrations of the Ti(iv) complexes were investigated. Mixtures created by mixing DPPH with each component were subjected to spectrophotometric analysis at 517 nm to determine the absorbance. Antioxidant compounds usually transfer one electron to act as a reducing agent.

Ascorbic acid was the reference used in the current *in vitro* study; thus the antioxidant potential of ascorbic acid and the Ti(iv) complexes was investigated. Each test was conducted in triplicate to ascertain the percentage of DPPH radical scavenging activity. The results of the DPPH assay, *i.e.* calculated IC_50_ values, are listed in Table 5 and ESI Fig. S91.†Ti-3-IO exhibited a lower IC_50_ value than its fellow compounds. The lower the IC_50_ value of a compound, the higher its antioxidant activity, proving that Ti-3-IO is a strongly antioxidant agent.

**Table 5:** IC_50_ values from the DPPH assay for the synthesized Ti(iv) complexes

S. no.	Code	IC_50_ values
1	Ti-1-IS	92.01 ± 0.1
2	Ti-2-IN	128.05 ± 0.3
3	Ti-3-IO	44.59 ± 0.4
4	Ti-4-IF	82.61 ± 0.1
5	Ti-5-ICl	72.26 ± 0.7
6	Ti-6-IBr	74.84 ± 0.2

### 3.13 MTT assay

An MTT assay was used to examine the cytotoxic effectiveness of the produced Ti(iv) complexes. After 48 hours of incubation, the cytotoxicity of Ti-1-IS to Ti-6-IBr was evaluated against human cancer cell lines, such as breast carcinoma (MCF7) and cervical cancer (HeLa), as well as the non-cancerous human embryonic kidney (HEK) cell line, by using the widely used therapeutic drug, *cis*-platin, as a positive control by following the standard protocol.

**Fig. 17:**
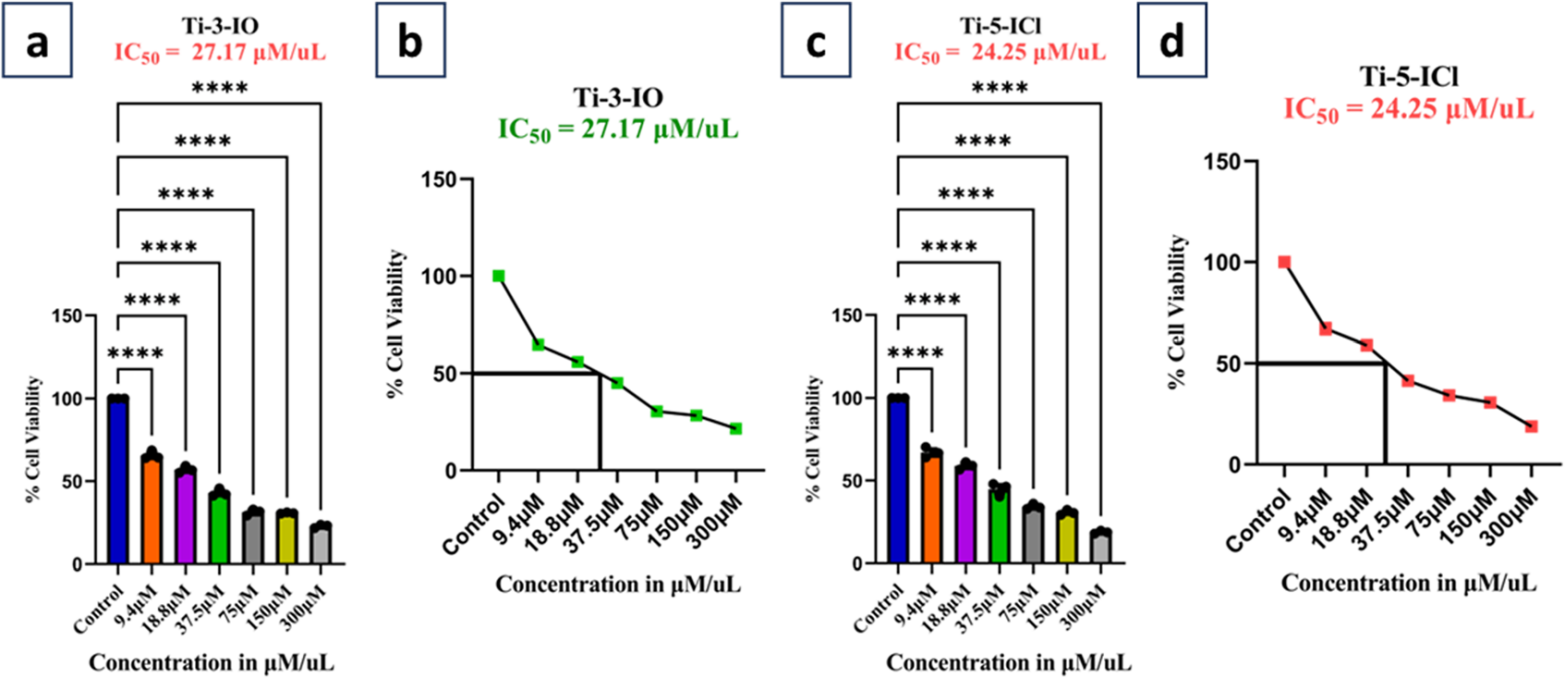
MTT assay and IC_50_ value determination: cell inhibition (%) of HeLa cell line with Ti-3-IO (a and b) and Ti-5-ICl (c and d). All values are expressed as mean ± SEM. ****p < 0.0001 denotes statistical significance as determined by the MTT assay. Error bars indicate the standard deviation from three independent experiments.

The MTT assay was conducted by treating 9.4 to 300 μM of the Ti(iv) complexes with MCF and HeLa cell lines. It was observed that all complexes exhibited outstanding cytotoxicity of 12.61–62.80 μM. Nevertheless, Ti-3-IO and Ti-5-ICl displayed higher potency in competition with the other complexes in terms of potency and selectivity in HeLa cell lines (HeLa, IC_50_ = 27.17 ± 2.0 μM, selectivity >29) for Ti-3-IO (HeLa, IC_50_ = 24.25 ± 1.7 μM, selectivity >26) for Ti-5-ICl. The cytotoxicity of the complexes on MCF7 and noncancerous HEK cell lines was less, as no differences at all were observed in these cell lines, proving their lower efficacy on these cell lines (Table 6, Fig. 17, ESI Fig. S92 and S93†). We chose the previously suggested IC_50_ values of Ti-3-IO and Ti-5-ICl to proceed with research to conduct additional cytotoxic experiments.

**Table 6:** MTT assay of synthesized Ti(iv) complexes at 48 h of drug exposure and calculated IC_50_ values

S. no.	Code	IC_50_ values in μM μL^−1^		
		HeLa	MCF7	HEK-293
1	Ti-1-IS	61.76 ± 0.1	109.22 ± 0.1	—
2	Ti-2-IN	74.04 ± 0.5	85.04 ± 0.5	>250
3	Ti-3-IO	27.17 ± 1.1	102.88 ± 0.3	—
4	Ti-4-IF	47.28 ± 0.5	109.78 ± 0.2	—
5	Ti-5-ICl	24.25 ± 0.7	65.22 ± 1.2	>250
6	Ti-6-IBr	78.64 ± 0.2	84.67 ± 0.5	—

### 3.14 AO–EB staining

The most important requirement in the creation of novel anticancer drugs is to determine the apoplastic induction caused by the drug concerned. AO–EB assay staining aids in the differentiation of necrotic, apoptotic, and living cells. By means of AO–EB staining, the morphological alterations caused by Ti-1-IS to Ti-6-IBr in HeLa cells were investigated. AO can penetrate intact cell membranes and dye them green; this distinction is caused in early apoptotic as well as normal cells. In contrast, EB can only enter cells that have lost their ability to form membranes, leaving an orange stain behind, identifying them as necrotic or late apoptotic cells.

**Fig. 18:**
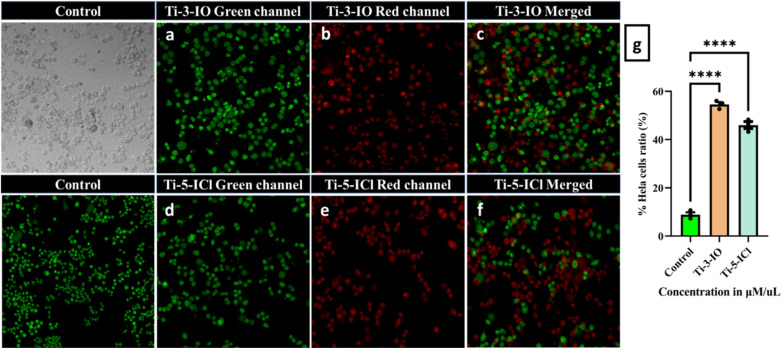
(i) (a–c) AO–EB staining of HeLa cells with Ti-3-IO (27.17 μM μL^−1^, equivalent concentrations) for 48 h; (ii) (d–f) AO–EB staining of HeLa cells Ti-5-IBr (24.25 μM μL^−1^, equivalent concentrations) for 48 h; (iii) (g) % cell population in the microscopic field in the AO–EB staining graph represented as the mean ± s.d.; ns *p* < 0.033.

Fig. 18 depicts the microscopic fluorescence images of HeLa cancer cells with and without complexes. The control in Fig. 18 depicts the living cells as green, denoting intact cell nuclei (unaffected). However, when these malignant cells were exposed to IC_50_ concentrations of Ti-3-IO (27.17 μM) and Ti-5-ICl (24.25 μM), for 48 hours, apoptosis was triggered, and the cells displayed both green and red fluorescence. This suggests that in HeLa cancer cells, morphological alterations, such as nuclear shrinkage, chromatin condensation, and cell membrane disintegration occur, eventually resulting in early and late apoptosis. We deduced from Fig. 18(a–f) that apoptosis was therefore activated by the potent Ti-3-IO and Ti-5-ICl complexes.

### 3.15 Flow cytometry for the detection of cell cycle arrest

**Fig. 19:**
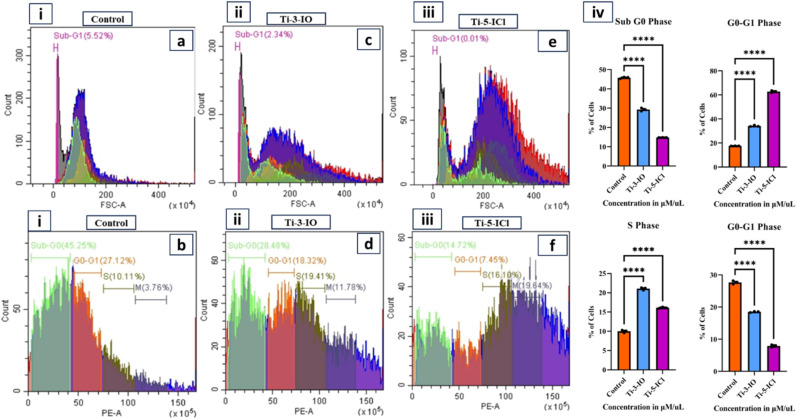
Cell cycle analysis of HeLa cells by PI staining for 48 h; (i) control (a and b); (ii) Ti-3-IO (27.17 μM μL^−1^, equivalent concentrations) (b and c); (iii) Ti-5-IBr (24.25 μM μL^−1^, equivalent concentrations) (c and d); (iv) graphical representation of the different phases of the cell cycle on treatment with Ti-3-IO and Ti-5-IBr; *P* values of >0.12 (ns), 0.033 (*), 0.002 (**), 0.001 (***), 0.001 (****) were considered significant.

Through cell cycle analysis, the mechanism of cancer cell death caused by the complexes was evaluated. Anticancer drugs promote growth-inhibitory action in cancer cells by a variety of mechanisms, one of which is dysfunction in the regulation of the cell cycle.^32^ For example, *cis*-platin inhibits DNA transcription in cells by stopping the cell cycle in the S and G2/M phases. PI staining and flow cytometry were used to examine the ability of the complexes to modify the cell cycle (Fig. 19). The amount of bound DNA in each stage of the cell cycle is measured by flow cytometry, and this amount is directly correlated with the luminescence that PI displays.

IC_50_ concentrations of Ti-3-IO (27.17 μM) and Ti-5-ICl (24.25 μM) were treated with HeLa cell lines. After 48 hours of exposure and workup with PI, the flow cytometer data was obtained for the cell lines. It was observed that the HeLa cell lines displayed 45.25% sub-G0 phase, 27.12% G0/G1 phase, 10.11% S phase and 17.52% M phase under the control conditions. Similar alterations were observed for both Ti-3-IO and Ti-5-ICl, as the cells displayed sub-G0, G0/G1, S, and M phases at 28.48, 18.32, 19.41, and 33.79% for Ti-3-IO and 14.72% sub-G0 phase, 7.45% G0/G1 phase, 16.10% S phase and 61.73% M phase, respectively. The results of the cell cycle analysis are displayed in Fig. 19 and Table 7.

**Table 7:** Tabular representation of phases of the cell cycle analysis on different treatments with Ti-3-IO and T-5-ICl

Sl. no.	Condition	Sub-G0 phase	G0/G1 phase	S Phase	M Phase
1	*Cis*-platin	45.25%	27.12%	10.11%	17.52%
2	Ti-3-IO (27.17 μM)	28.48%	18.32%	19.41%	33.79%
3	Ti-5-ICl (24.25 μM)	14.72%	7.45%	16.10%	61.73%

### 3.16 Generation of reactive oxygen species (ROS)

ROS have been proposed as potential mediators of oxidative stress-induced apoptosis caused by titanium(iv) complexes, and the electrophoresis experiment mentioned above supports this theory. The content of ROS in HeLa cells was measured using a DCFH-DA fluorescent probe (Fig. 4) after they were treated with IC_50_ doses of Ti-3-IO (27.17 μM) and Ti-5-ICl (24.25 μM) for 48 hours to determine whether the ROS concentration influences cell apoptosis. Both Ti(iv) complexes promote ROS production in HeLa cell lines, as evidenced by the increased fluorescence seen in the Ti-3-IO and Ti-5-ICl treated groups compared to the control.

**Fig. 20:**
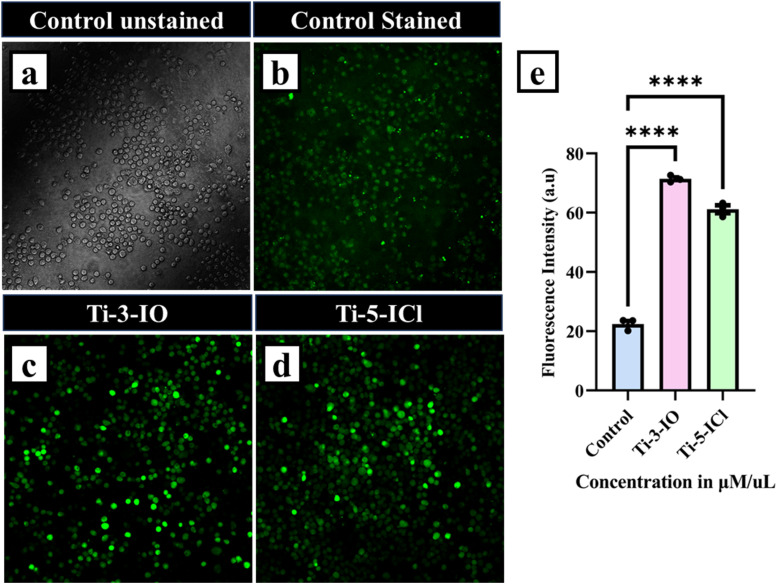
Fluorescence images of ROS levels in HeLa cells treated with (a) unstained control, (b) control stained and treated with IC_50_ equivalent concentrations of (c) Ti-3-IO and (d) Ti-5-ICl for 48 h. (e) Graphical data representing the ROS levels in complex-treated HeLa cells. All quantified values are expressed as mean ± SD (*n* = 3). Statistically significant values at ns < 0.033.

Apoptosis or necrosis of cells would result from the destruction of macromolecules like proteins and deoxyribonucleic acid caused by an increase in ROS. Furthermore, the ratio of intracellular ROS was Ti-5-ICl > Ti-3-IO. This indicates that Ti(iv) complexes do, in fact, produce more ROS and induce apoptosis when they come into contact with cancer cells. Additionally, employing a plate reader test, the ROS has been quantified for each compound against HeLa (Fig. 20). The fluorescence intensity of compound Ti-5-ICl was found to be lower at 62.5 au than that of Ti-3-IO (75.6 au) (Fig. 20(a–e)). Since ROS-mediated DNA damage is the primary cause of cell death in A549 cells, the named complexes might enhance intracellular oxidative stress and induce apoptosis.

## 4 Conclusion

The creation herein of titanium(iv) complexes incorporating ONO homoleptic Schiff base derivatives was the main goal of the current investigation. NMR, HRMS, FTIR, and UV-vis analytical tools were used to characterize these complexes. UV-vis and fluorescence spectrophotometric techniques were used to perform the photophysical characteristic and DNA/BSA interaction experiments. In order to understand the molecular insights, parameters and docking scores followed by binding poses with BSA/DNA, DFT and molecular docking experiments were included.

Further, complexes Ti-1-IS to Ti-6-IBr exhibited profound cytotoxicity towards cancerous cells compared to free ligands and *cis*-platin. More specifically, Ti-3-IO and Ti-5-ICl displayed enhanced activity against HeLa cells with lower IC_50_ values of 27.17 μM and 24.25 μM, respectively. AO–EB fluorescent staining and flow cytometry analysis revealed that Ti-3-IO and Ti-5-ICl induce cancer cell death in ‘HeLa’ by an apoptosis mechanism. Furthermore, cell cycle analysis indicates that the complexes arrest HeLa cell cycle progression in the M phase. Whereas, from the DCFH-DA assay, it was evident that both complexes exhibited notable ROS properties. Overall, complexes Ti-3-IO and Ti-5-ICl presented in the present communication displayed considerably good anticancer activity even at minimal concentration. Thus, the titanium(iv) complexes described herein might serve as the epicenter for the development of an upcoming class of anticancer medications.

## Author contributions

Shivabasayya V Salimath: methodology, investigation, data curation, writing – original draft; Kavita B Hiremath: investigation and data curation; Mahabarathi Subramaniyan: methodology, validation; Arjita Ghosh: methodology, validation; Evangeline Lawrence: methodology, validation; Murugesh Shivashankar: formal analysis; Anbalagan Moorthy: resources, writing – review & editing; Madhvesh Pathak: conceptualization, resources, writing – review & editing, supervision.

## Conflicts of interest

The authors declare that there is no conflict of interest regarding this publication.

## Supplementary Material

RA-015-D5RA03821A-s001

## Data Availability

Authors declare that the data supporting the findings of this study are available within the paper and in its ESI files.†
