# Supplementary material for: Homoleptic complexes of titanium(iv) fused with O^N^O Schiff base derivatives: design, BSA–DNA interaction, molecular docking, DFT and cytotoxicity
Source: RSC Adv. 2025 Jul 16;15(31):25075–102. doi: 10.1039/d5ra03821a (PMC12264794; doi:10.1039/d5ra03821a)
Supplement: RA-015-D5RA03821A-s001 [file RA-015-D5RA03821A-s001.pdf]

Supplementary Information (SI) for RSC Advances.
This journal is © The Royal Society of Chemistry 2025

Supporting Information

Homoleptic complexes of titanium(IV) fused with O^N^O Schiff Base derivatives: design, BSA-DNA interaction, molecular docking, DFT and cytotoxicity

Shivabasayya V Salimath[a], Kavita B Hiremath[a], Mahabarathi Subramaniyan[a], Arjita Ghosh[b], Evangeline Lawrence[b], Anbalagan Moorthy[b], Murugesh Shivashankar[a] and Madhvesh Pathak[a]*

[a]*Department of Chemistry, School of Advanced Sciences, Vellore Institute of Technology (VIT), Vellore, Tamil Nadu, India*

[b]*Department of Integrative Biology, School of Bioscience and Technology (SBST), Vellore Institute of Technology (VIT), Vellore, Tamil Nadu, India*

**Email: madhveshpathak@vit.ac.in* *ORCID iD: *https://orcid.org/0000-0002-1567-6519*

Figures and tables	Title	Page.No
Fig.S1-S4	NMR, FTIR and ESI-MS data of Ligand [IS]	3-4
Fig.S5-S8	NMR, FTIR and ESI-MS data of Ligand [IN]	5-6
Fig.S9-S12	NMR, FTIR and ESI-MS data of Ligand [IO]	7-8
Fig.S13-S16	NMR, FTIR and ESI-MS data of Ligand [IF]	9-10
Fig.S17-S20	NMR, FTIR and ESI-MS data of Ligand [ICl]	11-12
Fig.S21-S24	NMR, FTIR and ESI-MS data of Ligand [IBr]	13-14
Fig.S25-S29	NMR, FTIR, ESI-MS and HPLC data of Complex [Ti-1-IS]	14-17
Fig.S30-S34	NMR, FTIR, ESI-MS and HPLC data of Complex [Ti-2-IN]	17-19
Fig.S35-S39	NMR, FTIR, ESI-MS and HPLC data of Complex [Ti-3-IO]	20-22
Fig.S40-S45	NMR, FTIR, ESI-MS and HPLC data of Complex [Ti-4-IF]	22-25
Fig.S46-S50	NMR, FTIR, ESI-MS and HPLC data of Complex [Ti-5-ICl]	25-27
Fig.S51-S55	NMR, FTIR, ESI-MS and HPLC data of Complex [Ti-6-IBr]	28-30
Fig.S56-S57	UV-Vis and Fluorescence spectra of Ti(IV) complexes in DMSO: H_2O (1:9)	31
Fig.S58-S59	Stability of complexes in 1:9 DMSO: H_2O and GSH medium	32-33
Fig.S60	UV-visible spectra of Ti(IV) complexes for Lipophilicity study of complexes in octanol: water	34
Fig.S61	UV-Visible spectra of DNA Binding studies of Ti(IV) complexes (a)Ti-1-IS, (b) Ti-2-IN, (c) Ti-3-IO, (d) Ti-4-IF, (e) Ti-5-Cl and (f) Ti-6-IBr	35
Fig.S62	Linear plots of DNA UV-binding studies of Ti(IV) complexes	36
Fig.S63	Fluorescence quenching spectra of DNA with increasing concentration of Ti(IV) complexes	37
Fig.S64	Stern-Volmer plots of I0/I vs. Ti(IV) complexes	38
Fig.S64	Scatchard plots of log([I_0-I]/I) vs. log [Ti(IV) complexes]	38

Fig.S66	Viscosity studies of Complexes	39
Fig.S67	Cyclic voltammetry DNA binding Studies of Ti(IV) complexes	40
Table S1	Oxidation and reduction peaks obtained from Cyclic Voltammograms of Ti(IV) complexes with CT-DNA(0–50 µM)	41
Fig.S68	Fluorescence quenching spectra of BSA with increasing concentration of Ti(IV) complexes	42
Fig.S69	Stern-Volmer plots of I0/I vs. Ti(IV) complexes	43
Fig.S70	Scatchard plots of log($[I_0$-I]/I) vs. log [Ti(IV) complexes]	43
Fig.S71	Synchronous spectra of BSA with increasing concentration of Ti(IV) complexes at Δλ=15 nm,	44
Fig.S72	Stern-Volmer plots of I_0/I vs. complex of Synchronous spectra of BSA with increasing concentration of Ti(IV) complexes at Δλ=15 nm	45
Fig.S73	Synchronous spectra of BSA with increasing concentration of Ti(IV) complexes at Δλ=60 nm,	45-46
Fig.S74	Stern-Volmer plots of I_0/I vs. complex of Synchronous spectra of BSA with increasing concentration of Ti(IV) complexes at Δλ=15 nm	46
Table S2	Stern-Volmer plots of I_0/I vs. complex of Synchronous spectra of BSA with increasing concentration of Ru(II) complexes at Δλ=15 nm and 60nm	46
Fig.S75	Site marker fluorescence quenching studies of BSA+Ibuprofin with an increase in the concentration of Ti(IV) complexes	47
Fig.S76	Scatchard plot of log($[I_0$-I]/I) vs log [complex]of site marker fluorescence quenching studies of BSA+Ibuprofin	48
Fig.S77	Site marker fluorescence quenching studies of BSA+Warfarin with an increase in the concentration of Ti(IV) complexes	49
Fig.S78	Scatchard plot of log($[I_0$-I]/I) vs log [complex]of site marker fluorescence quenching studies of BSA+Warfarin	50
Table S3	The comparison of binding constants of the complexes Ti(IV) with BSA before and after the addition of site probe at 298 K	50
Fig.S79-S84	Molecular docking of DNA with Ti(IV) complexes	51-53
Table S4	Docking scores and binding sites of DNA with Ti(IV) complexes	54
Table S5	Docking scores and binding sites of BSA with Ti(IV) complexes	54
Fig.S85-S90	Molecular docking of BSA with Ti(IV) complexes	55-57
Table S6	Bond length (Å) of Ti(IV) complexes	58
Table S7	Comparison of experimental and theoretical excitation spectral details	58
Fig.S91	DPPH assay of Ti(IV) complexes	59
Fig.S92-S93	MTT assay of Ti(IV) complexes on HeLa and MCF7 cell line	60-61
	References	62

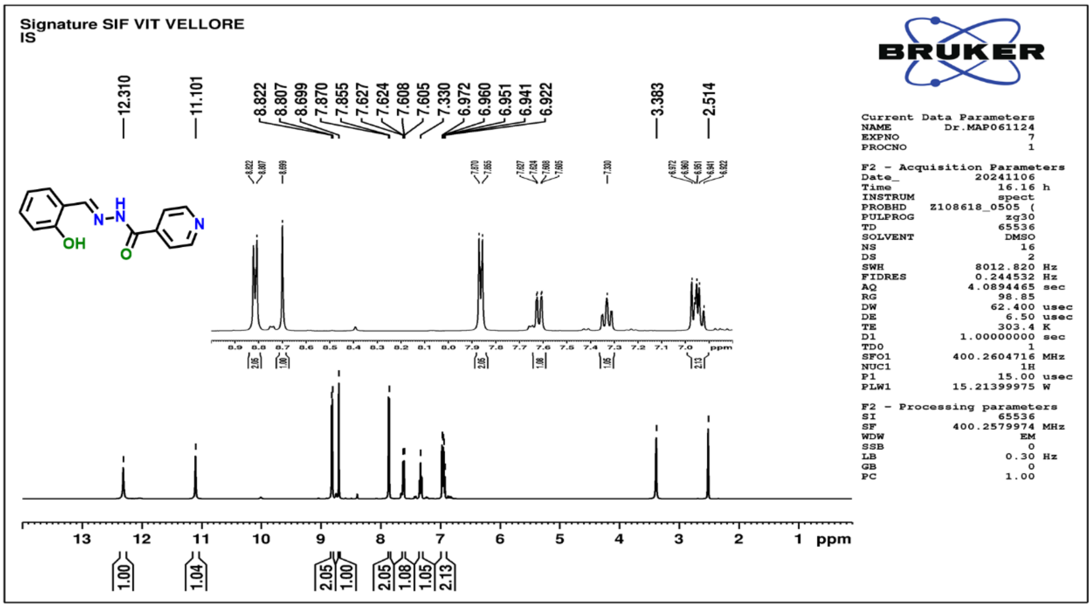

Figure.S1. ^{1}H NMR spectrum of ligand IS (400 MHz, DMSO-d_6)

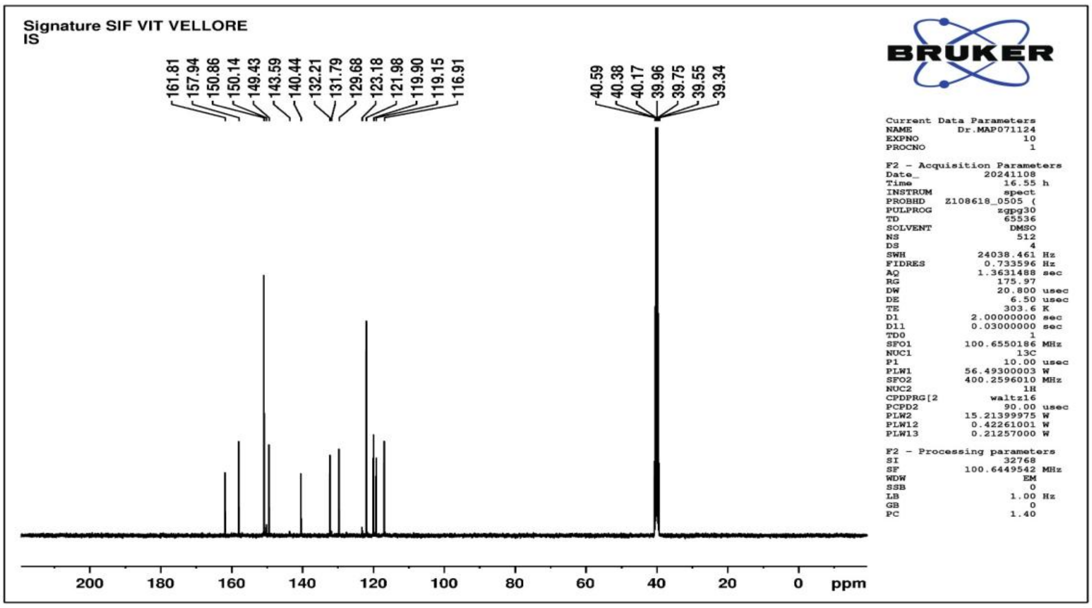

Figure.S2. ^{13}C NMR spectrum of ligand IS (400 MHz, DMSO-d_6)

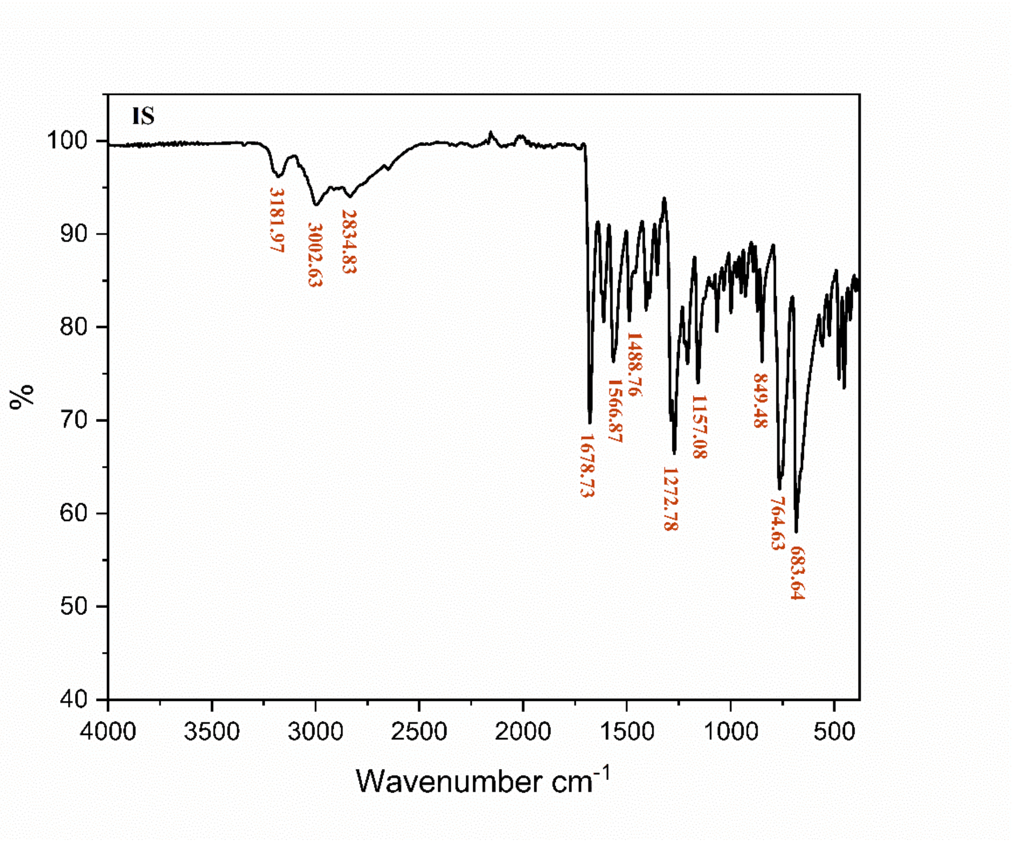

Figure.S3. FTIR spectrum of ligand IS (400 MHz, DMSO-d$_6$)

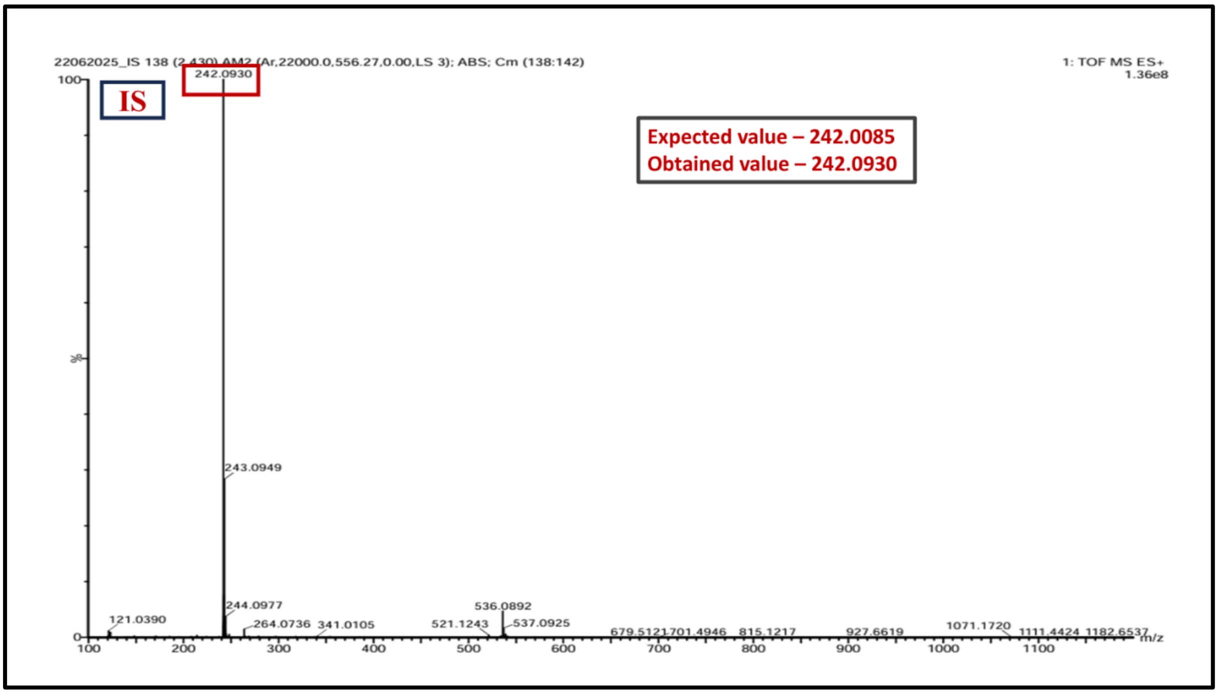

Figure.S4. ESI-MS spectrum of ligand IS

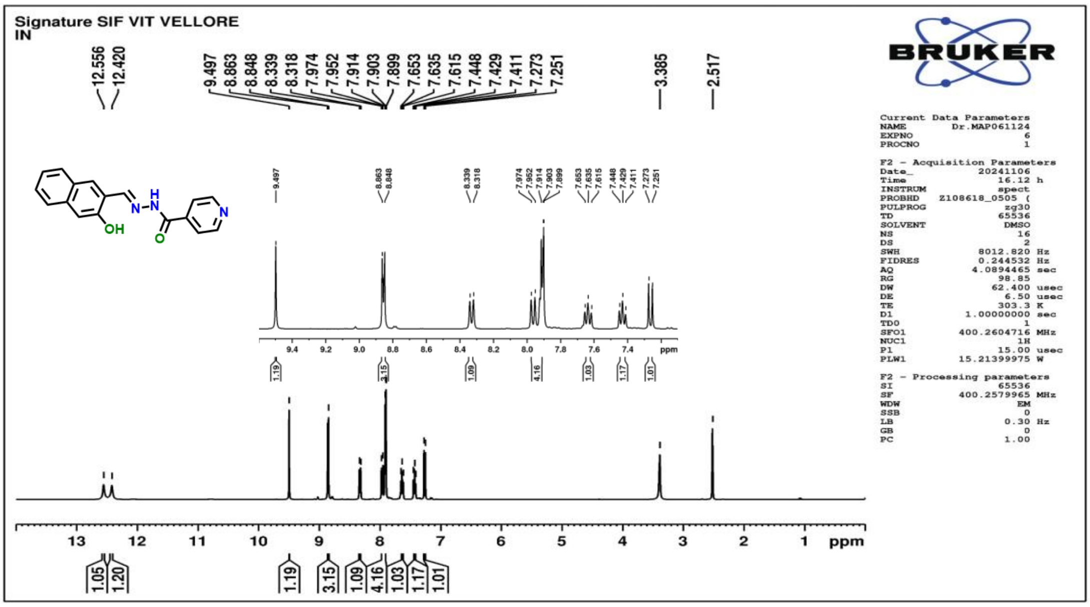

Figure.S5. ^{1}H NMR spectrum of ligand IN (400 MHz, DMSO-d$_6$)

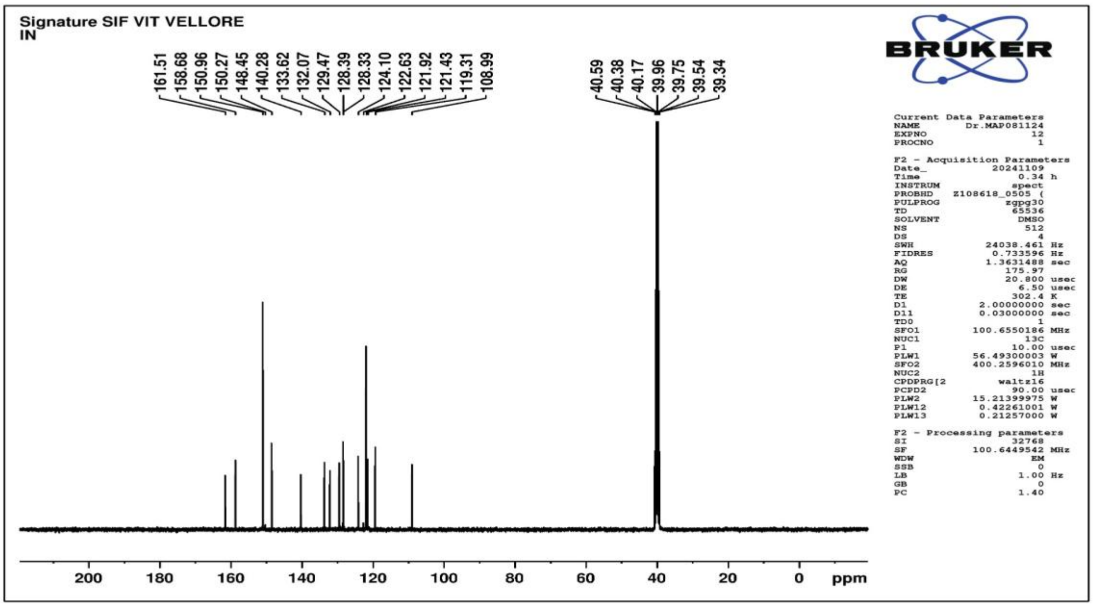

Figure.S6. ^{13}C NMR spectrum of ligand IN (400 MHz, DMSO-d$_6$)

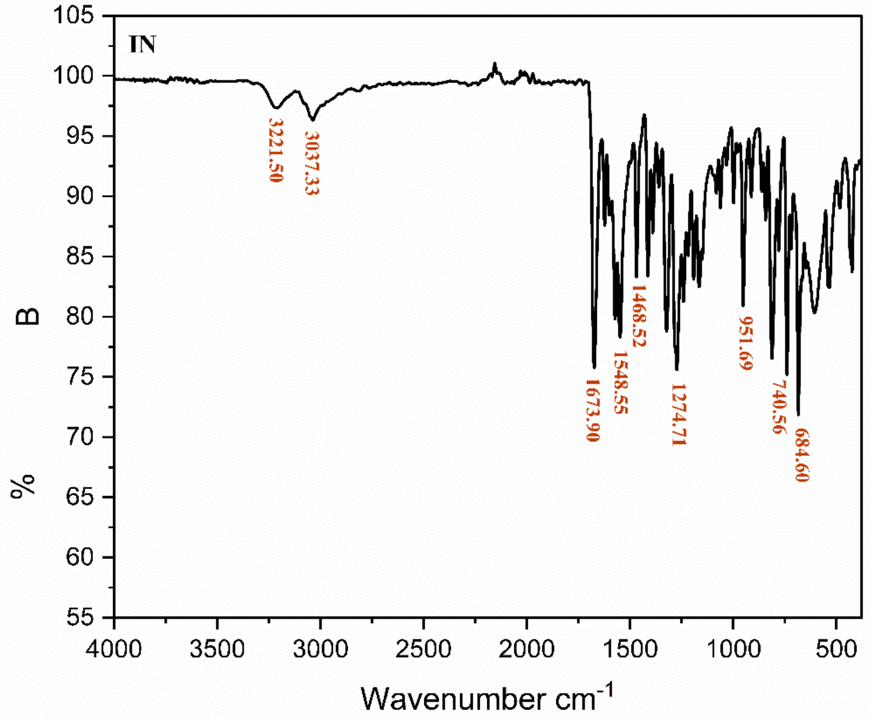

Figure.S7. FTIR spectrum of ligand IN (400 MHz, DMSO-d_6)

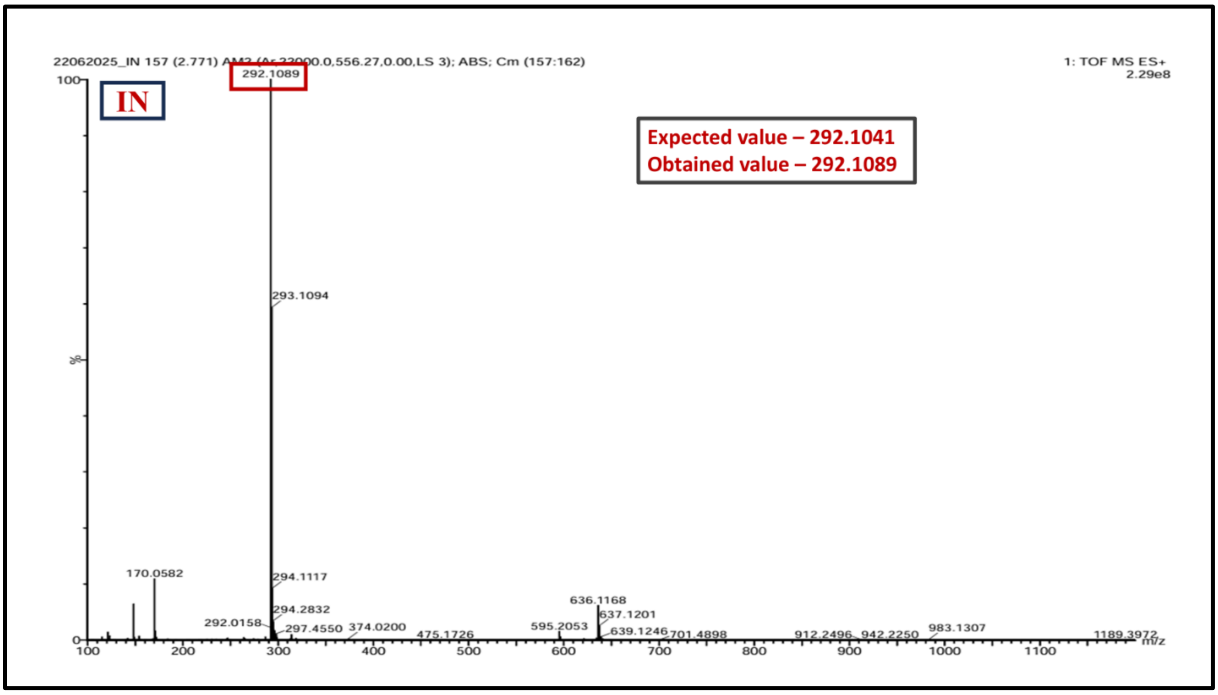

Figure.S8. ESI-MS spectrum of ligand IN

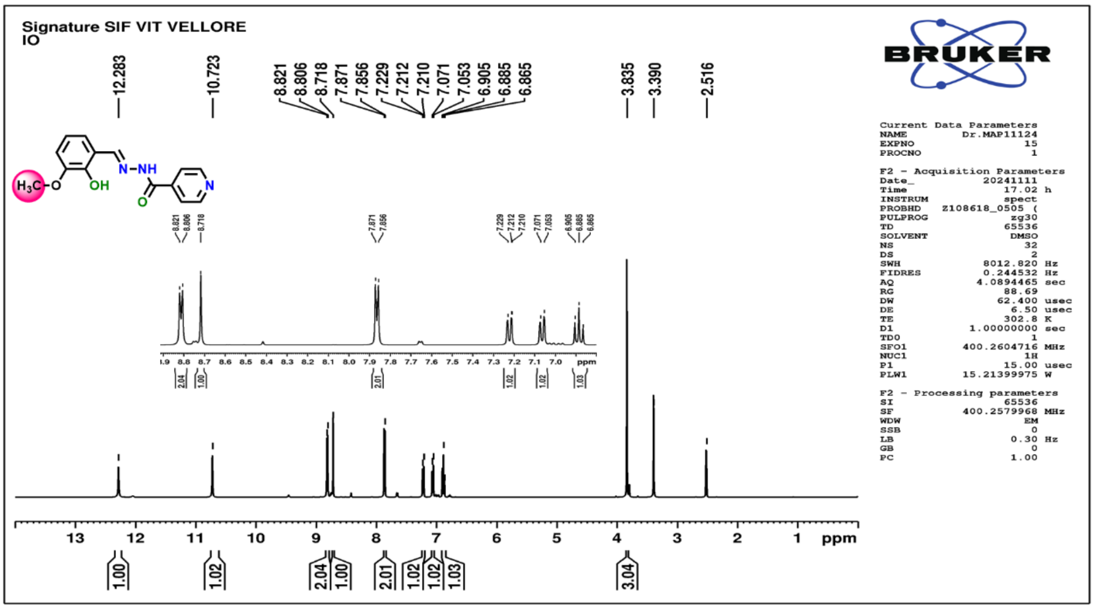

Figure.S9.^{1}H NMR spectrum of ligand IO (400 MHz, DMSO-d_6)

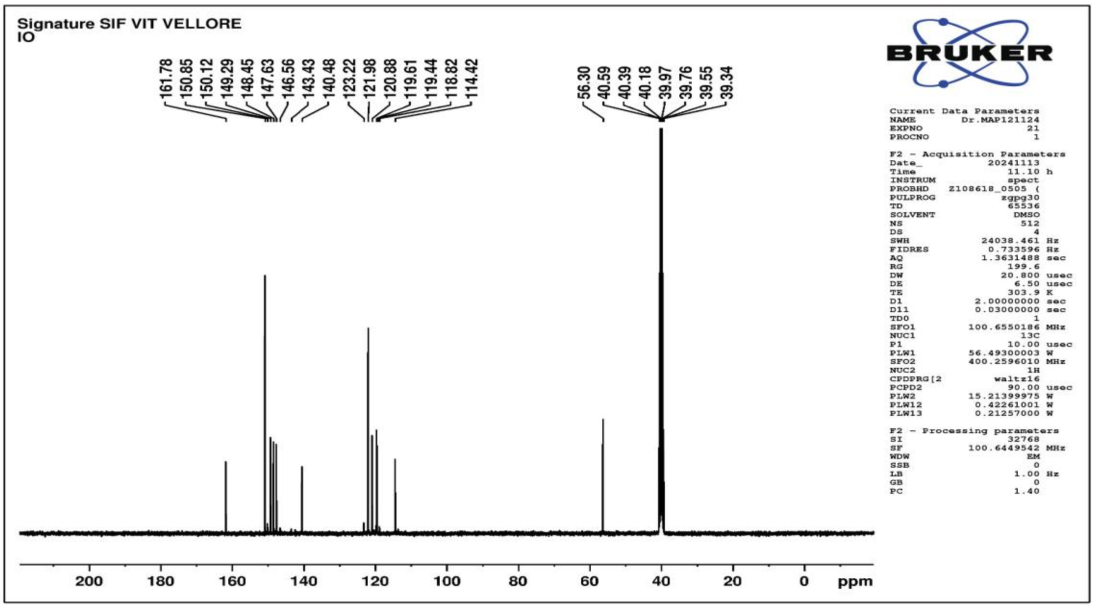

Figure.S10. ^{13}C NMR spectrum of ligand IO (400 MHz, DMSO-d_6)

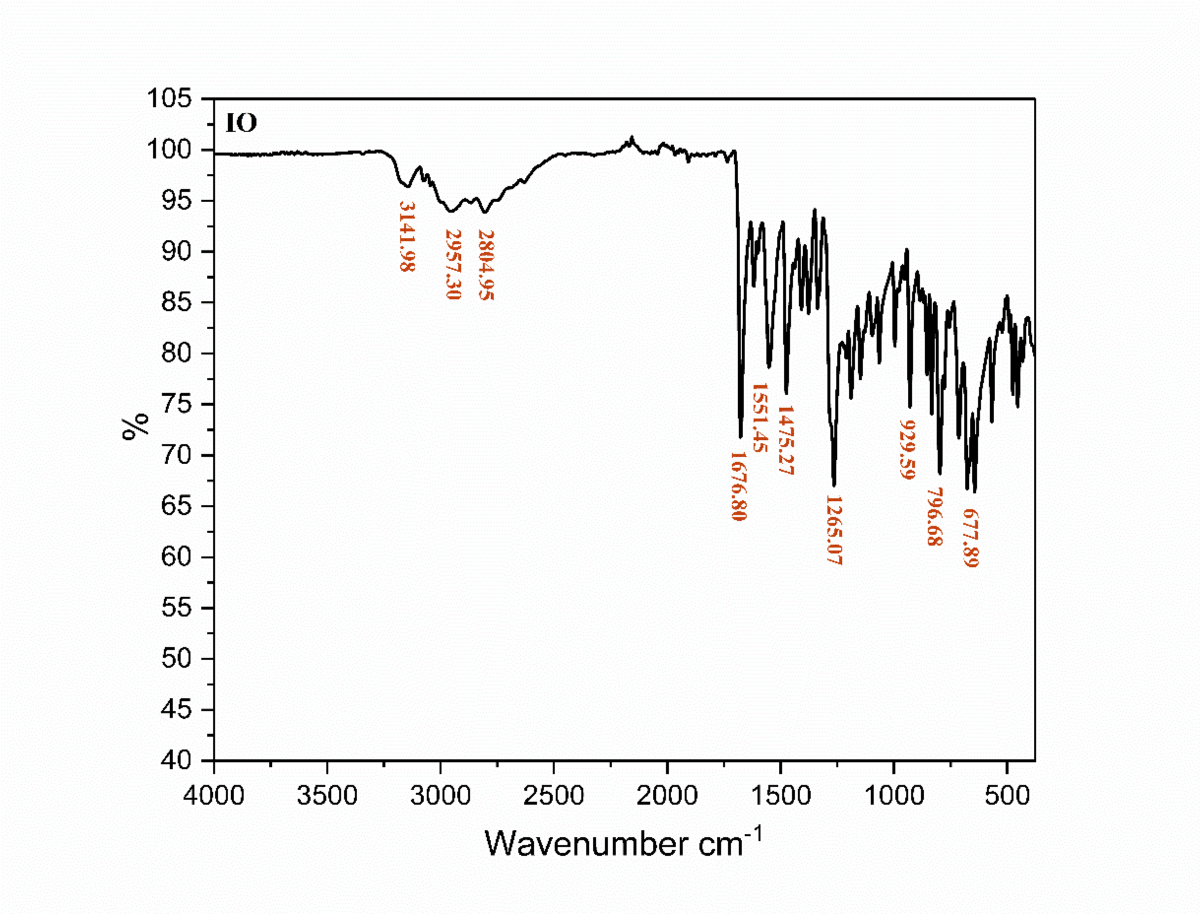

Figure.S11. FTIR spectrum of ligand IO

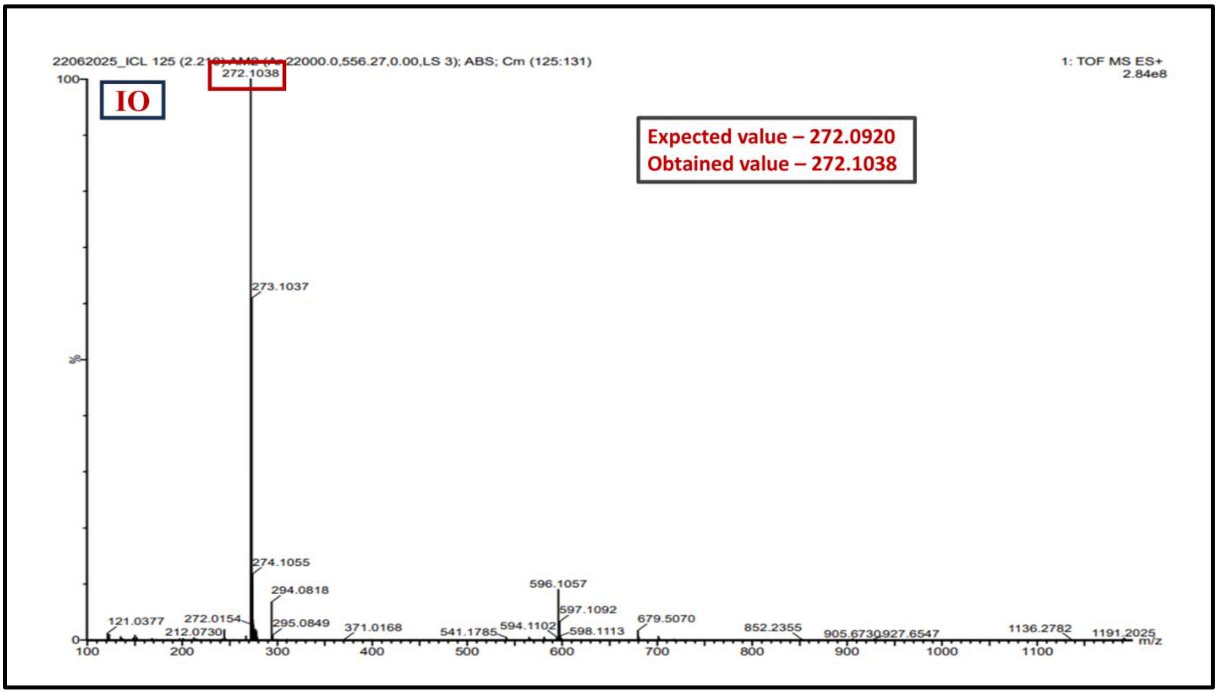

Figure.S12. ESI-MS spectrum of ligand IO

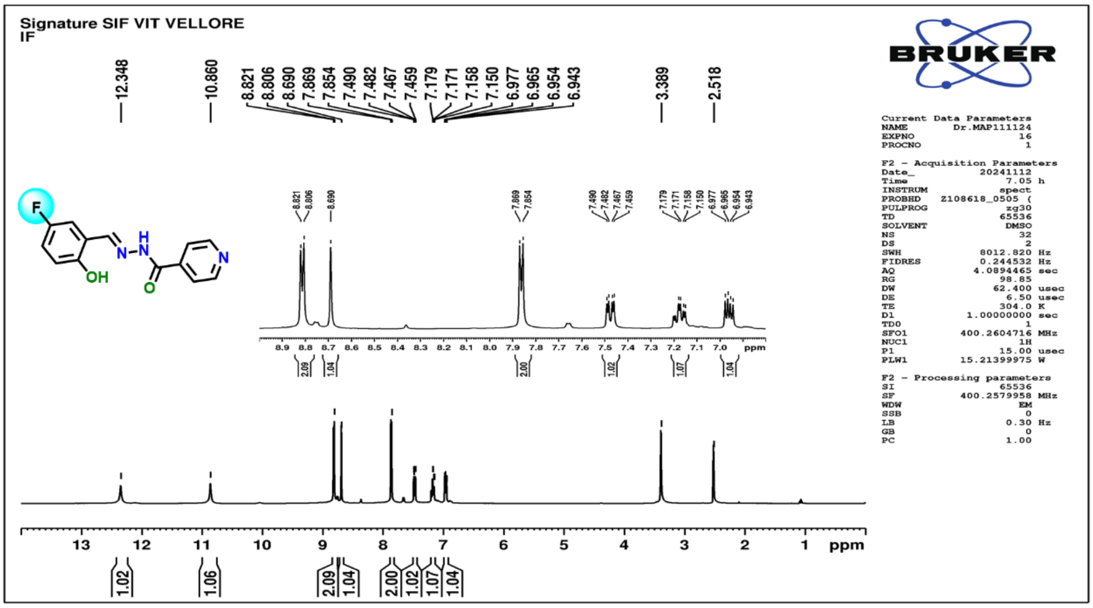

Figure.S13. ^{1}H NMR spectrum of ligand IF(400 MHz, DMSO-d_6)

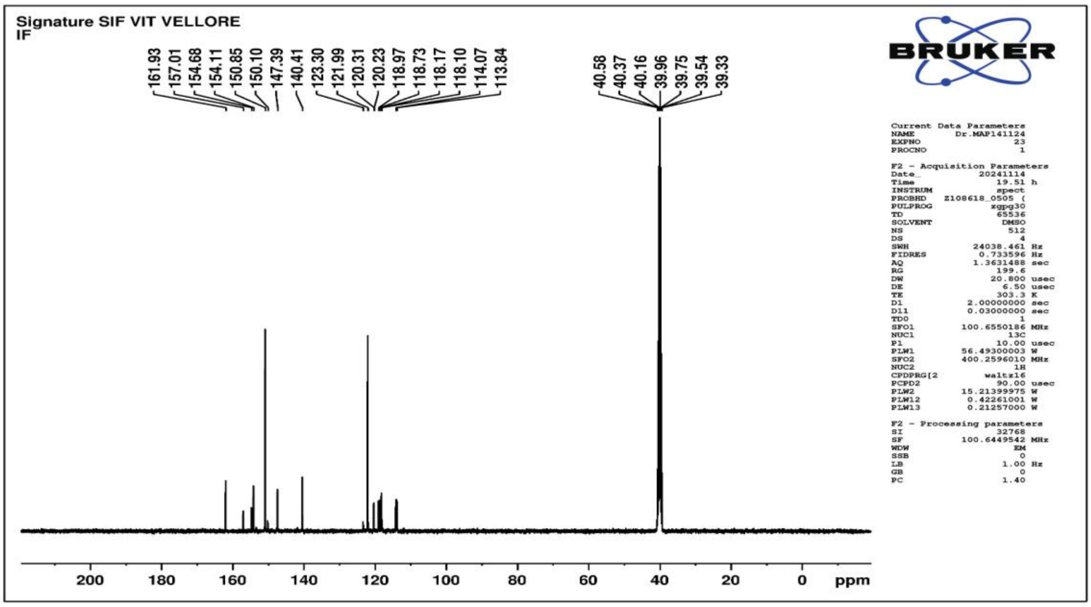

Figure.S14. ^{13}C NMR spectrum of ligand IF(400 MHz, DMSO-d_6)

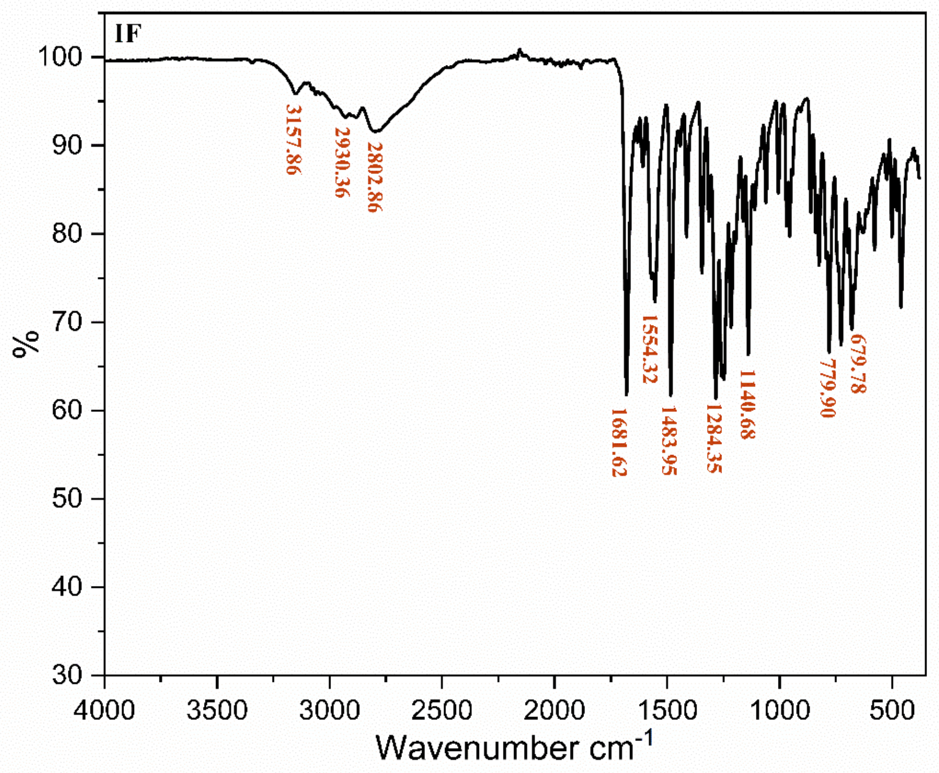

Figure.S15. FTIR spectrum of ligand IF (400 MHz, DMSO-d$_6$)

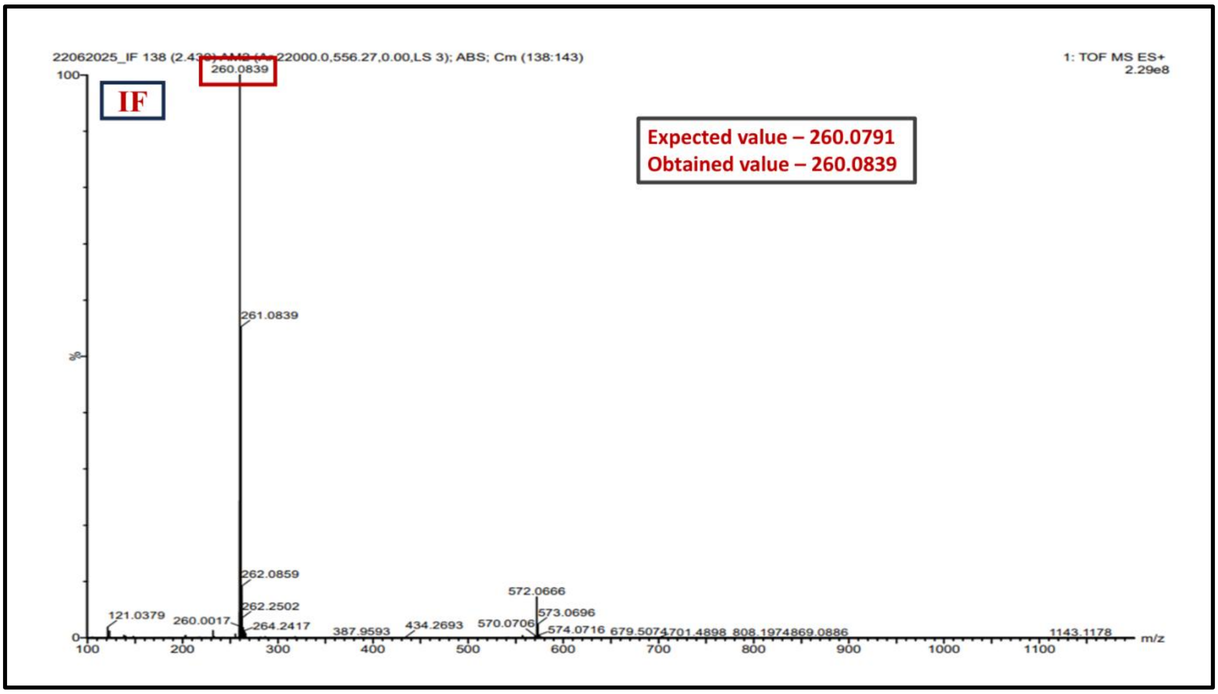

Figure.S16. ESI-MS spectrum of ligand IF

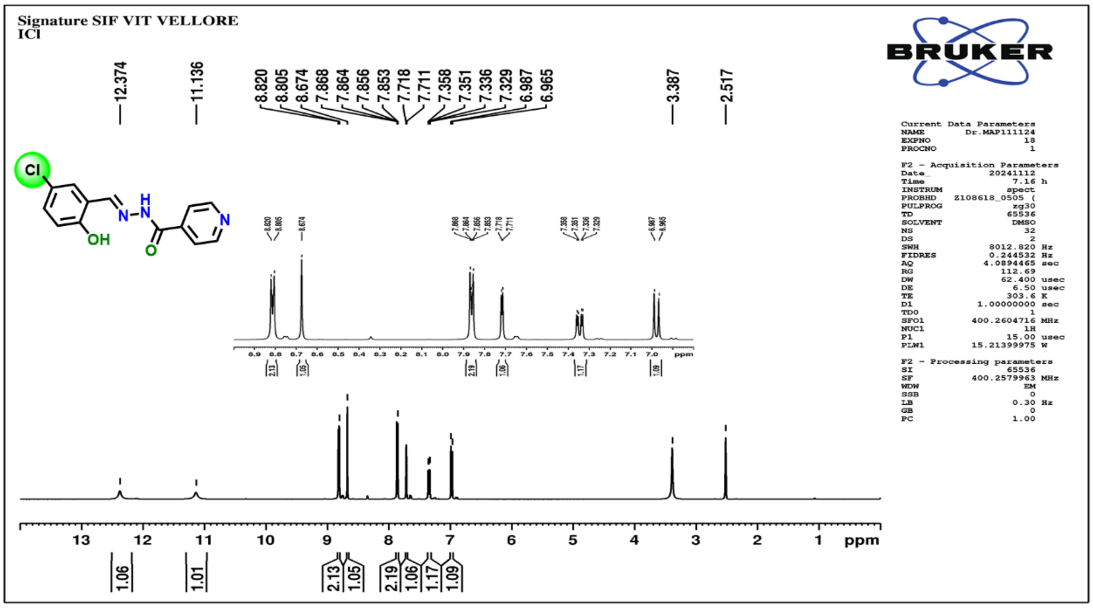

Figure.S17. ^{1}**H NMR spectrum of ligand ICl (400 MHz, DMSO-d$_6$)**

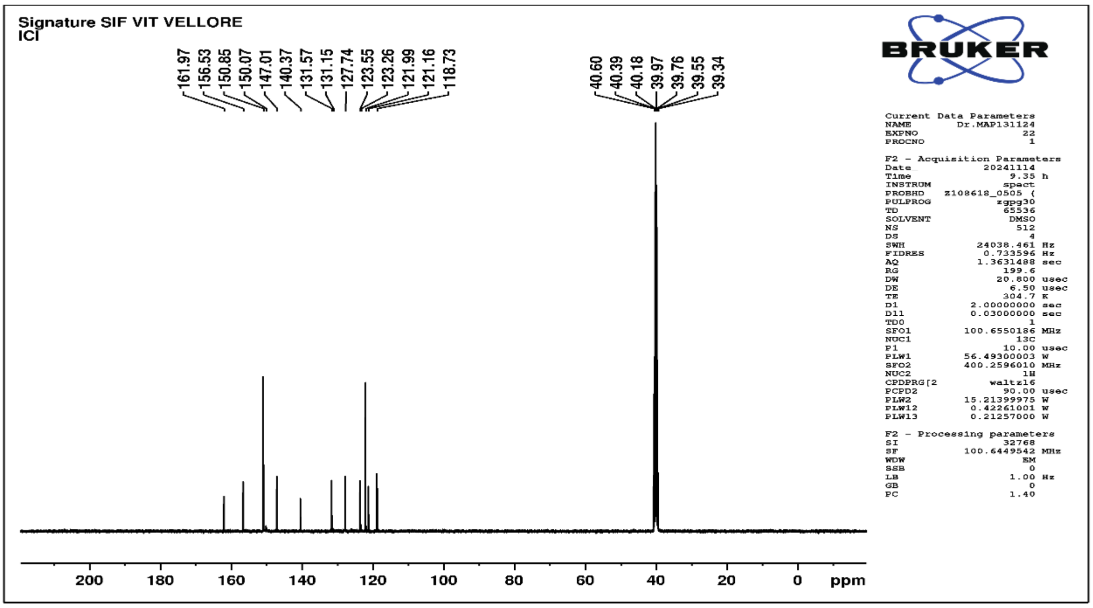

Figure.S18. ^{13}**C NMR spectrum of ligand ICl (400 MHz, DMSO-d$_6$)**

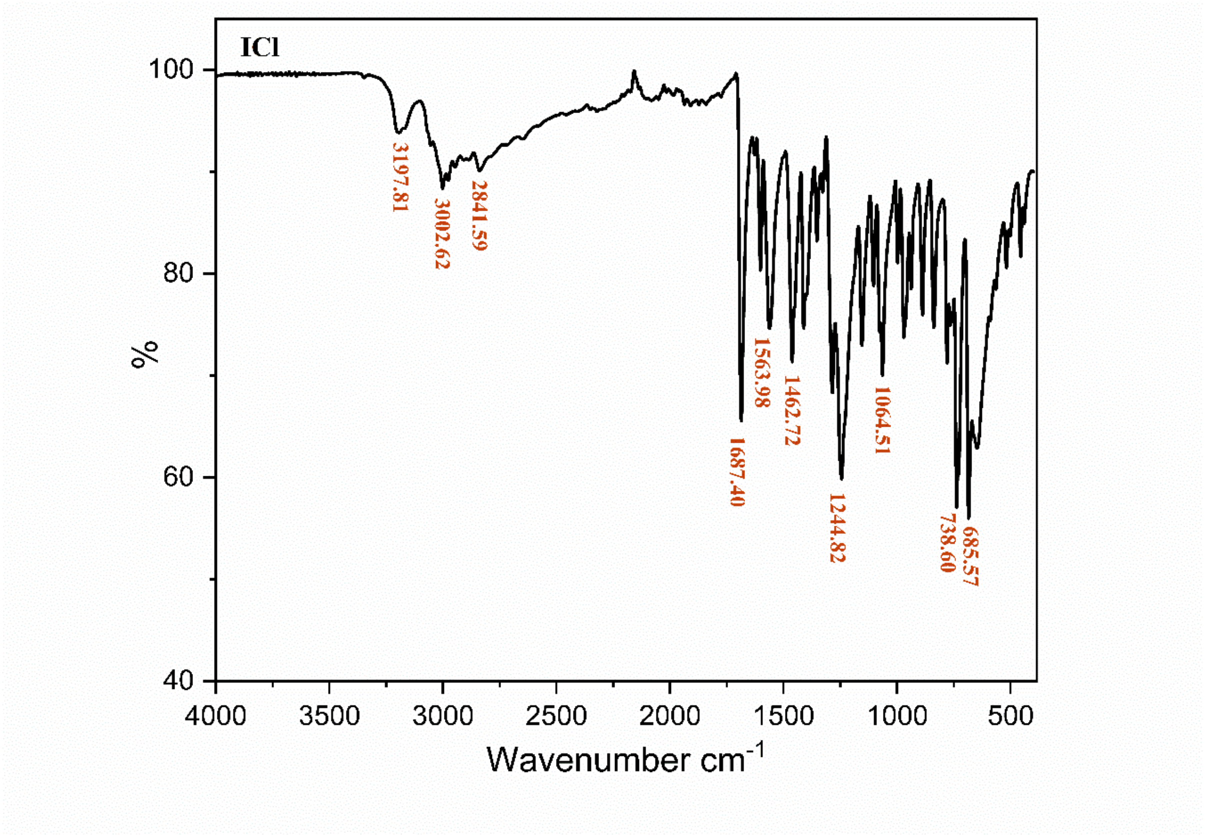

Figure.S19. FTIR spectrum of ligand ICl

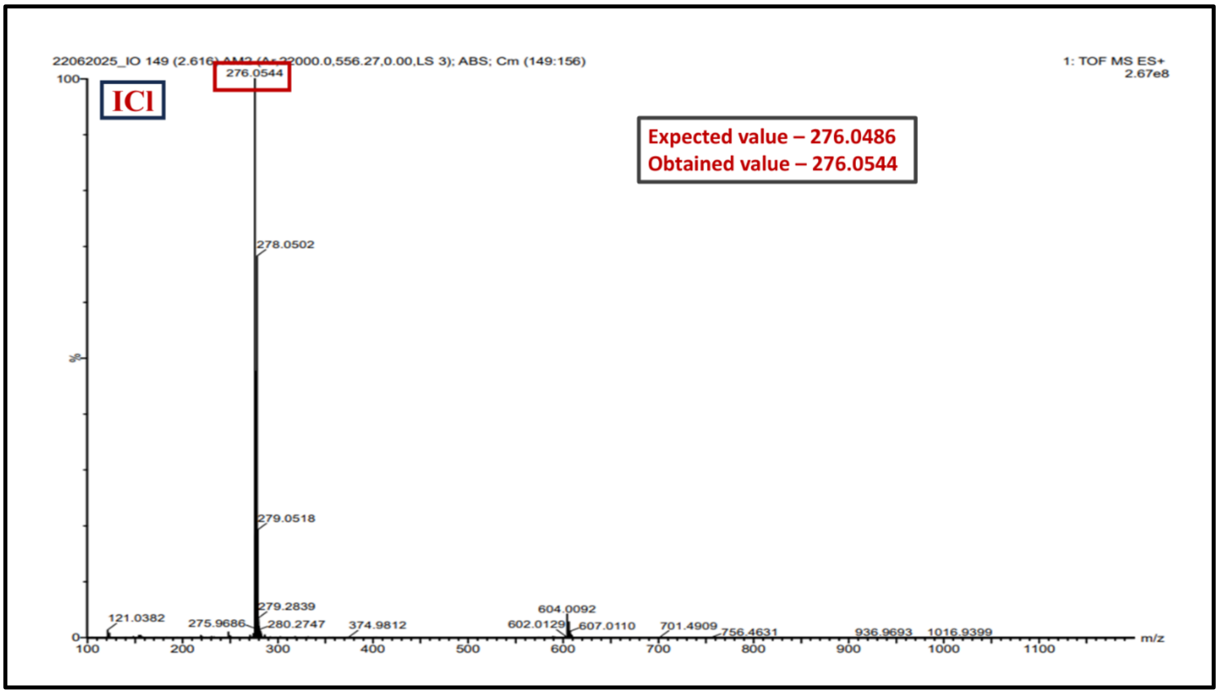

Figure.S20. ESI-MS spectrum of ligand ICl

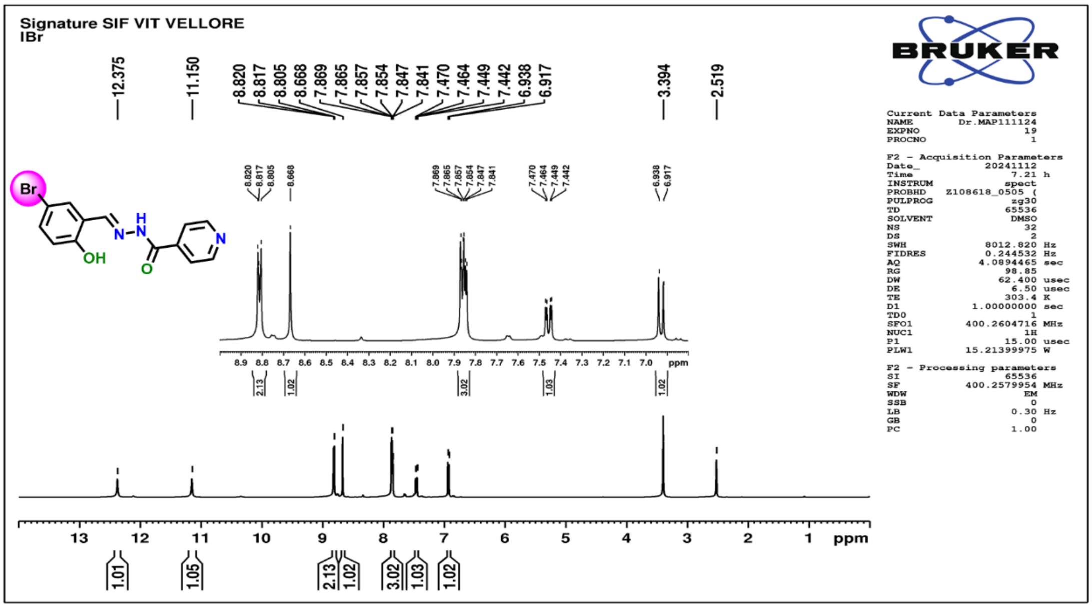

Figure.S21. ^{1}H NMR spectrum of ligand IBr (400 MHz, DMSO-d$_6$)

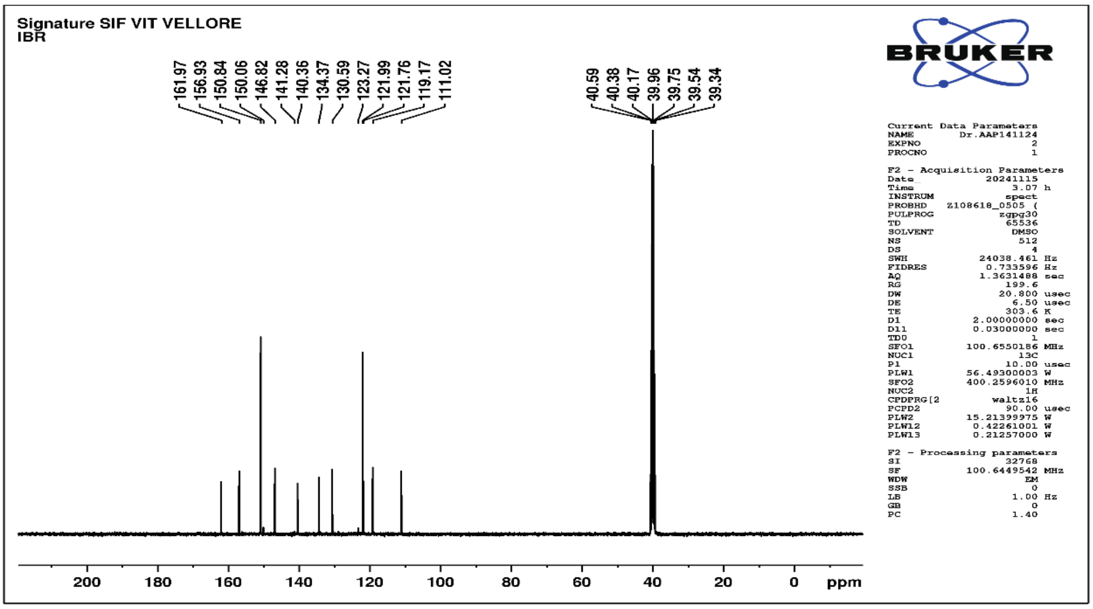

Figure.S22. ^{13}C NMR spectrum of ligand IBr (400 MHz, DMSO-d$_6$)

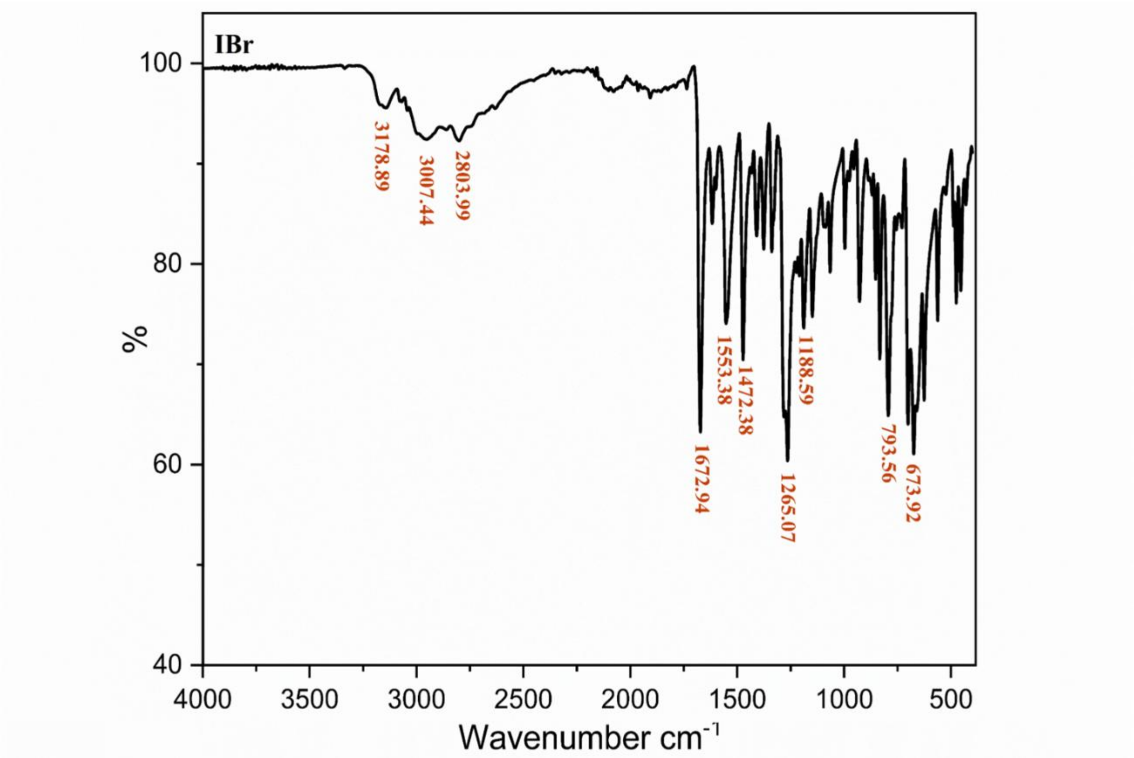

Figure.S23. FTIR spectrum of ligand IBr

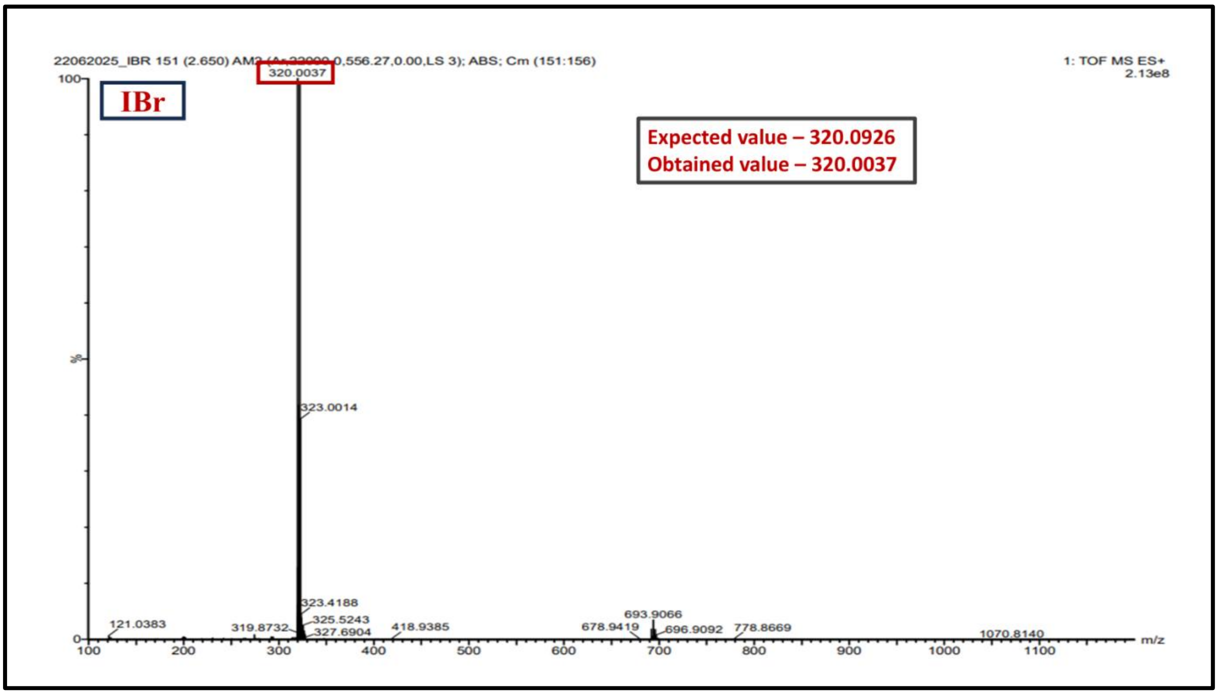

Figure.S24. ESI-MS spectrum of ligand IF

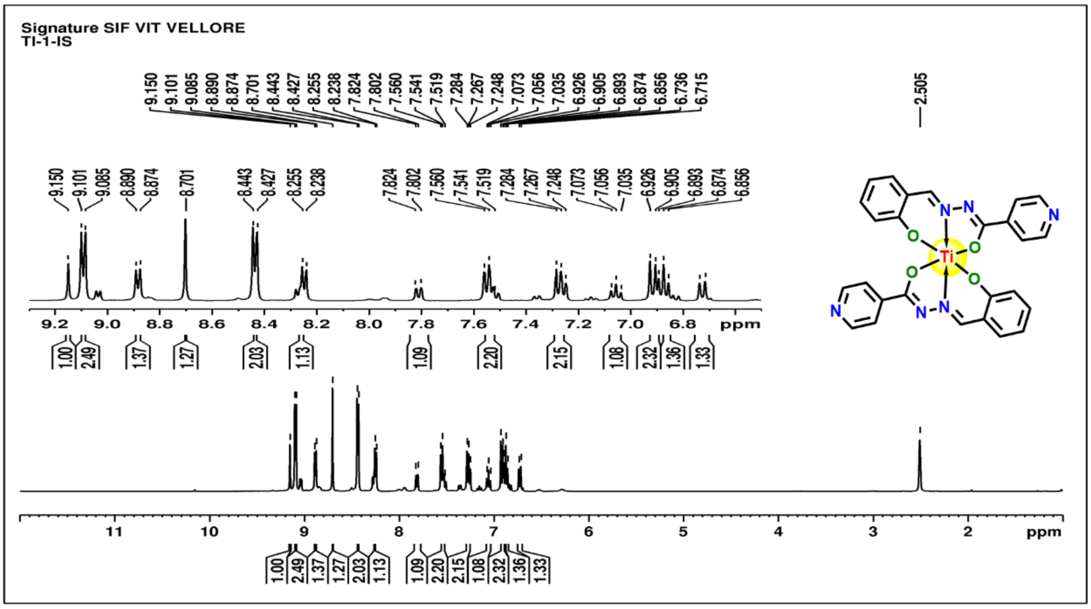

Figure.S25. ^{1}H NMR spectrum of complex Ti-1-IS (400 MHz, DMSO-d_6)

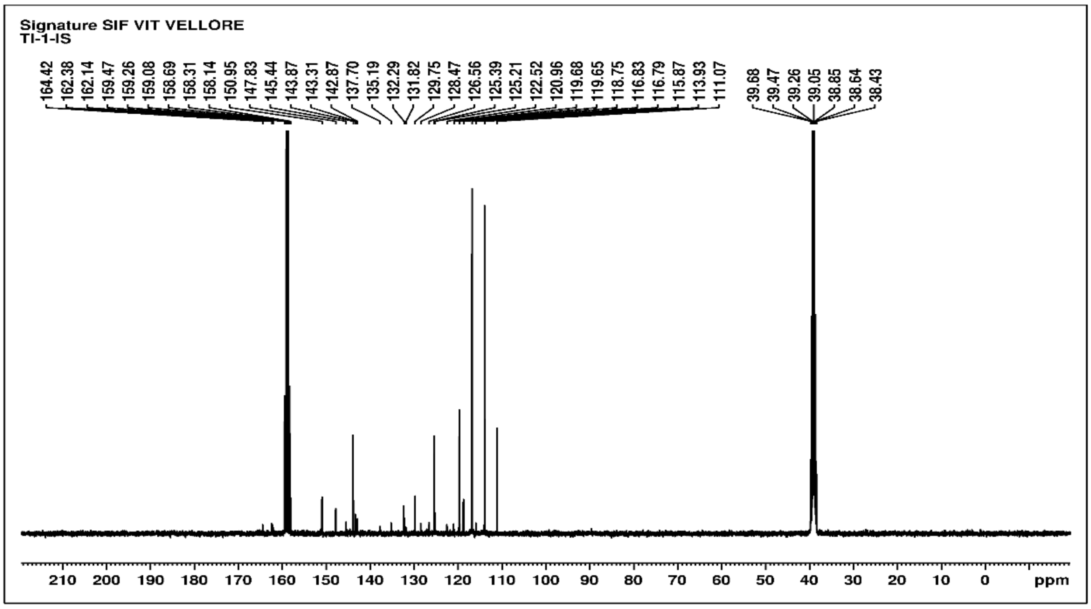

Figure.S26. ^{13}C NMR spectrum of complex Ti-1-IS (400 MHz, DMSO-d_6)

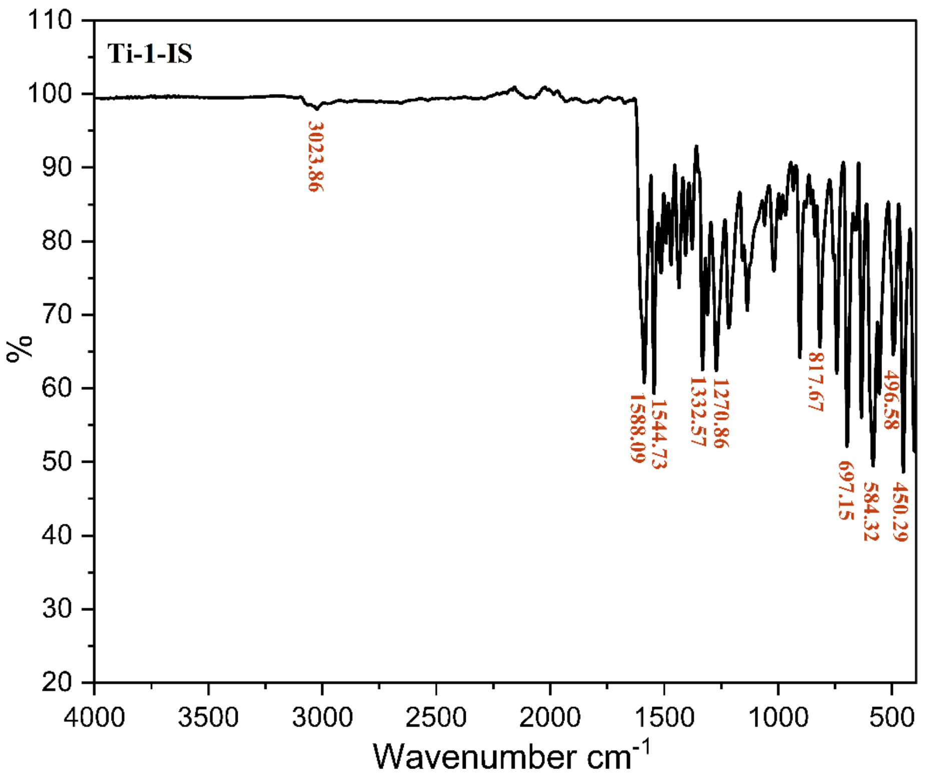

Figure.S27. FTIR spectrum of complex Ti-1-IS (400 MHz, DMSO-d_6)

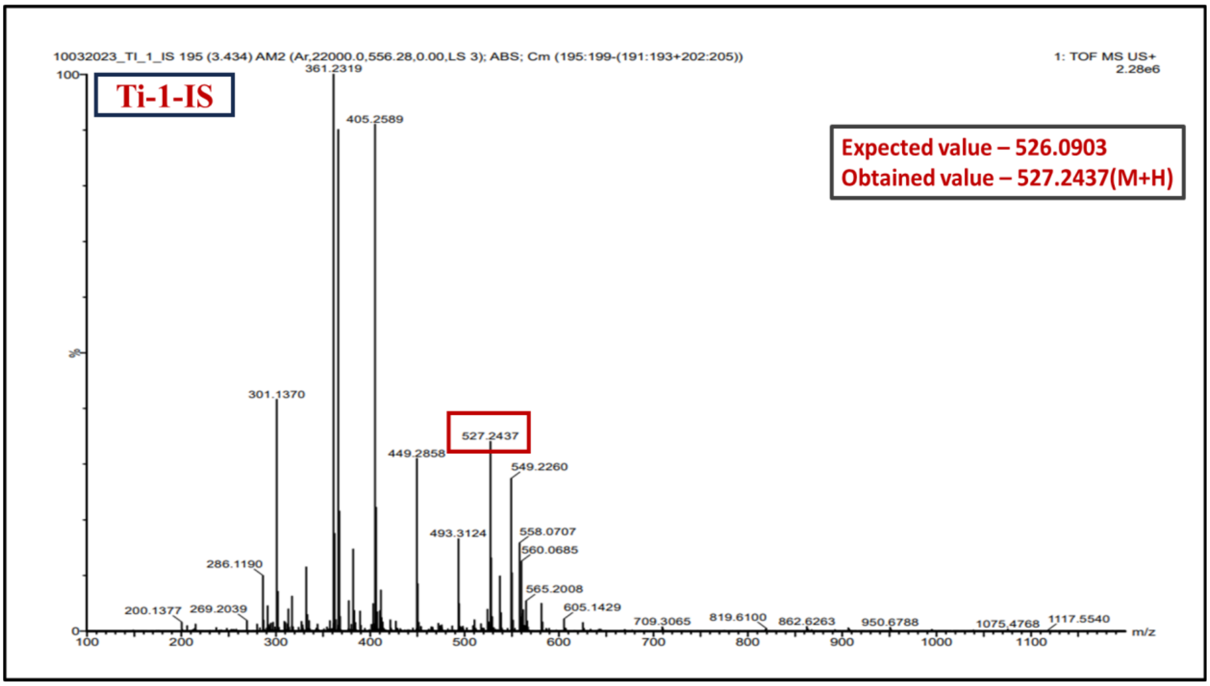

Figure.S28. ESI-MS spectrum of complex Ti-1-IS

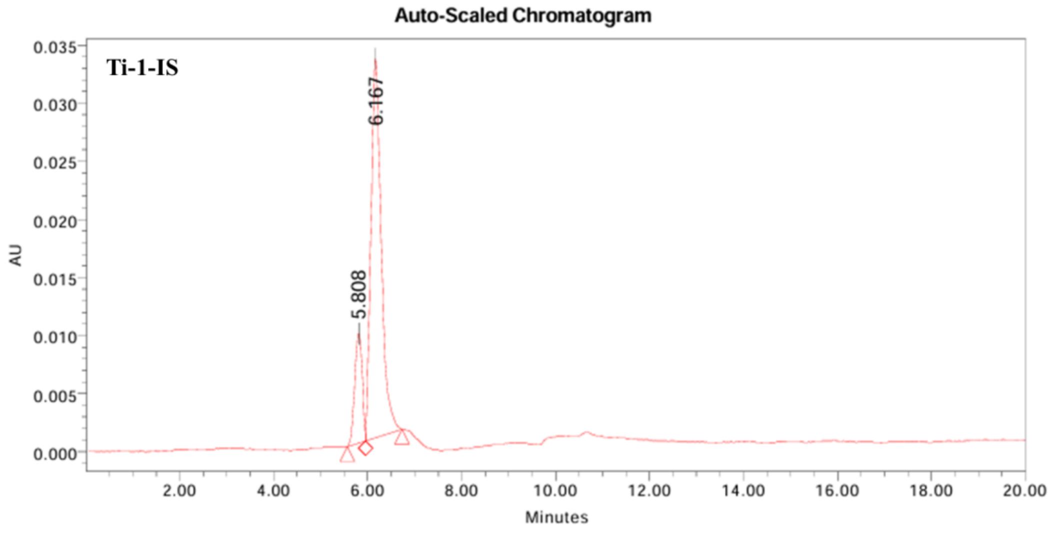

Peak Results

	Name	RT	Area	Height	Amount	Units
1		5.808	104329	9441		
2		6.167	496342	32625		

Figure.S29. HPLC spectrum of complex Ti-1-IS

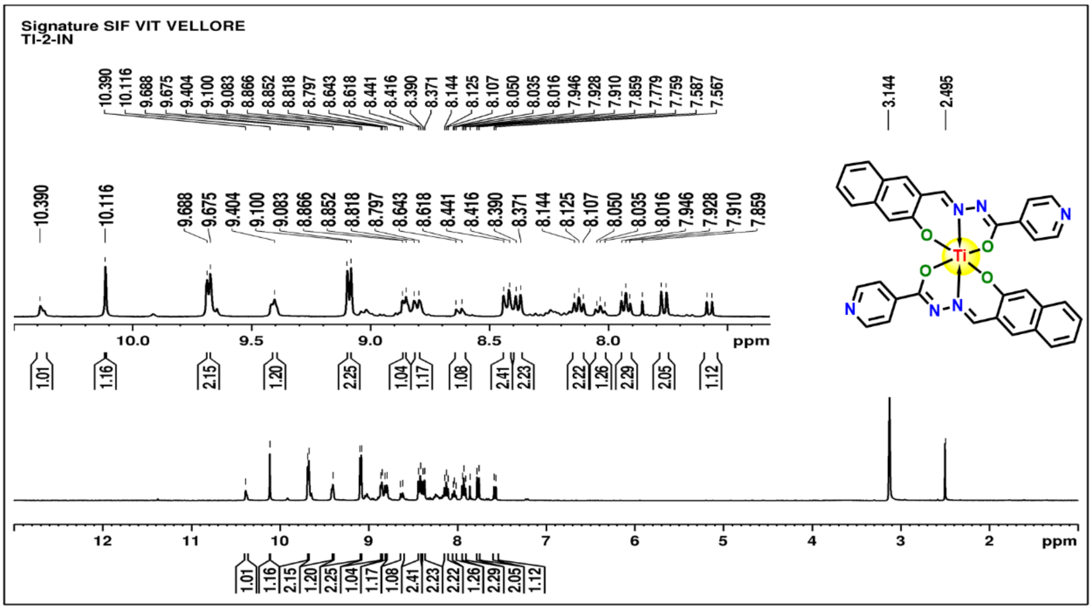

Figure.S30. ^{1}**H NMR spectrum of complex Ti-2-IN (400 MHz, DMSO-d$_6$)**

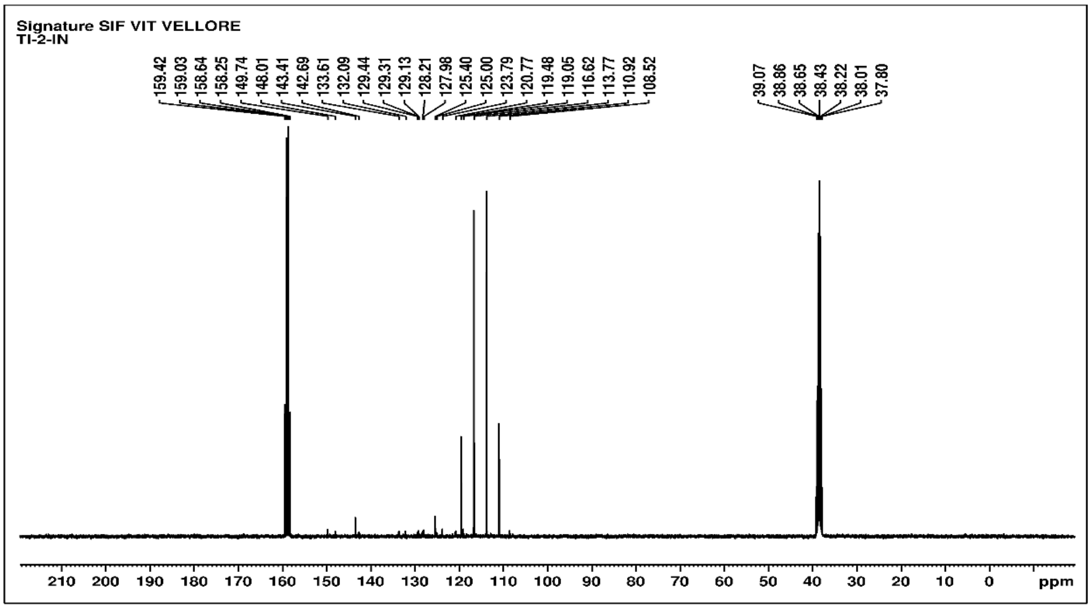

Figure.S31. ^{13}C NMR spectrum of complex Ti-2-IN (400 MHz, DMSO-d_6)

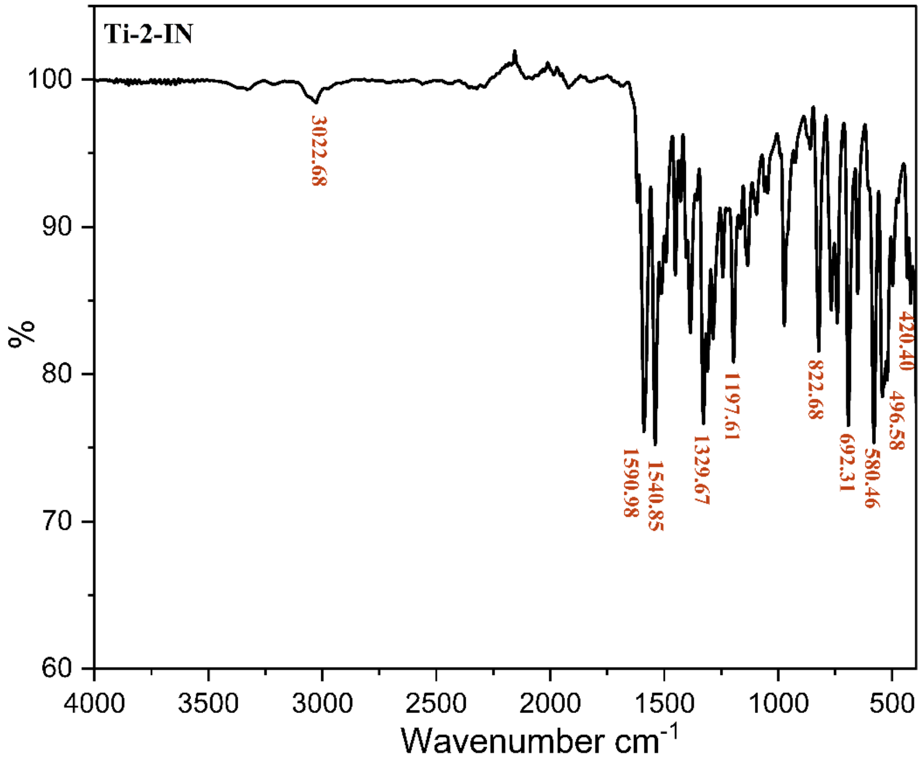

Figure.S32. FTIR spectrum of complex Ti-2-IN

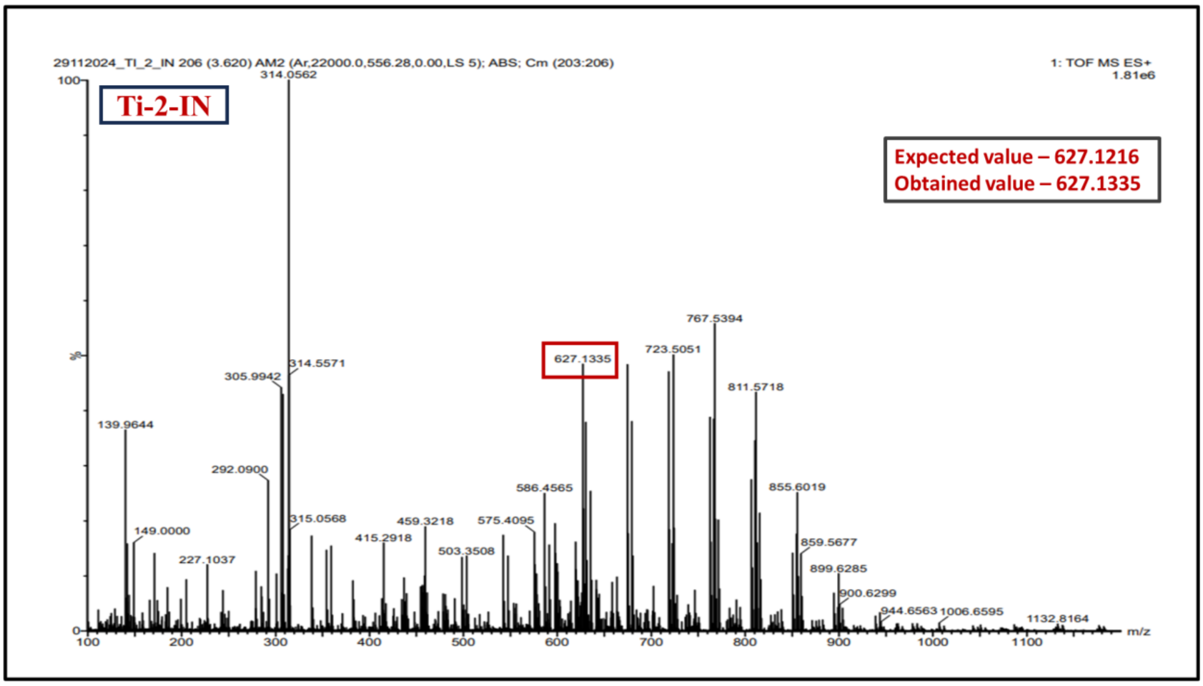

Figure.S33. ESI-MS spectrum of complex Ti-2-IN (400 MHz, DMSO-d_6)

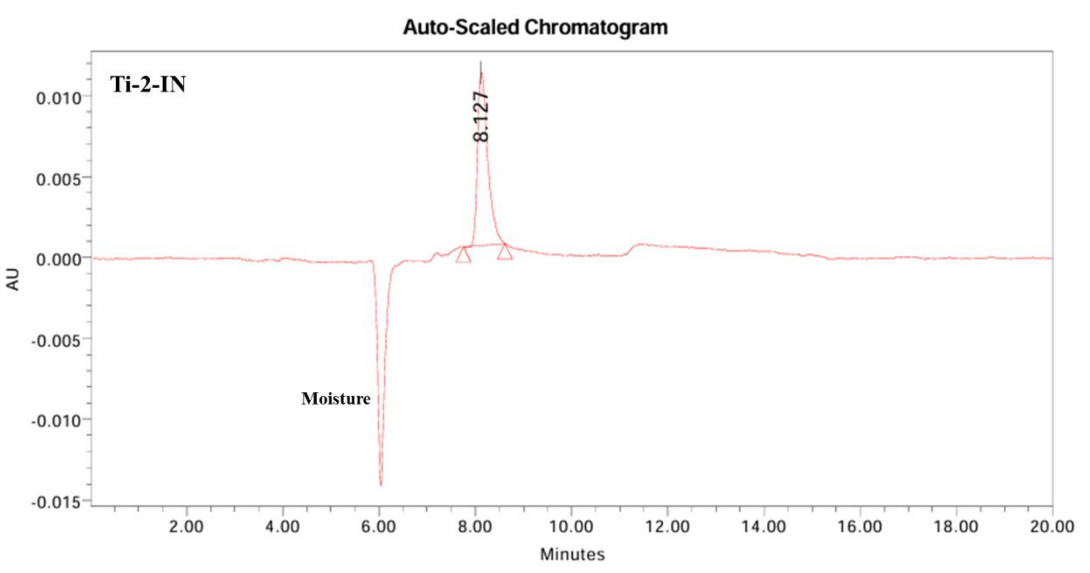

Peak Results

	Name	RT	Area	Height	Amount	Units
1		8.127	157767	10704		

Figure.S34. HPLC spectrum of complex Ti-2-IN

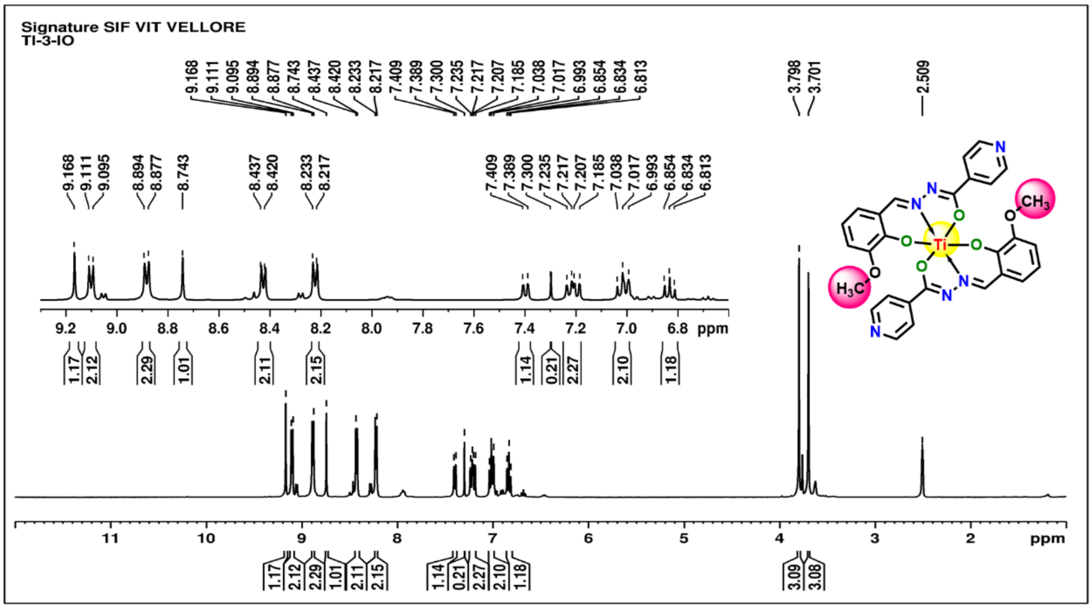

Figure.S35. **^{1}H NMR spectrum of complex Ti-3-IO (400 MHz, DMSO-d_6)**

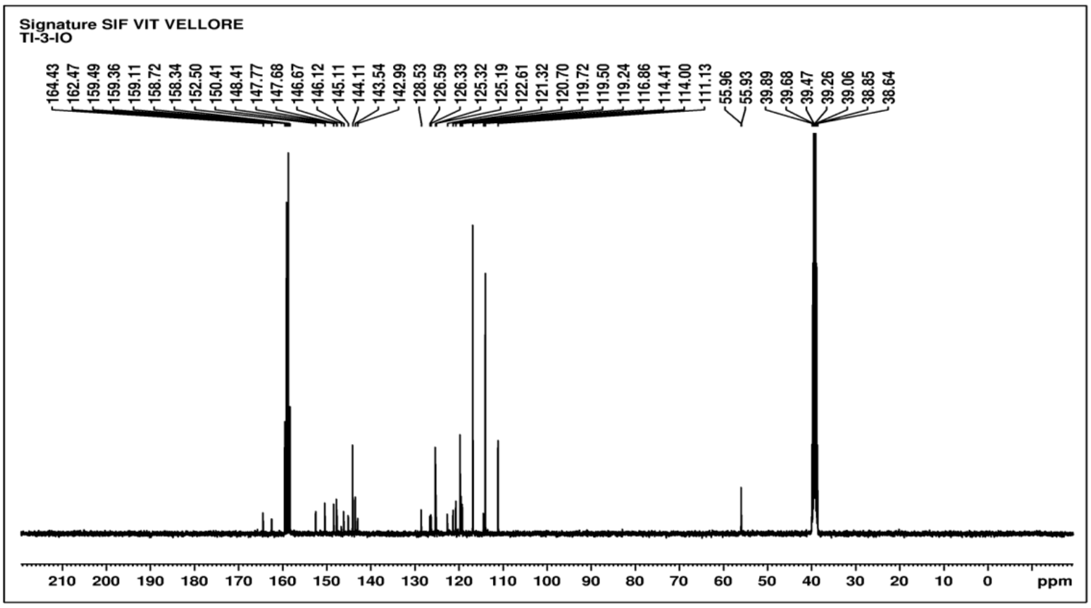

Figure.S36. **^{13}C NMR spectrum of complex Ti-3-IO (400 MHz, DMSO-d_6)**

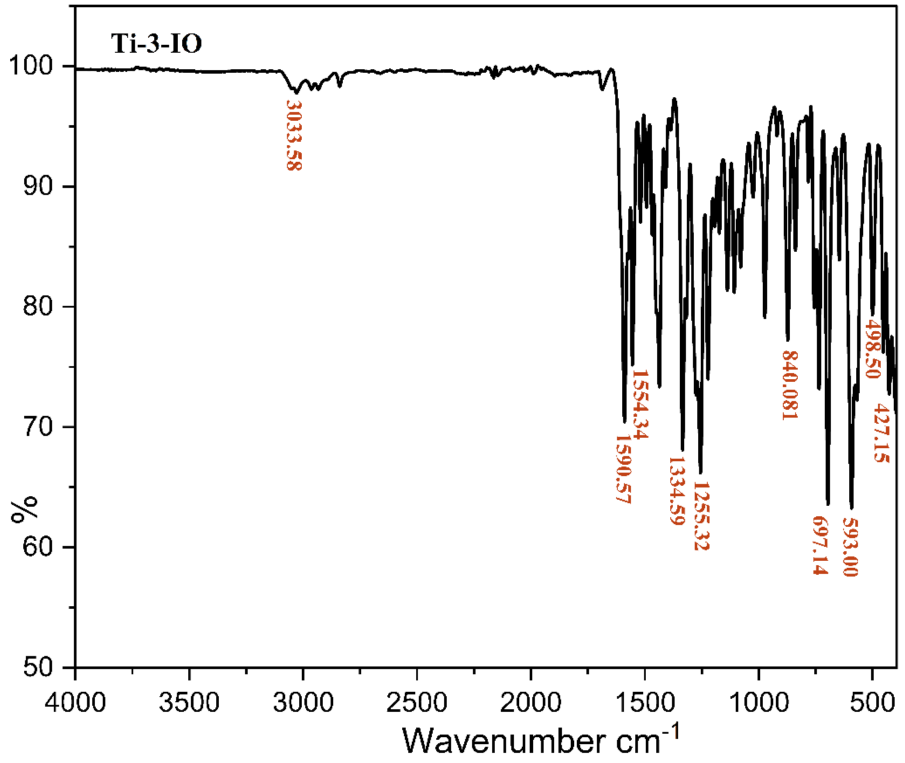

Figure.S37. FTIR spectrum of complex Ti-3-IO

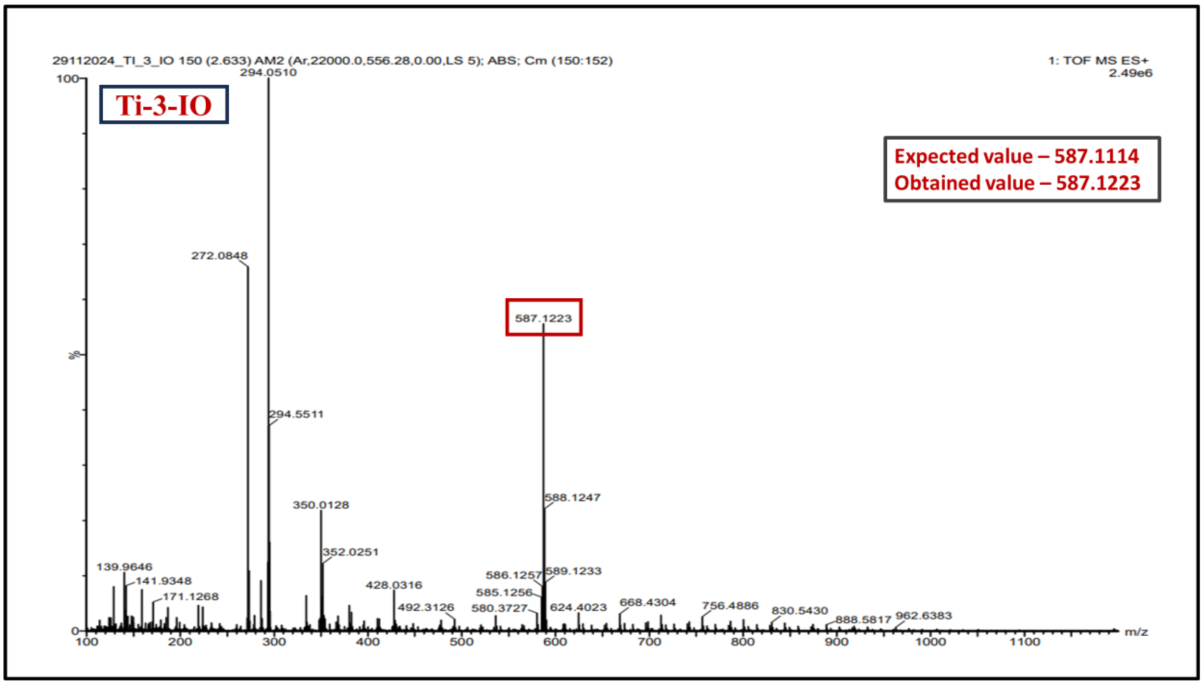

Figure.S38. ESI-MS spectrum of complex Ti-3-IO

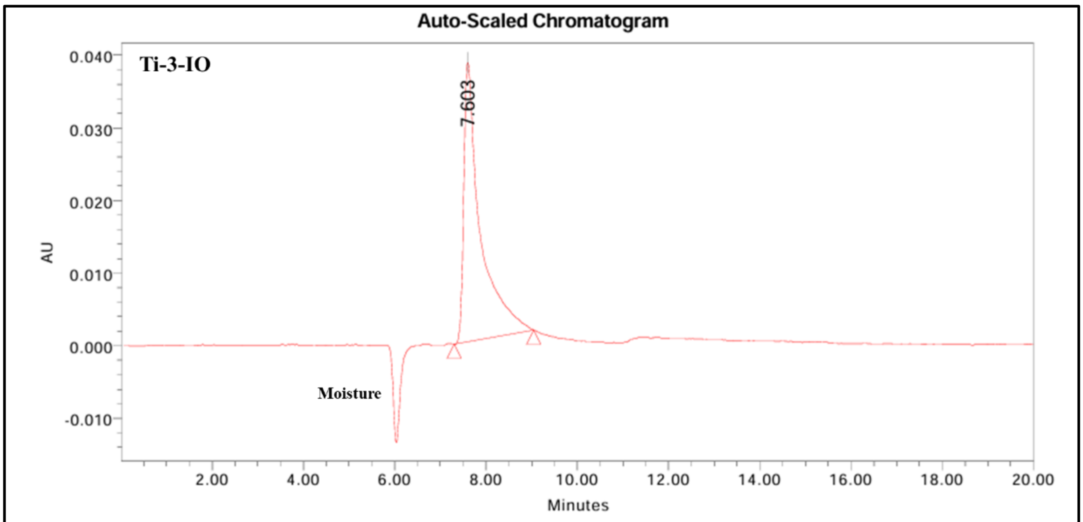

Peak Results

	Name	RT	Area	Height	Amount	Units
1		7.603	976997	38493		

Figure.S39. HPLC spectrum of complex Ti-3-IO

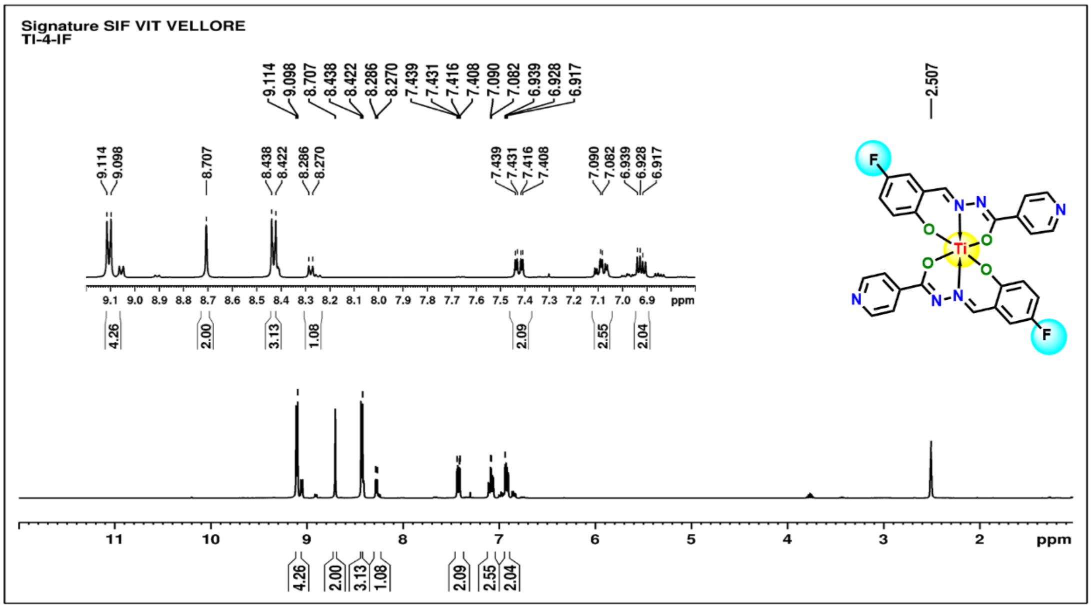

Figure.S40. ^{1}H NMR spectrum of complex Ti-4-IF(400 MHz, DMSO-d_6)

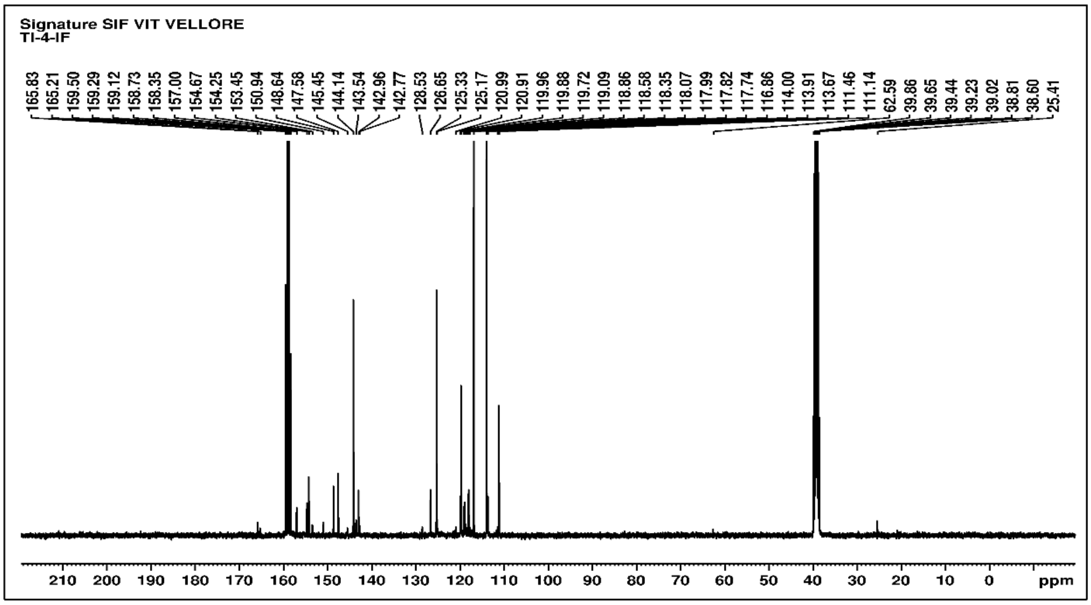

Figure.S41. ^{13}C **NMR spectrum of complex Ti-4-IF(400 MHz, DMSO-d$_6$)**

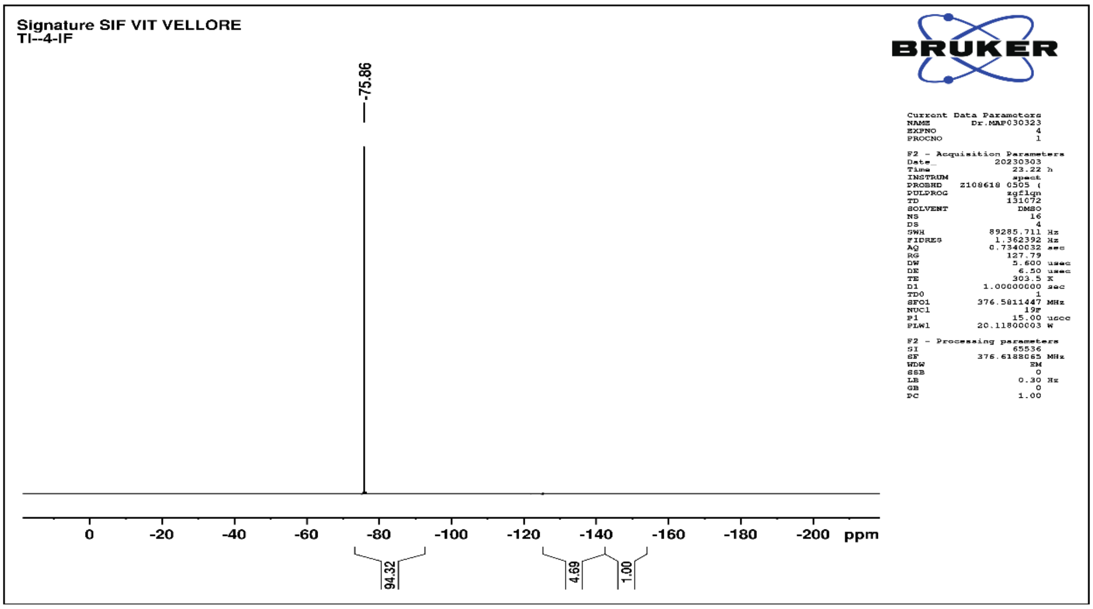

Figure.S42. ^{19}F **NMR spectrum of complex Ti-4-IF(400 MHz, DMSO-d$_6$)**

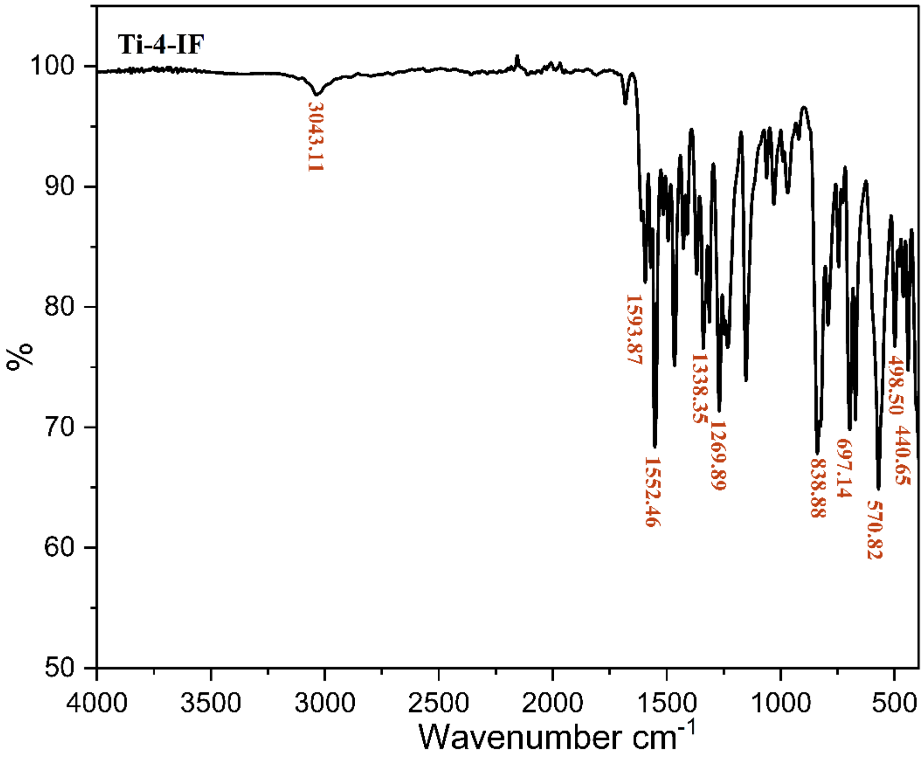

Figure.S43. FTIR spectrum of complex Ti-4-IF

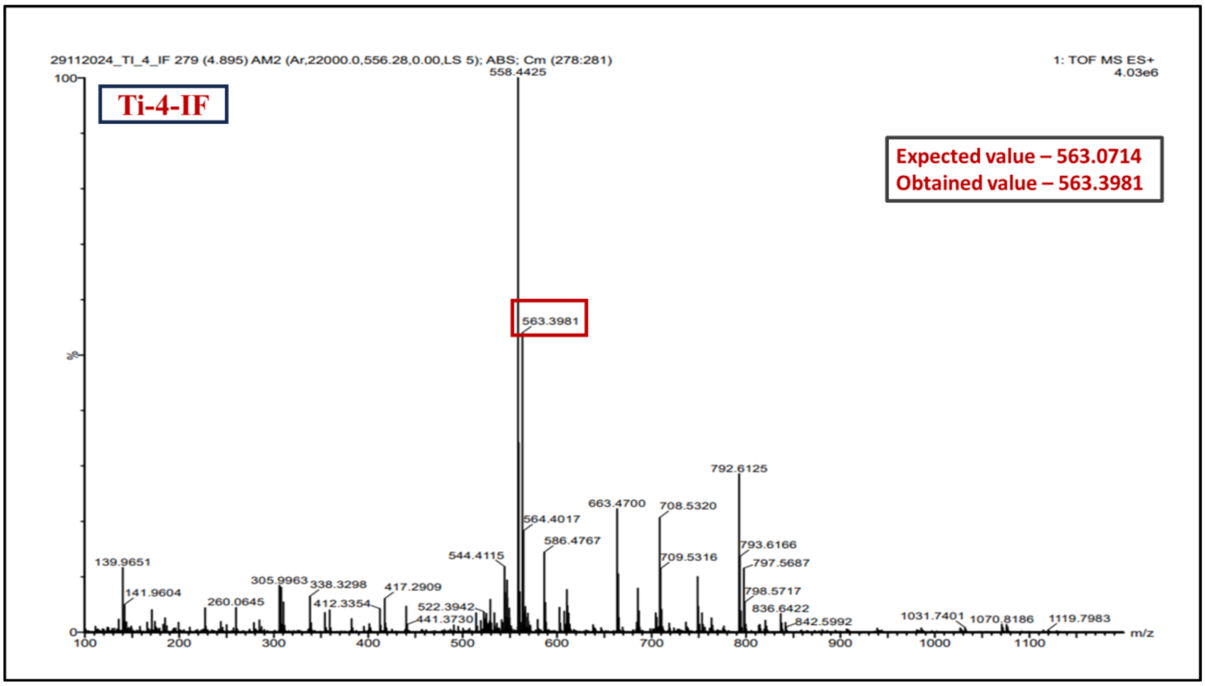

Figure.S44. ESI-MS spectrum of complex Ti-4-IF

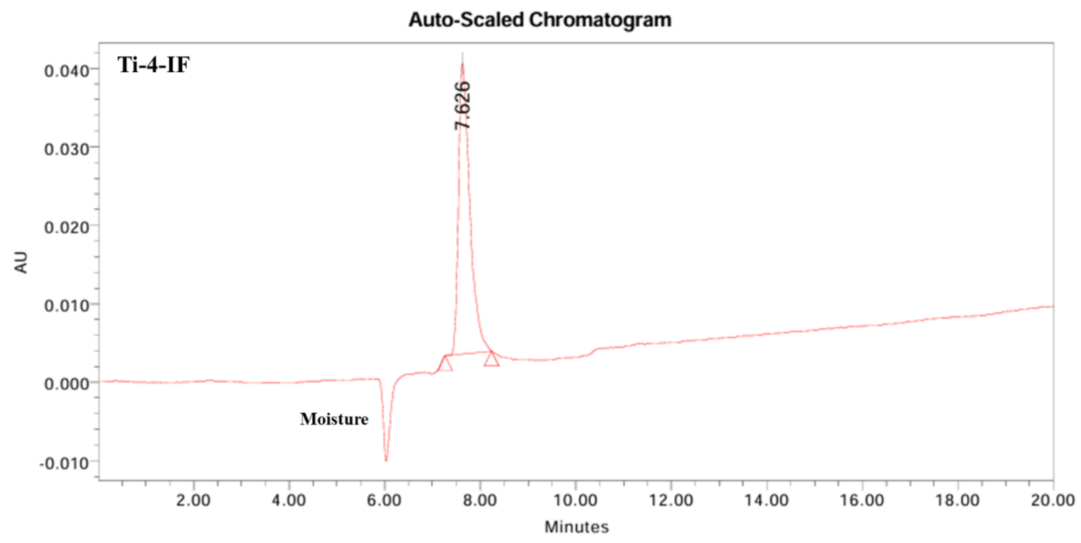

Peak Results

	Name	RT	Area	Height	Amount	Units
1		7.626	616721	37208		

Figure.S45. HPLC spectrum of complex Ti-4-IF

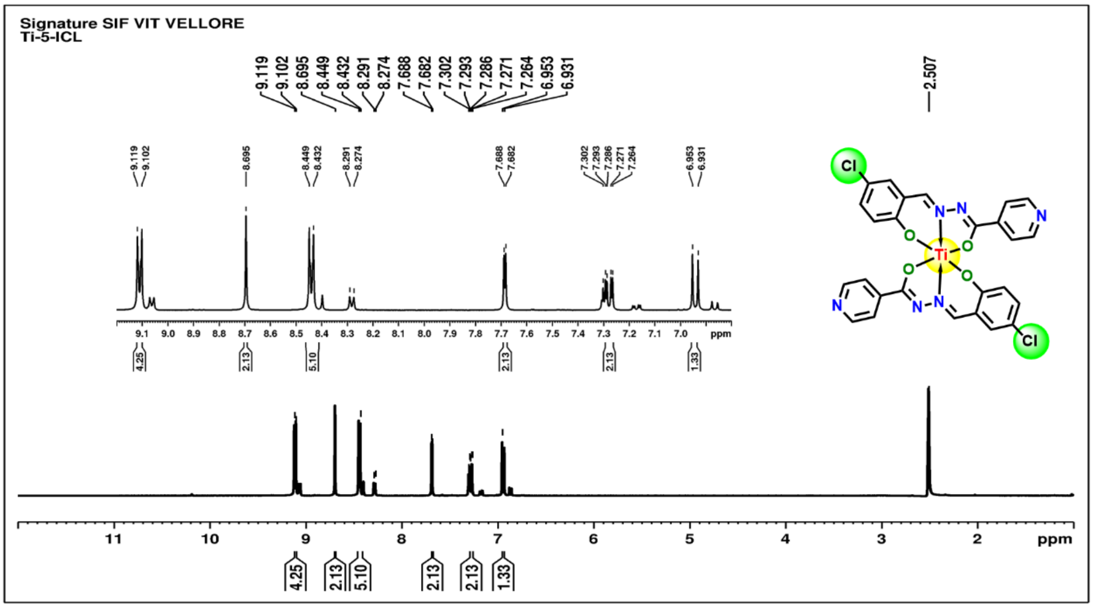

Figure.S46. ^{1}H NMR spectrum of complex Ti-5-ICl (400 MHz, DMSO-d_6)

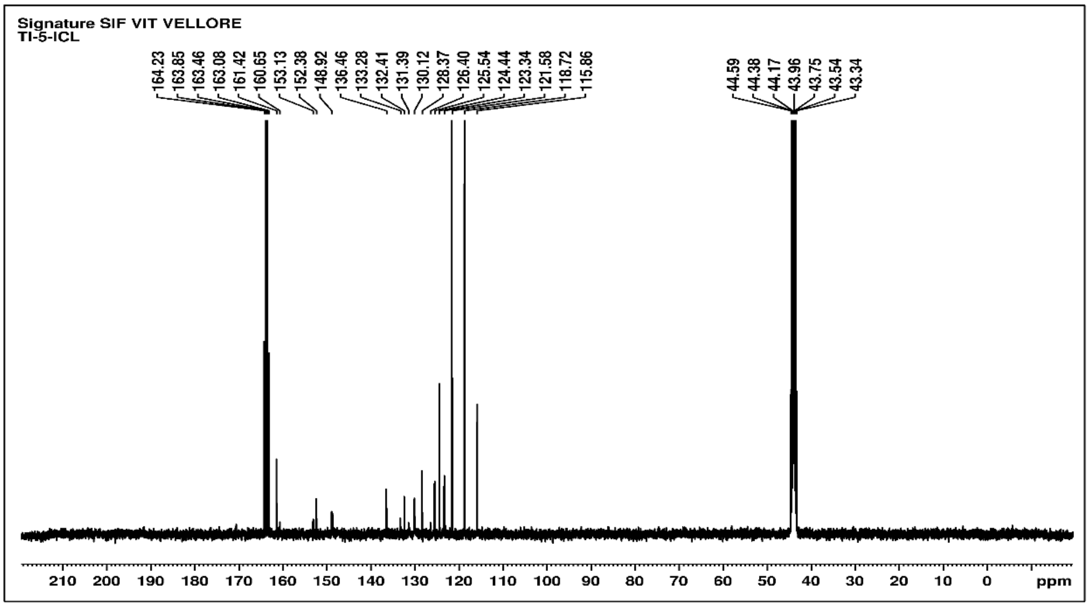

Figure.S47. ^{13}C NMR spectrum of complex Ti-5-ICl (400 MHz, DMSO-d$_6$)

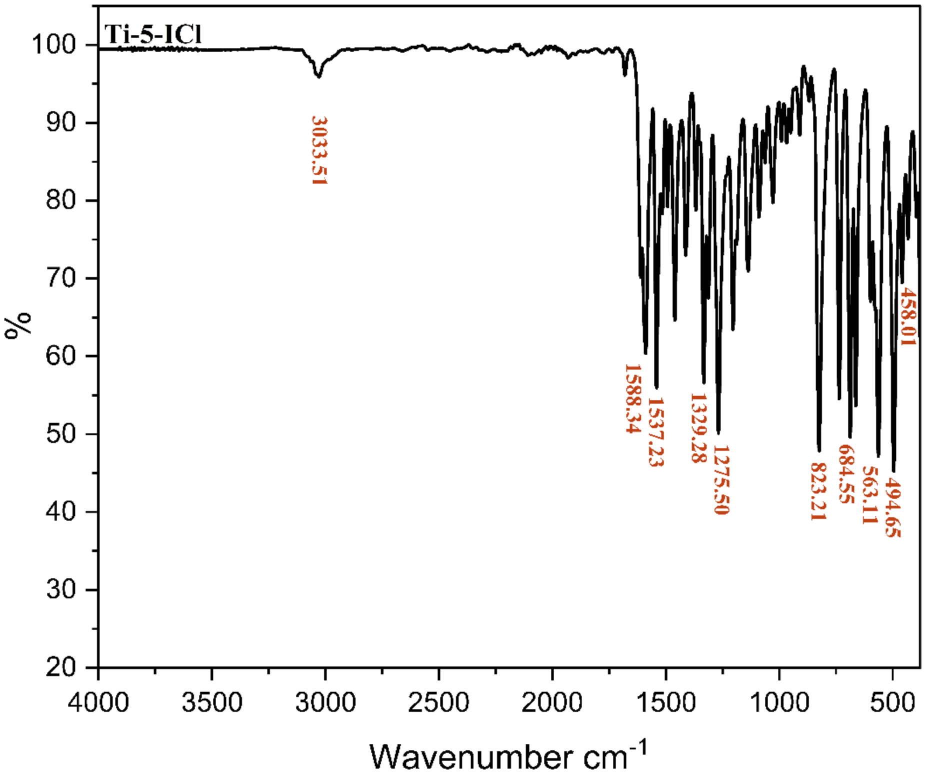

Figure.S48. FTIR spectrum of complex Ti-5-ICl

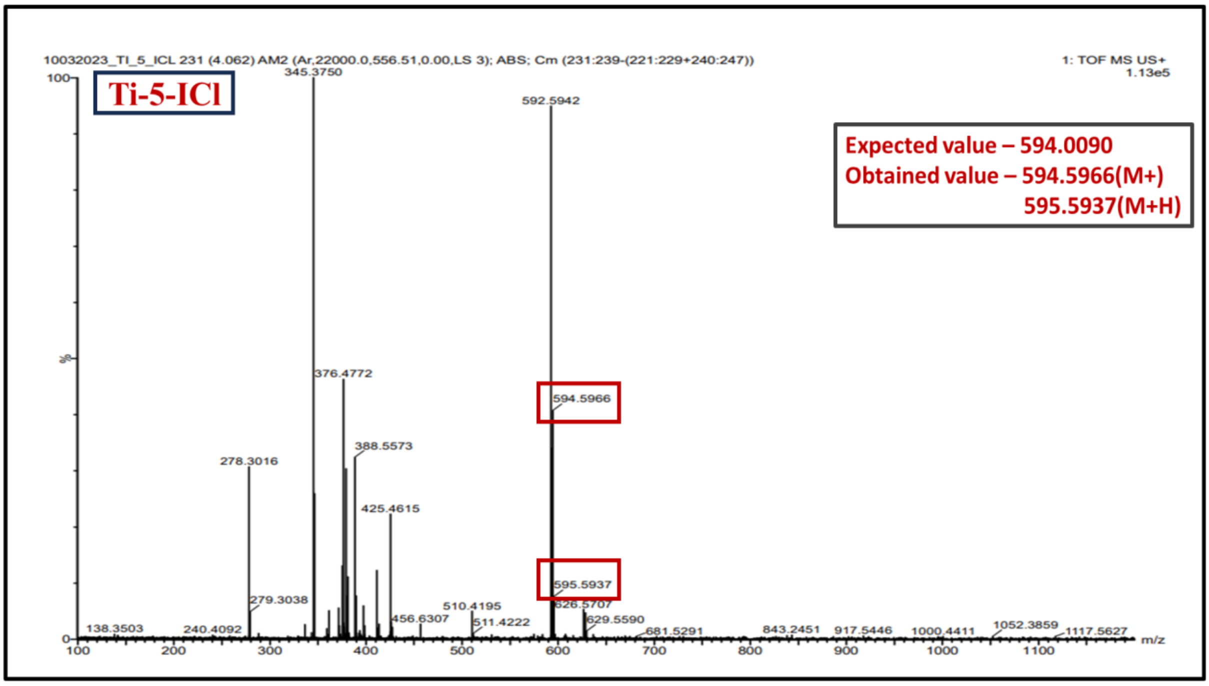

Figure.S49. ESI-MS spectrum of complex Ti-5-ICl

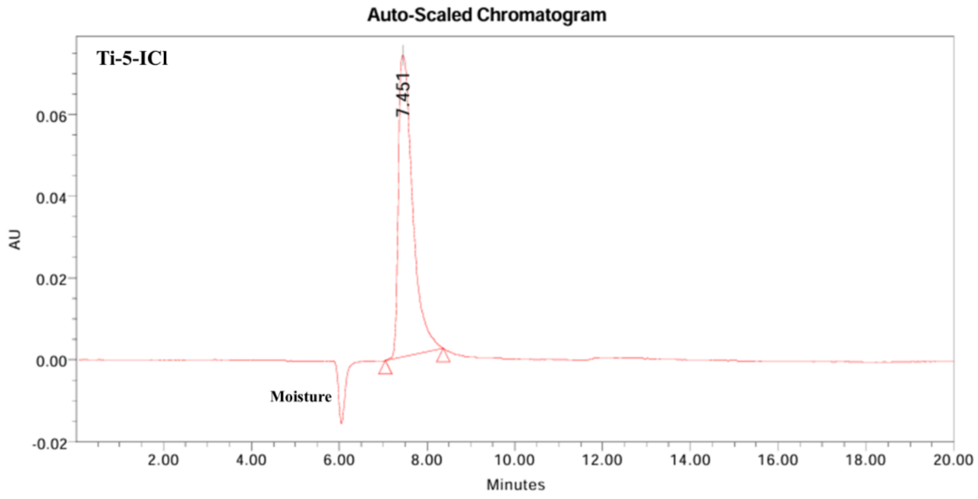

Peak Results

	Name	RT	Area	Height	Amount	Units
1		7.451	1666481	73690		

Figure.S50. HPLC spectrum of complex Ti-5-ICl

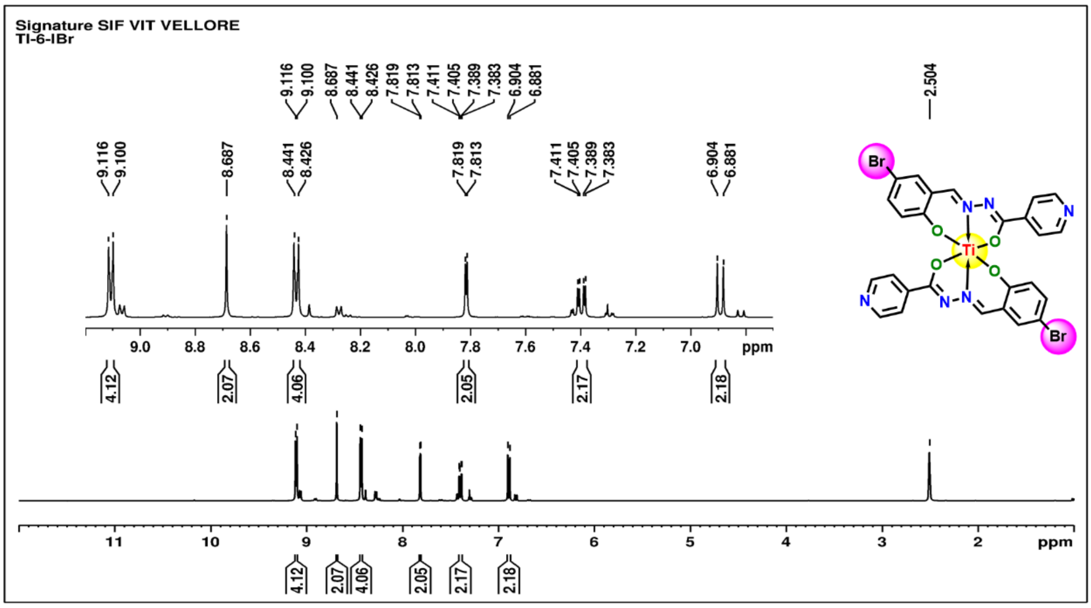

Figure.S51. ^{1}H NMR spectrum of complex Ti-6-IBr (400 MHz, DMSO-d_6)

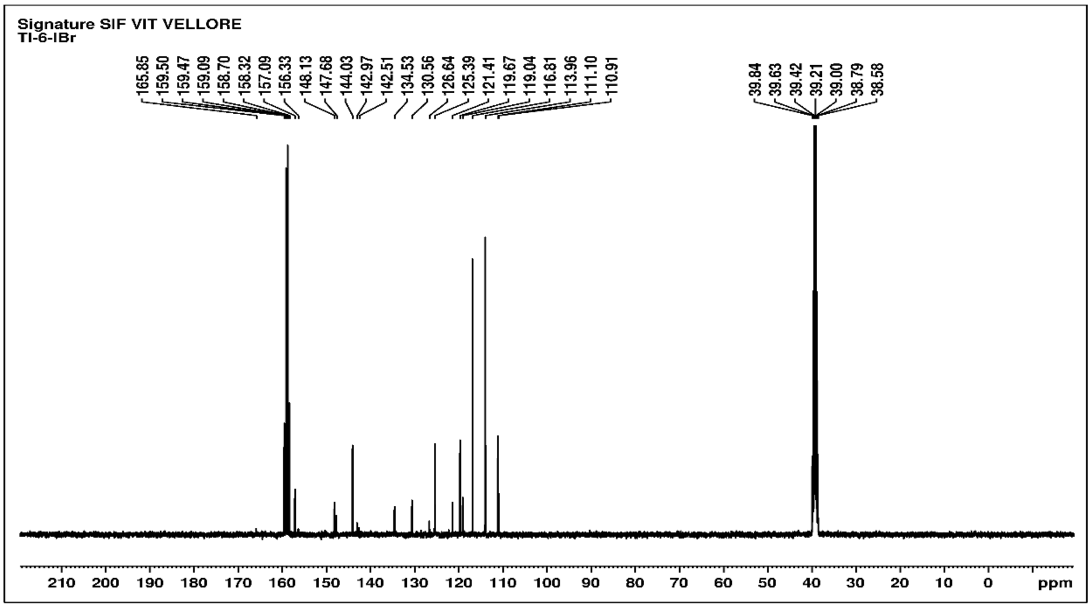

Figure.S52. ^{13}C NMR spectrum of complex Ti-6-IBr (400 MHz, DMSO-d_6)

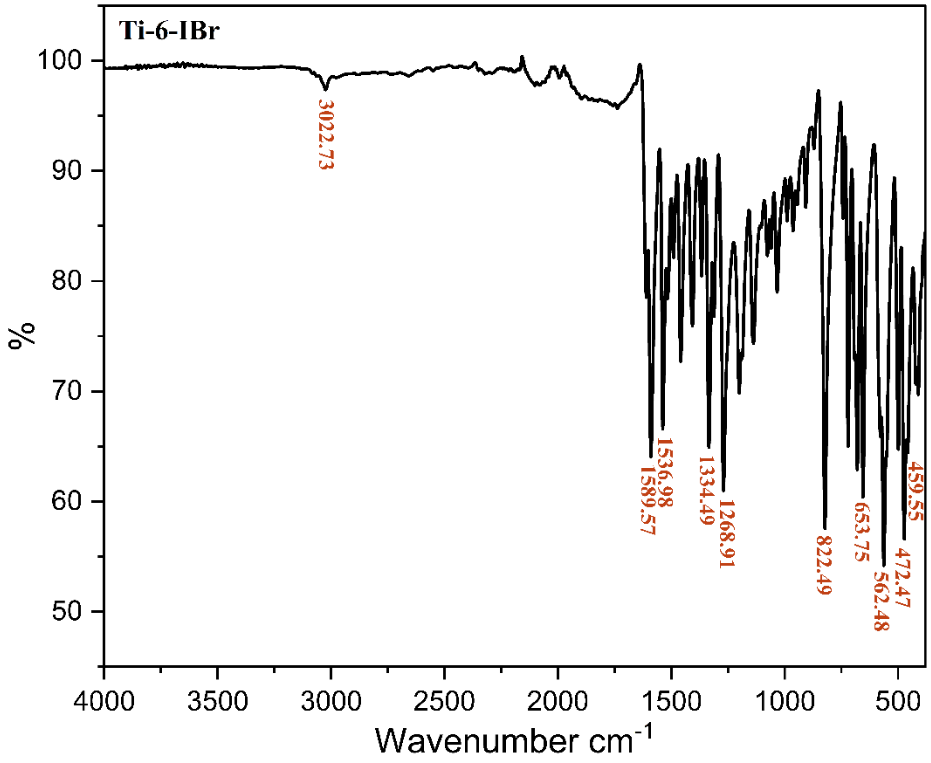

Figure.S53. FTIR spectrum of complex Ti-6-IBr

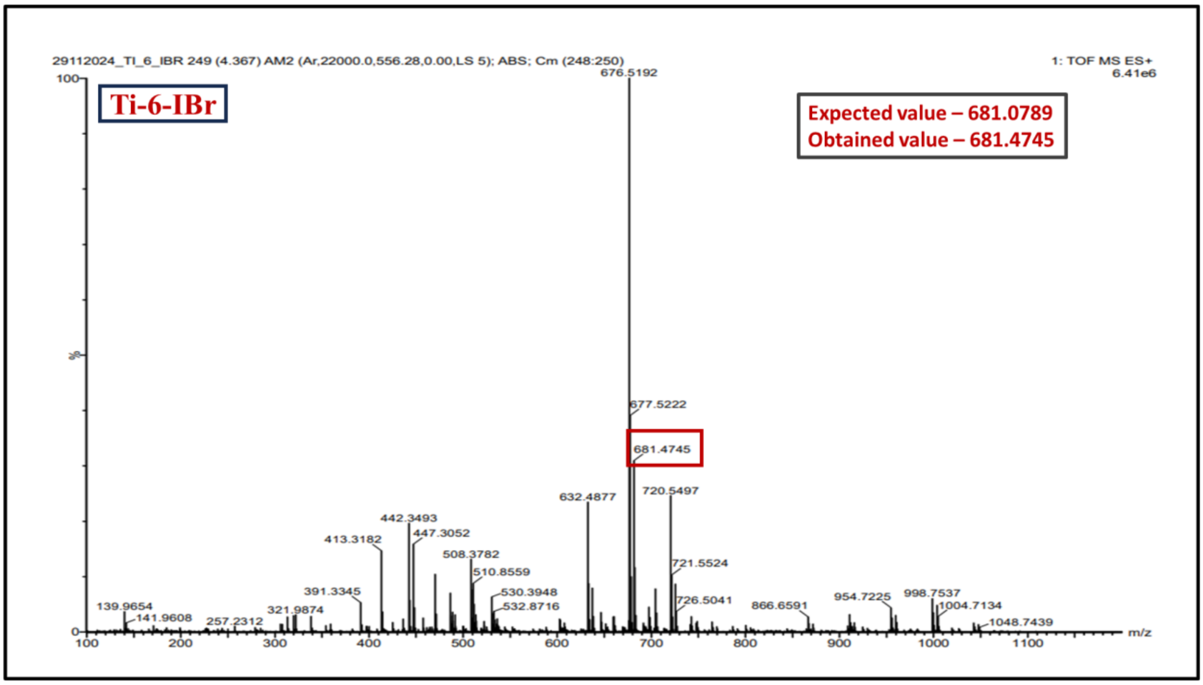

Figure.S54. ESI-MS spectrum of complex Ti-6-IBr

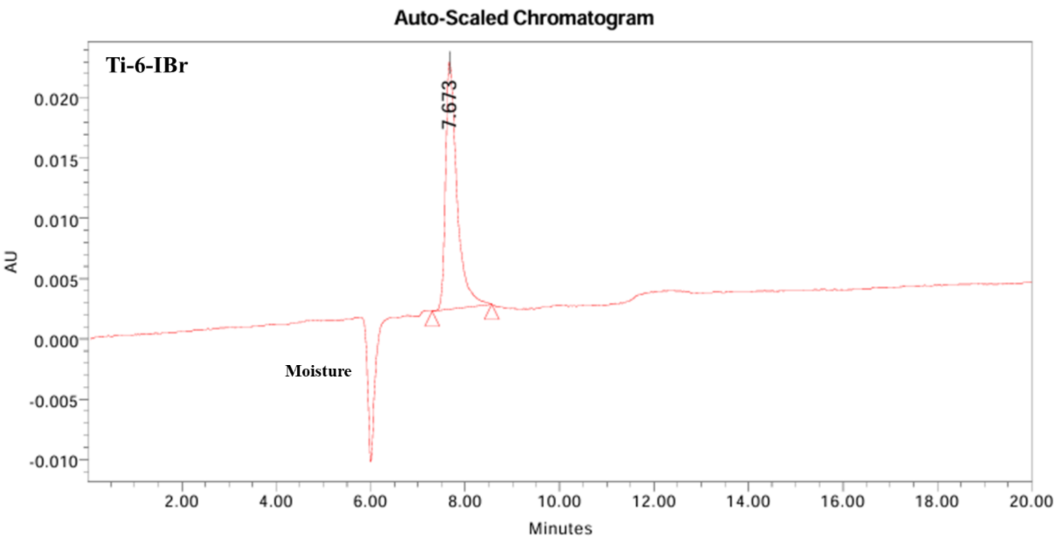

Peak Results

	Name	RT	Area	Height	Amount	Units
1		7.673	344930	20554		

Figure.S55. HPLC spectrum of complex Ti-6-IBr

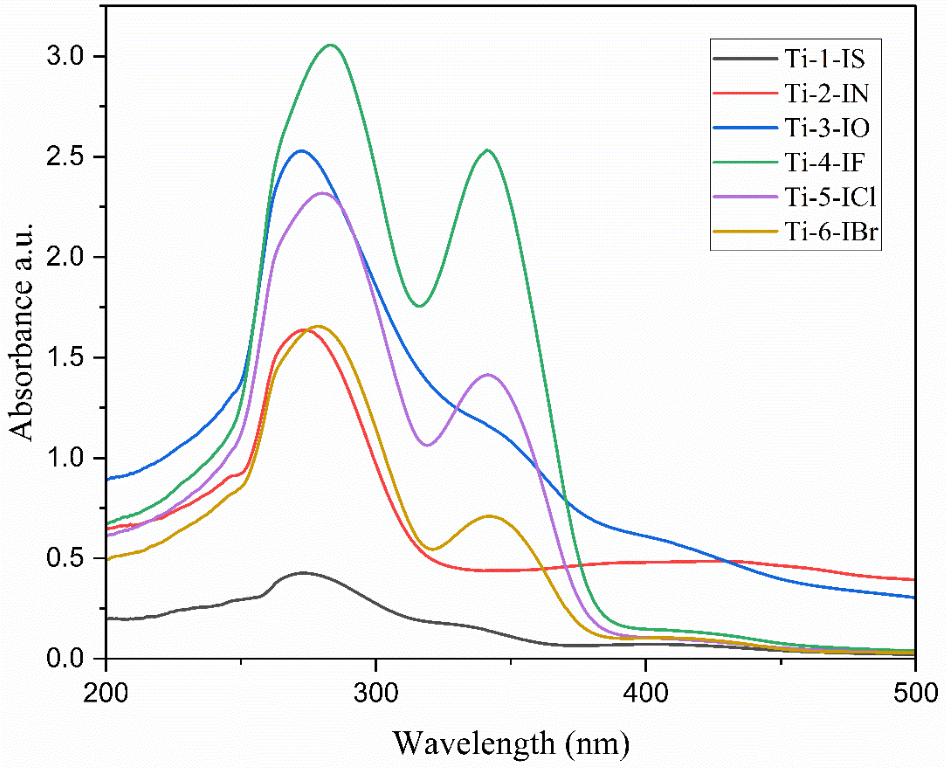

Fig.S56. UV-Vis Spectra of Ti(IV) complexes in DMSO: H_2O (1:9)

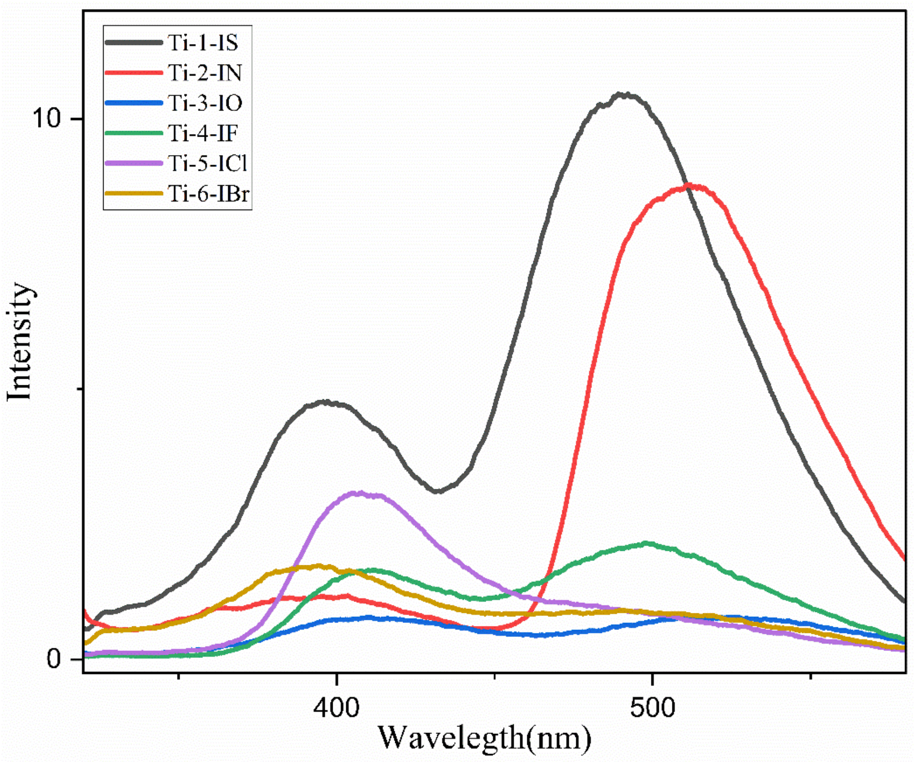

Fig.S57. Fluorescence Spectra of Ti(IV) complexes in DMSO: H_2O (1:9)

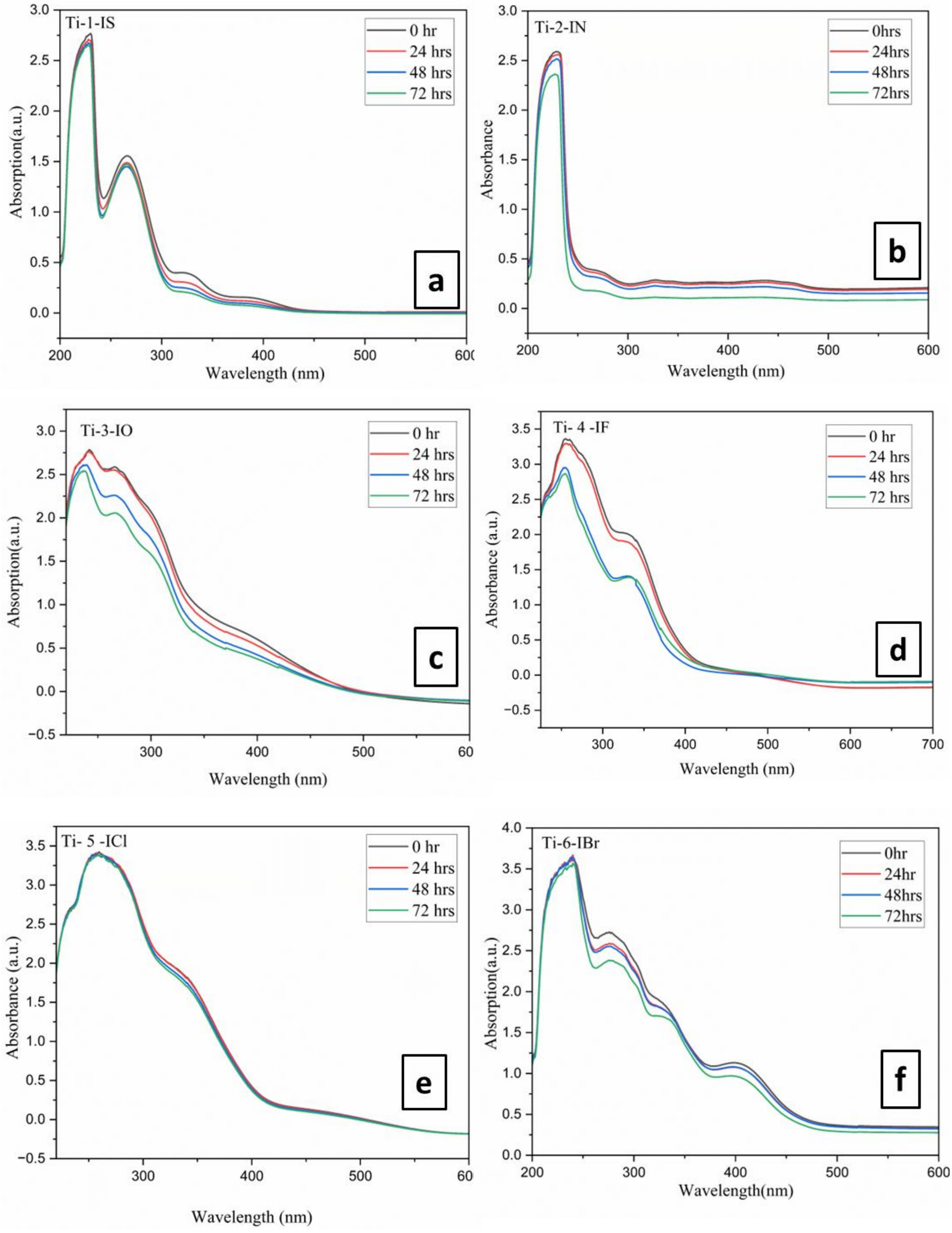

Fig.S58. UV-Vis stability study of Ti(IV) complexes in 1:9 DMSO: H_2O medium

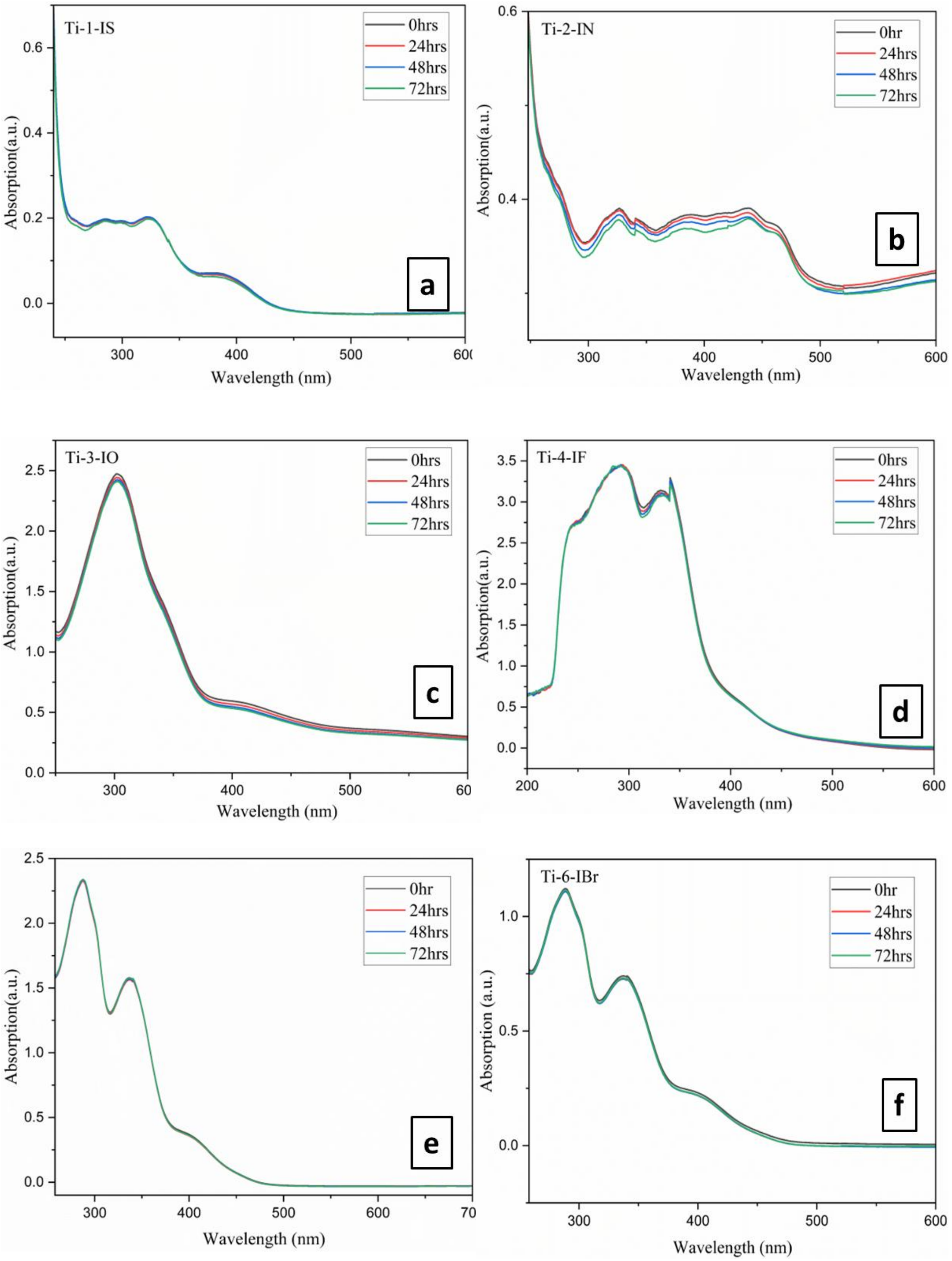

Fig.S59. UV-Vis stability study of Ti(IV) complexes in GSH medium

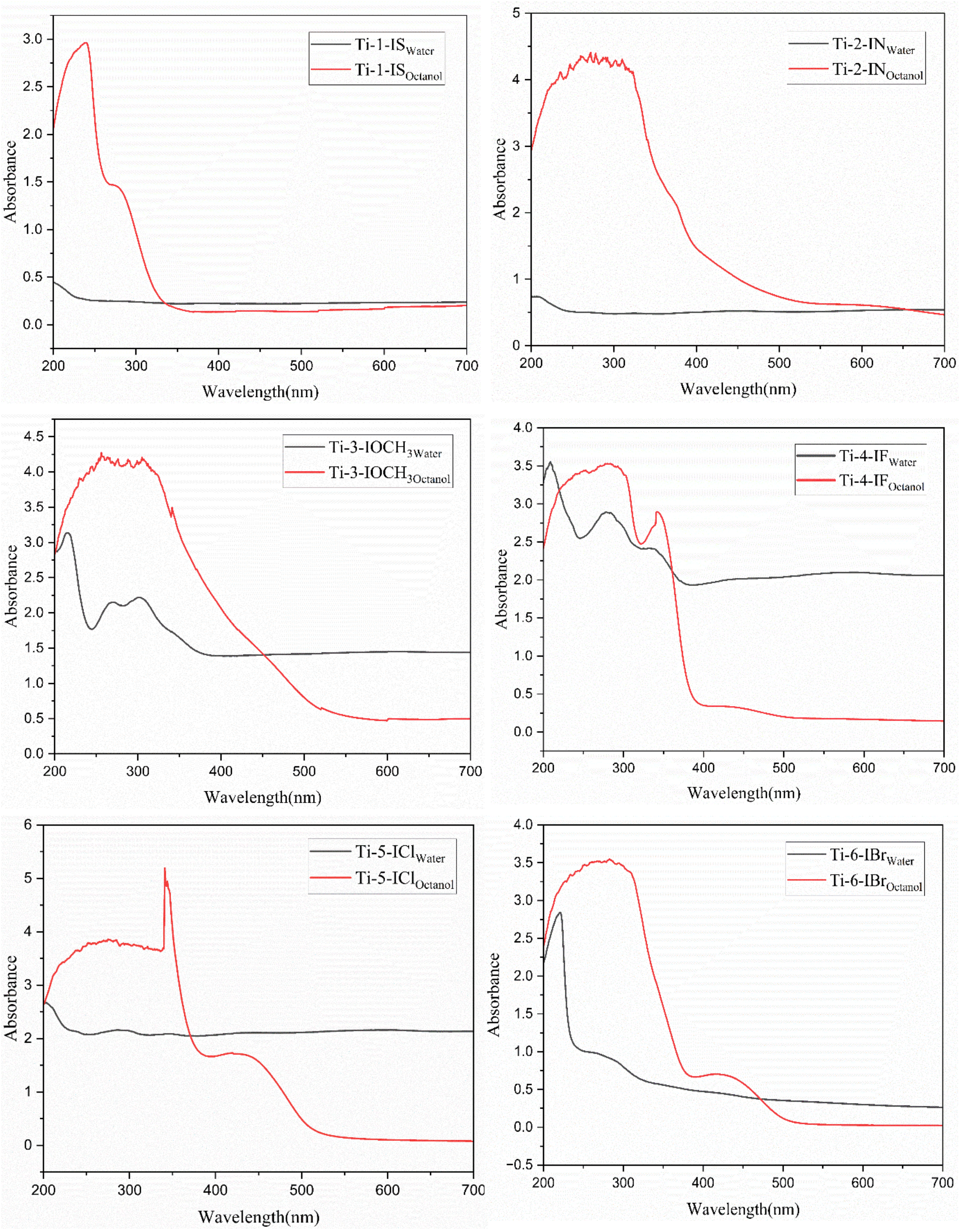

Fig. S60. UV-visible spectra of Ti(IV) complexes for Lipophilicity study of complexes in octanol: water

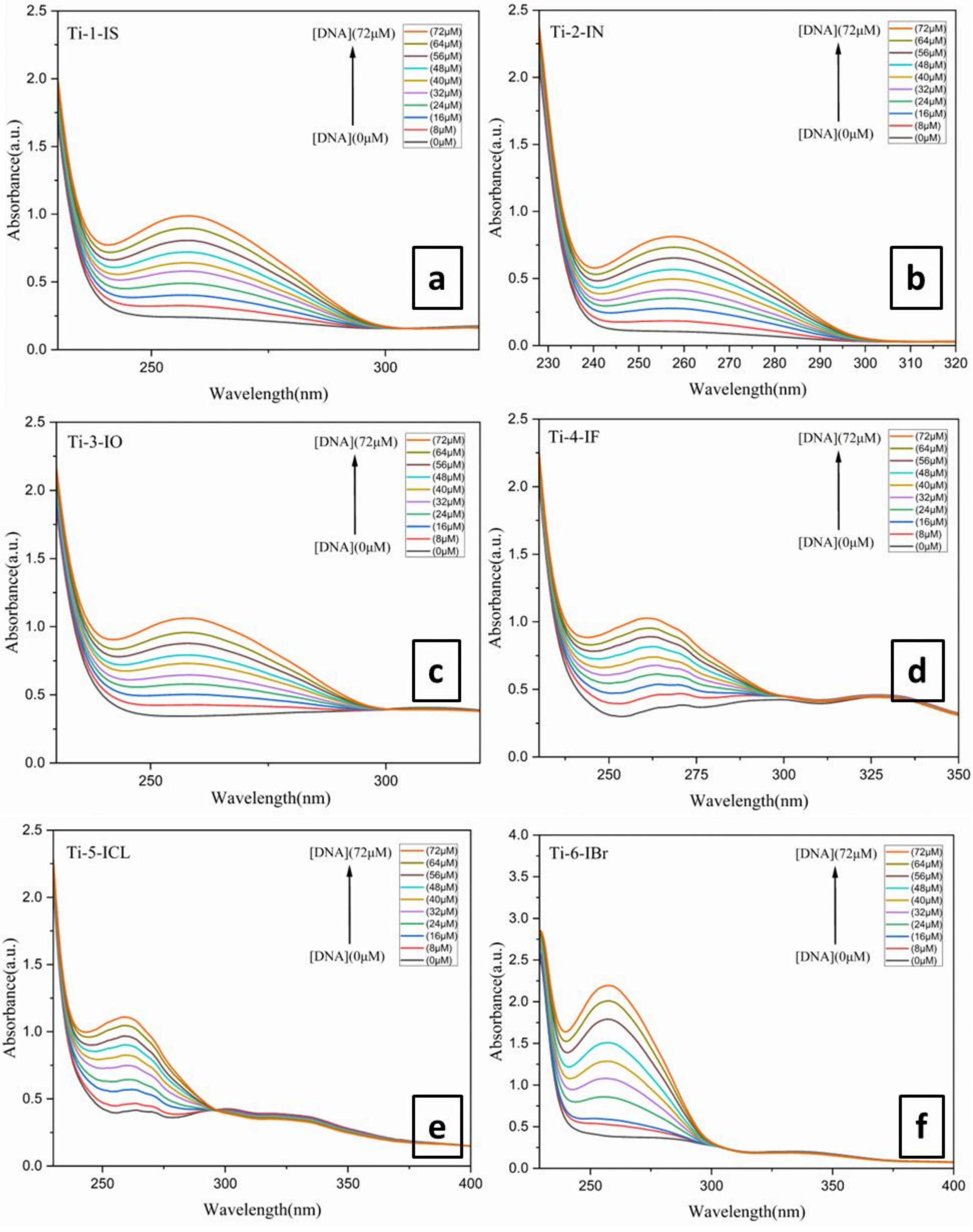

Fig.S61. UV-Visible spectra of DNA Binding studies of Ti(IV) complexes

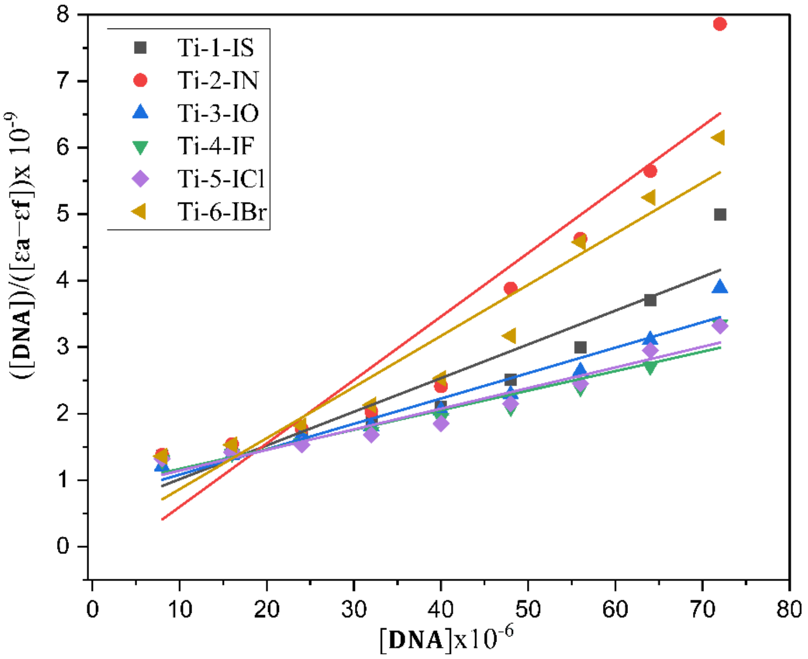

Fig.S62. Linear plots of DNA UV-binding studies of Ti(IV) complexes

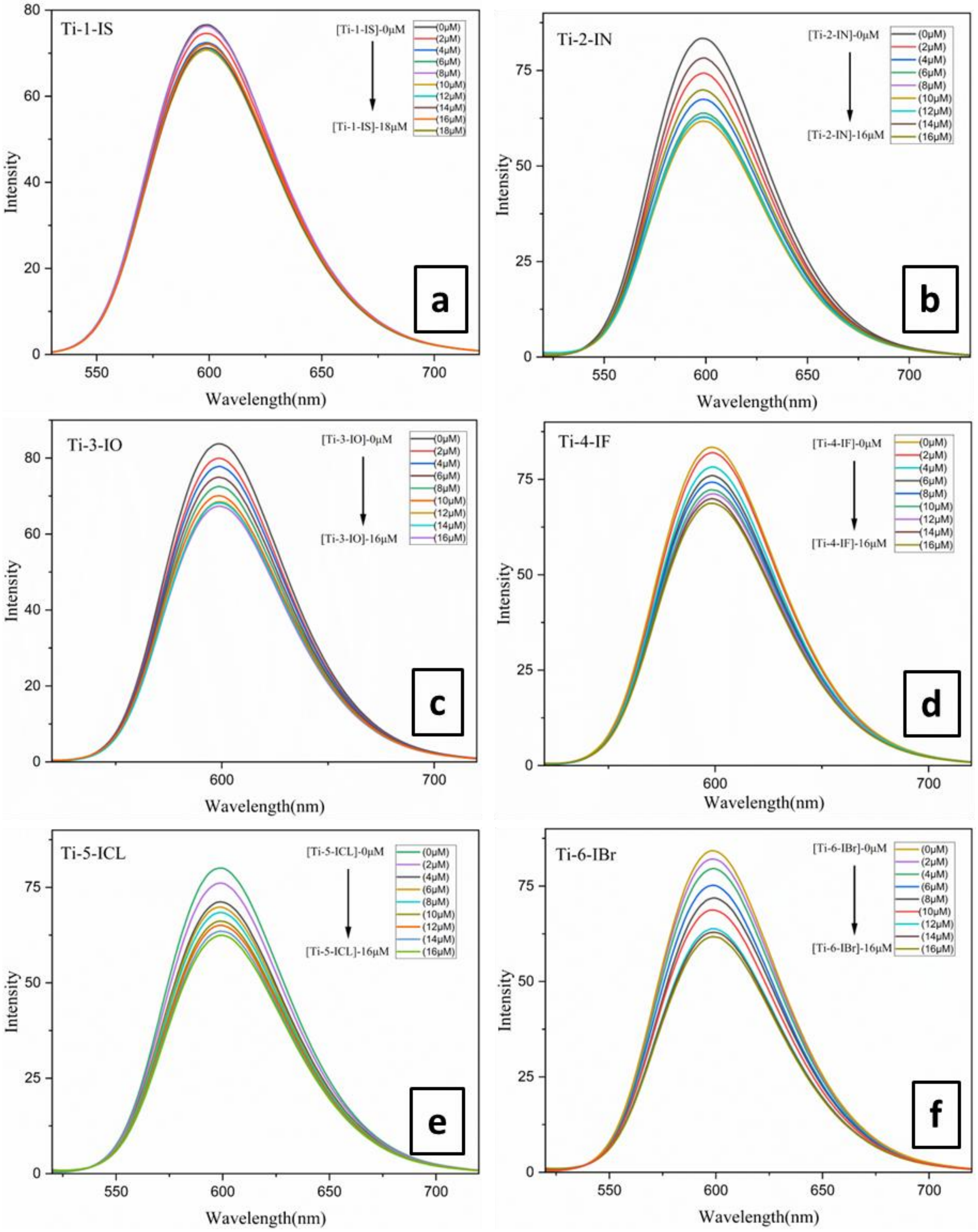

Fig.S63. Fluorescence quenching spectra of DNA with increasing concentration of Ti(IV) complexes

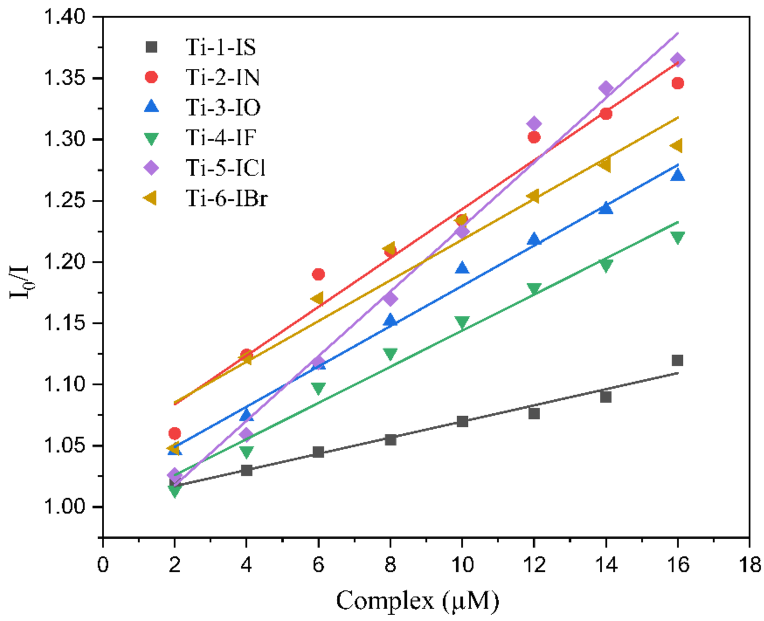

Fig.S64. Stern-Volmer plots of I_0/I vs. Ti(IV) complexes

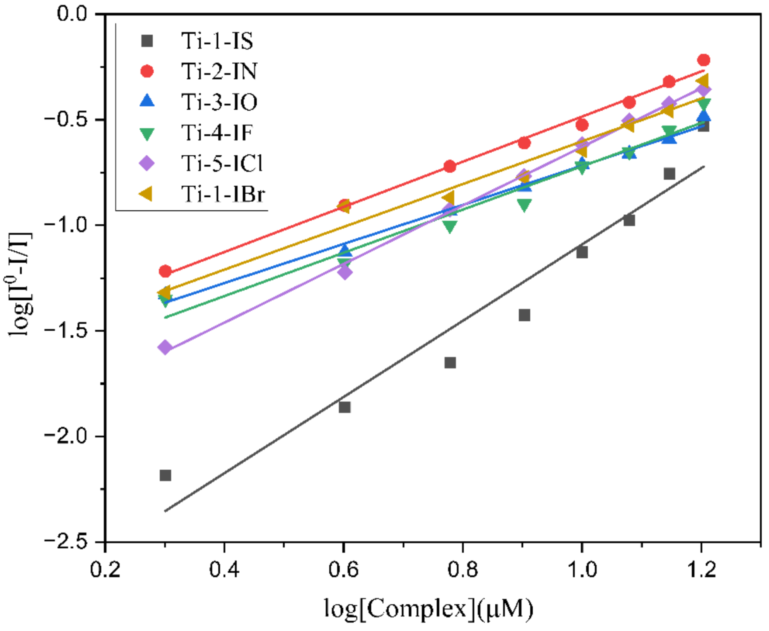

Fig.S65. Scatchard plot of log([I_0-I]/I) vs. log [Ti(IV) complexes]

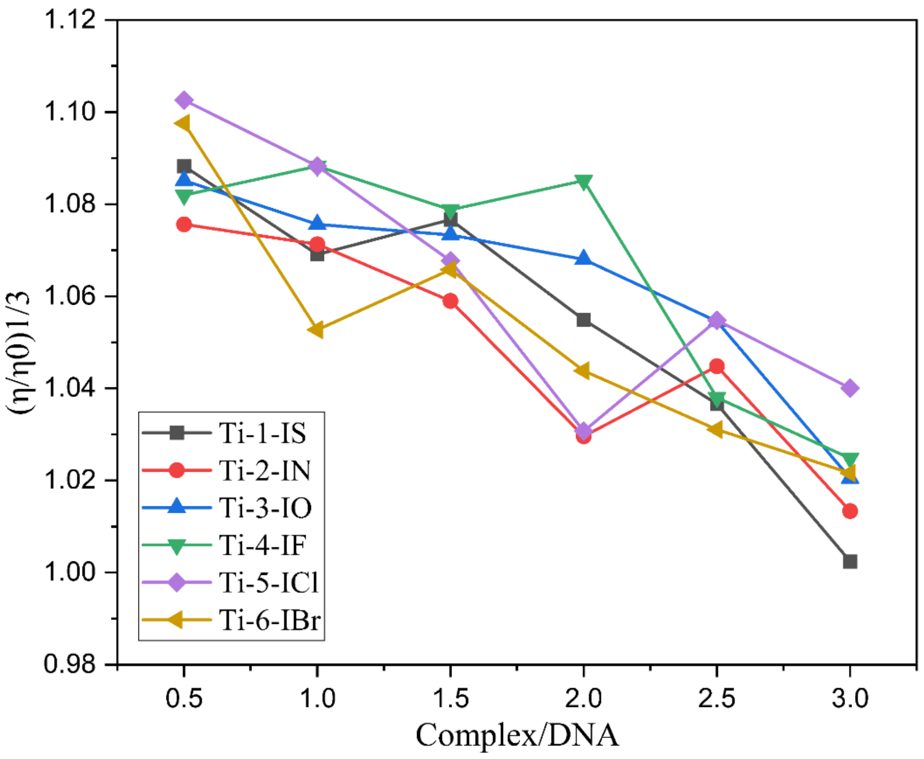

Fig.S66. Viscosity Studies of Ti(IV) complexes

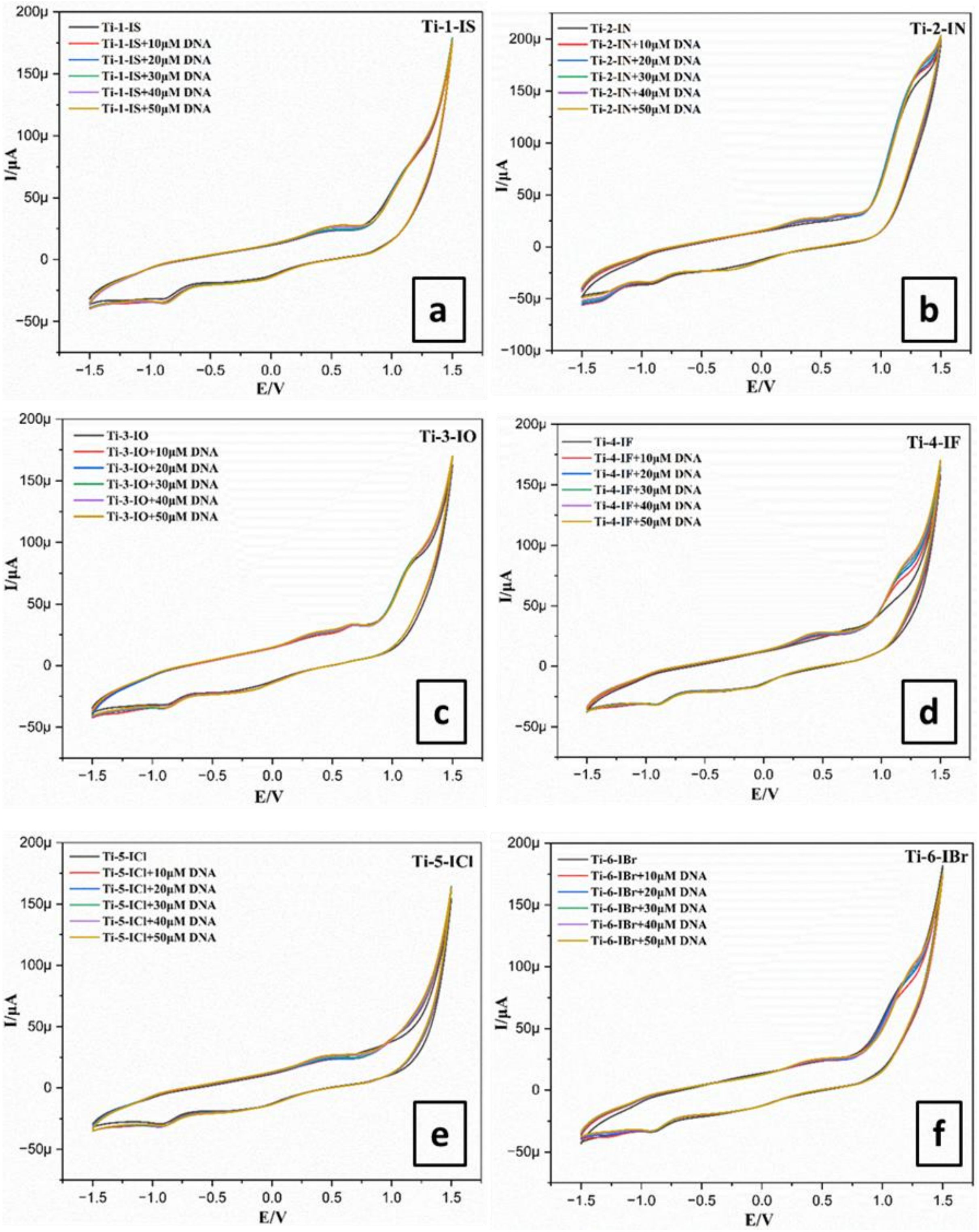

Fig.S67. Cyclic voltammetry DNA binding Studies of Ti(IV) complexes

Table S1: Oxidation and reduction peaks obtained from Cyclic Voltammograms of Ti(IV) complexes with CT-DNA(0–50 µM)

Complexes	Oxidation peak	Reduction peak
**Ti-1-IS**	-0.877, 0.533, 1.099	-0.860, -0.117, 1.327
**Ti-2-IN**	-1.328, -0.924, -0.233	-0.920, -0.335
**Ti-3-IO**	-0.892, -0.264	-0.900, -0.355, -0.665, 1.161
**Ti-4-IF**	-0.892, -0.417, 1.16	-0.892, -0.148, 0.859
**Ti-5-ICl**	-0.932, 0.425	-0.916, -0.218, 0.750
**Ti-6-IBr**	-0.908, 0.440, 1.239	-1.249, -0.908, -0.055

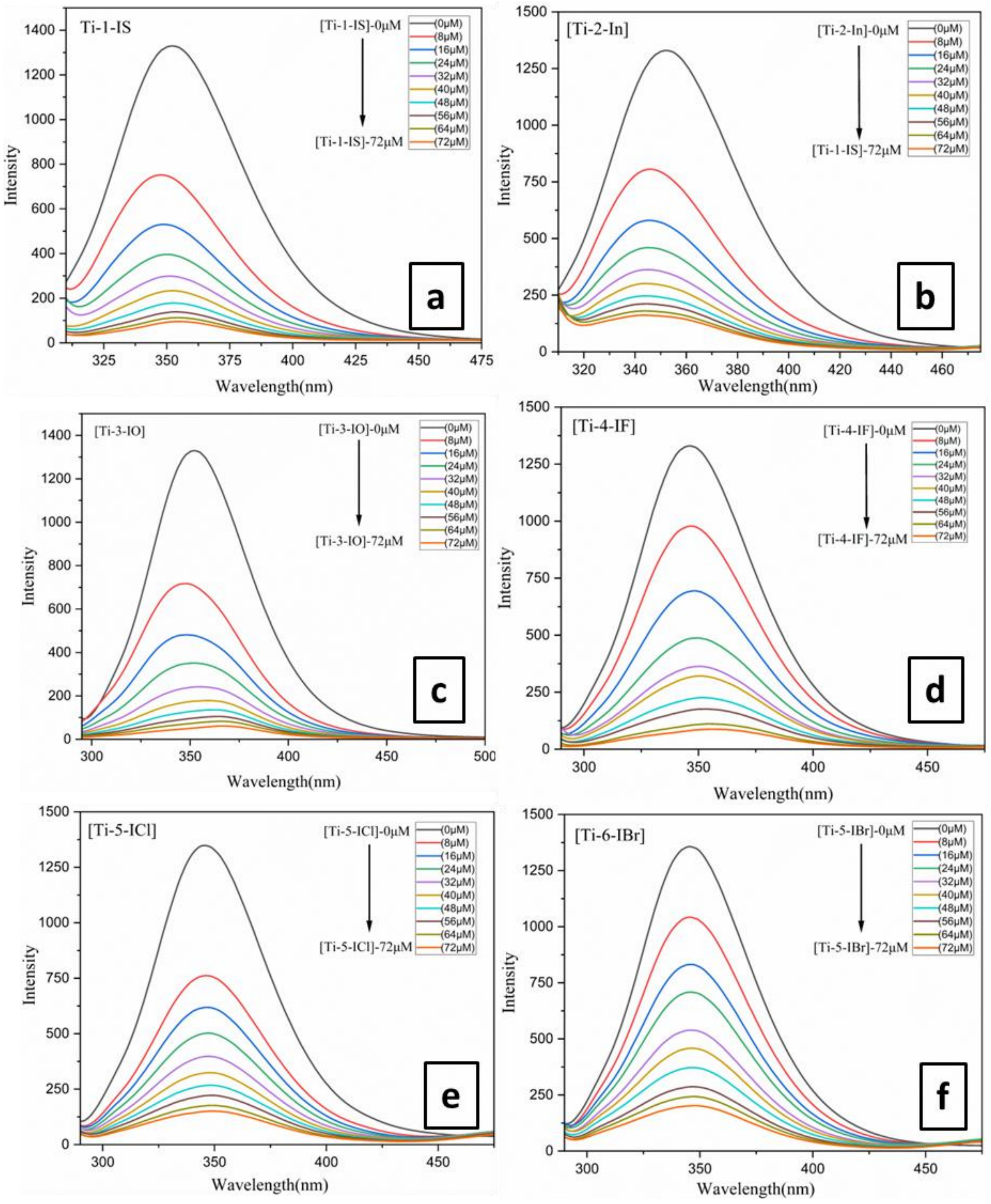

Fig.S68. Fluorescence quenching spectra of BSA with increasing concentration of Ti(IV) complexes

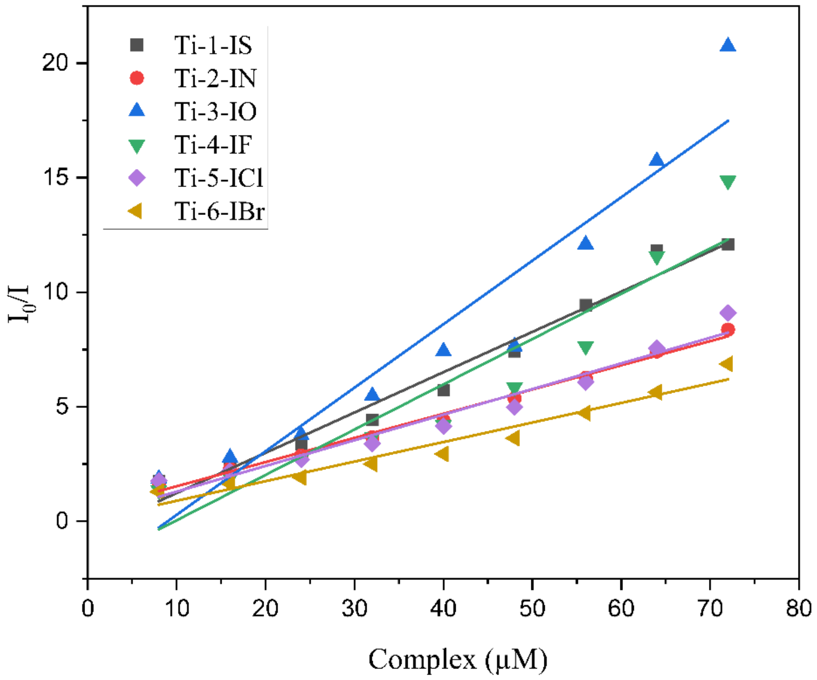

Fig.S69. Stern-Volmer plots of I_0/I vs. complex

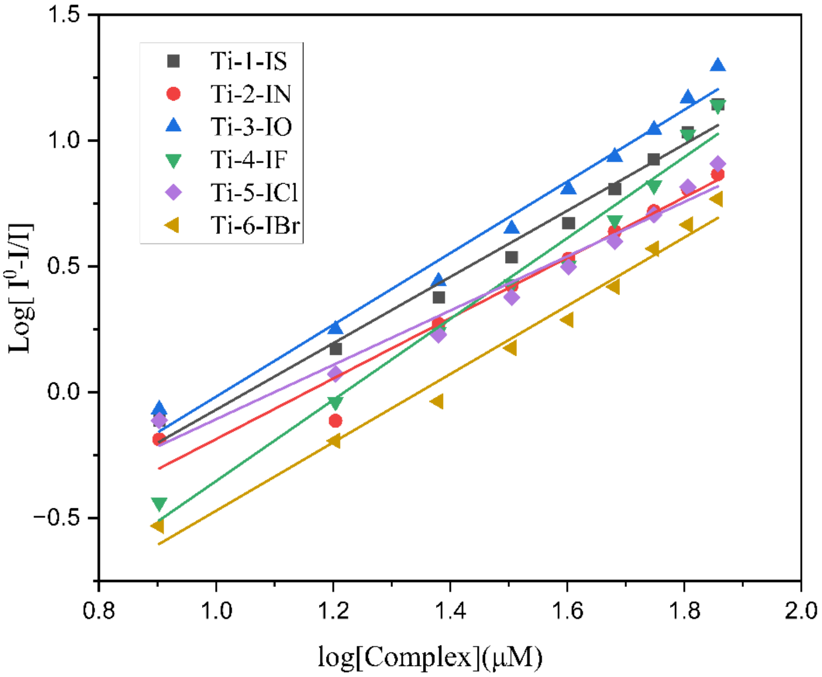

Fig.S70. Scatchard plot of log([I_0-I]/I) vs. log [complex]

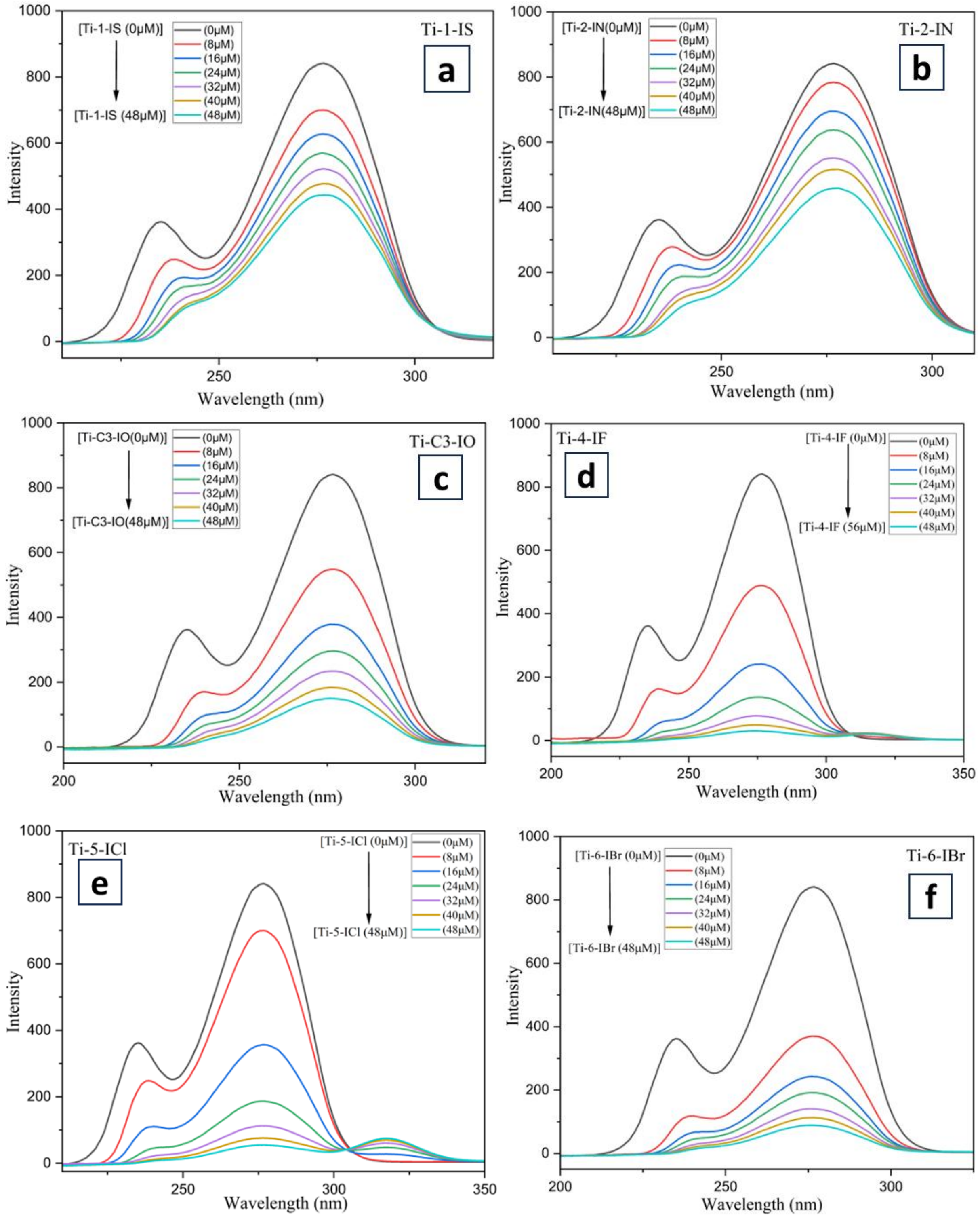

Fig.S71. Synchronous spectra of BSA with increasing concentration of Ti(IV) complexes at Δλ=15 nm

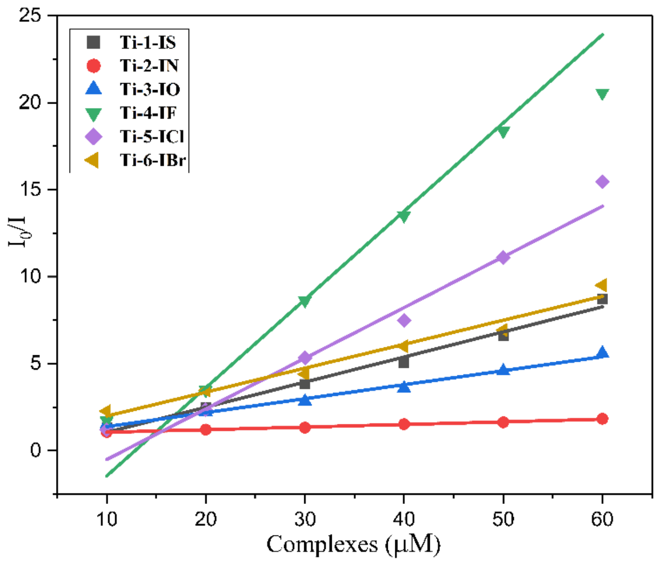

Fig.S72. Stern-Volmer plots of I_0/I vs. complex of Synchronous spectra of BSA with increasing concentration of Ti(IV) complexes at Δλ=15 nm

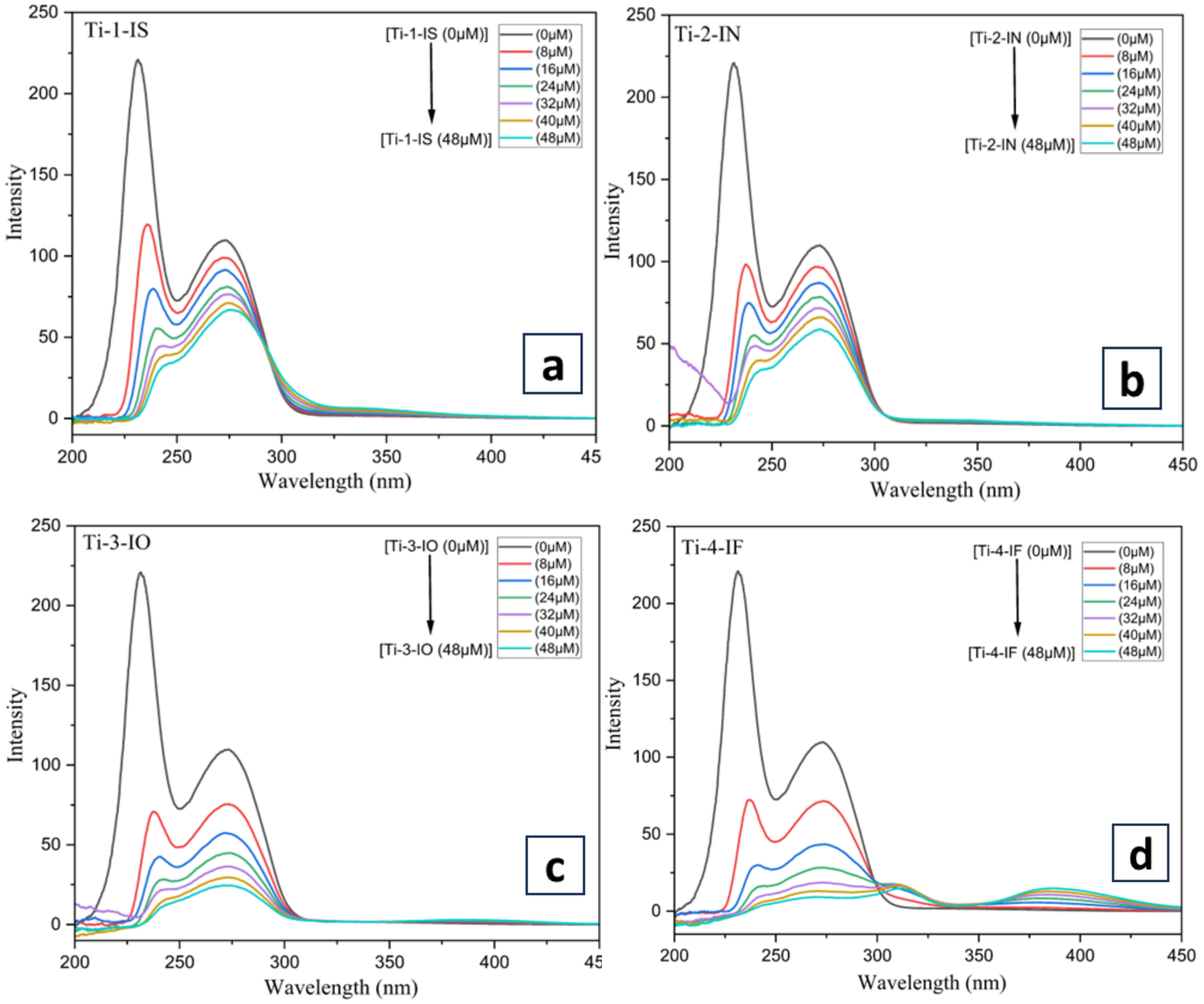

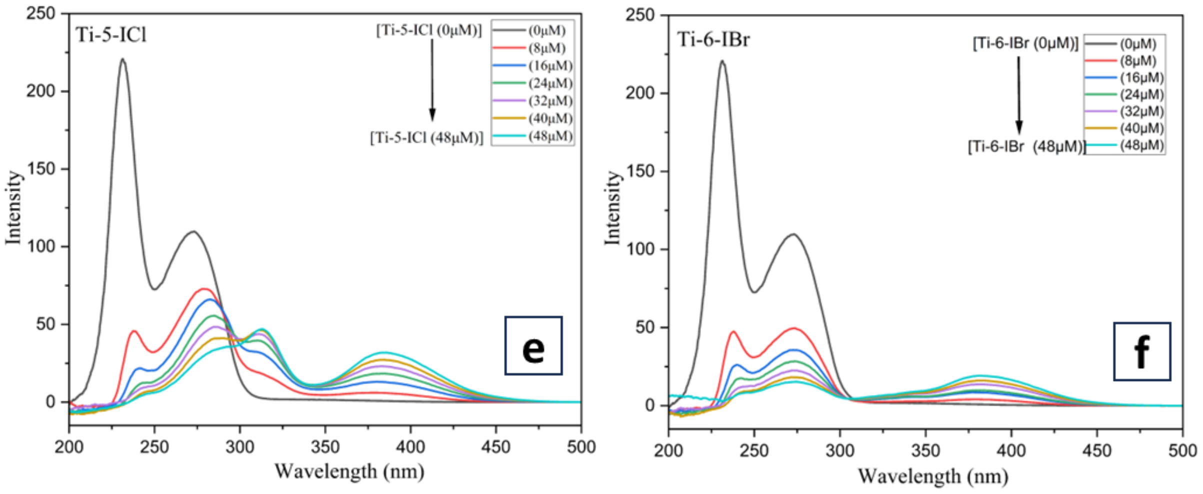

Fig.S73. Synchronous spectra of BSA with increasing concentration of Ti(IV) complexes at Δλ=60 nm

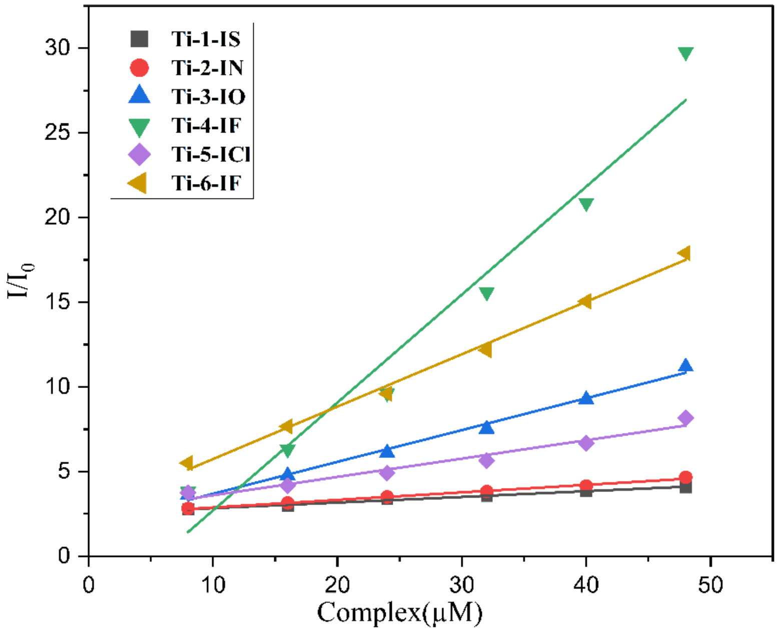

Fig.S74. Stern-Volmer plots of I_0/I vs. complex of Synchronous spectra of BSA with increasing concentration of Ti(IV) complexes at Δλ=60 nm

Table S2; Stern-Volmer plots of I_0/I vs. complex of Synchronous spectra of BSA with increasing concentration of Ru(II) complexes at Δλ=15 nm and 60nm

Complexes	Ka<br>Δλ=15nm	Ka<br>Δλ=60 nm
Ti-1-IS	0.1444	0.0334
Ti-2-IN	0.0150	0.0445
Ti-3-IO	0.0803	0.1882
Ti-4-IF	0.5071	0.6377
Ti-5-ICl	0.2911	0.1084
Ti-6-IBr	0.1376	0.3096

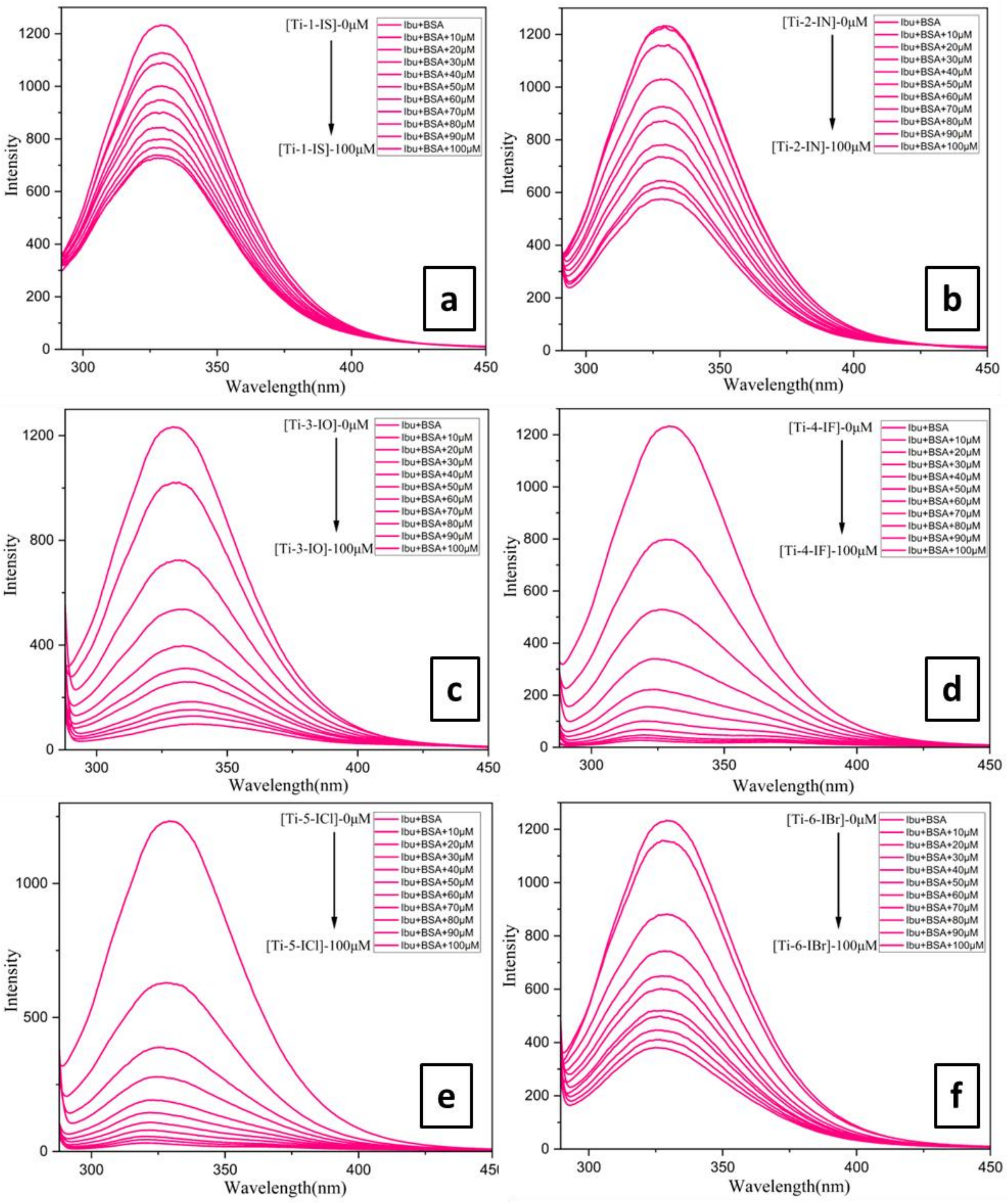

Fig.S75. Site marker fluorescence quenching studies of BSA+Ibuprofin with an increase in the concentration of Ti(IV) complexes

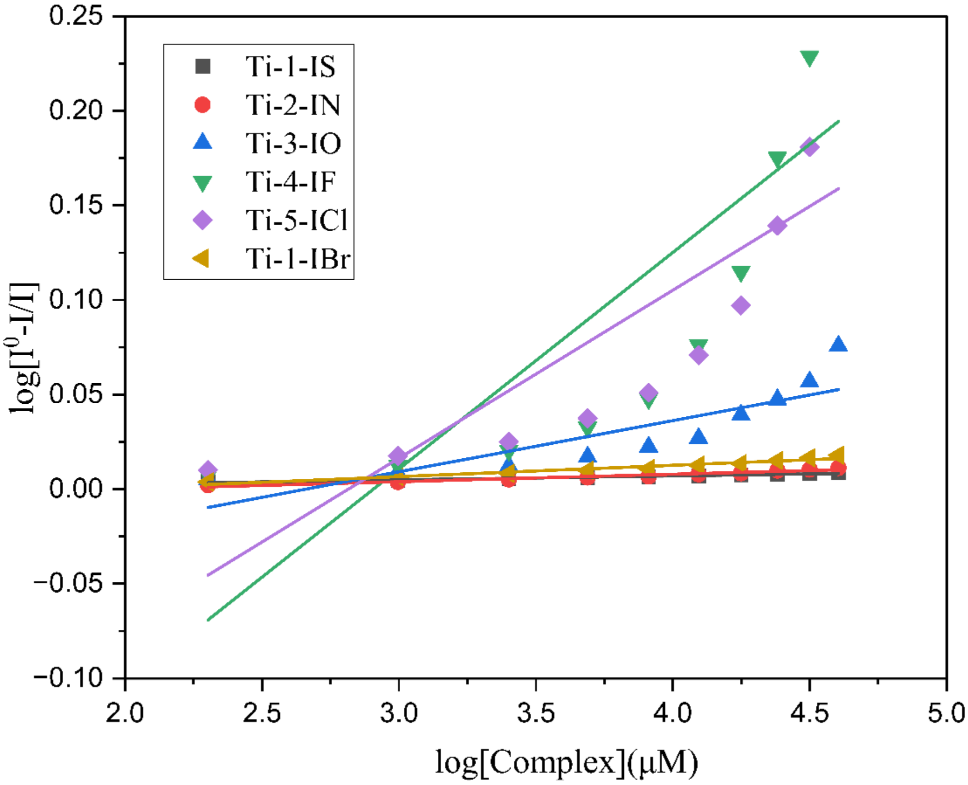

Fig.S76. Scatchard plot of log([I_0-I]/I) vs log [complex]of site marker fluorescence quenching studies of BSA+Ibuprofin

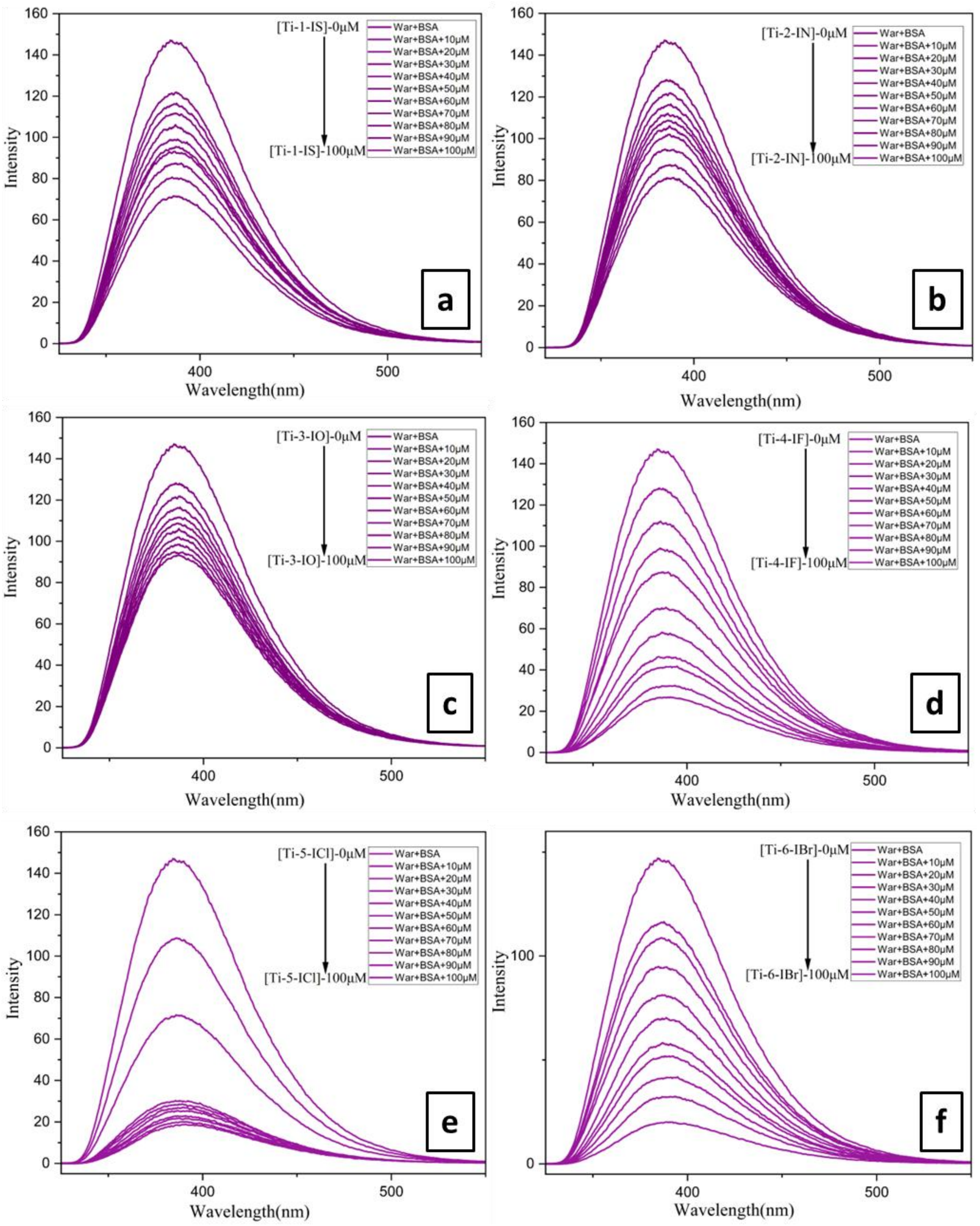

Fig.S77. Site marker fluorescence quenching studies of BSA+Warfarin with an increase in the concentration of Ti(IV) complexes

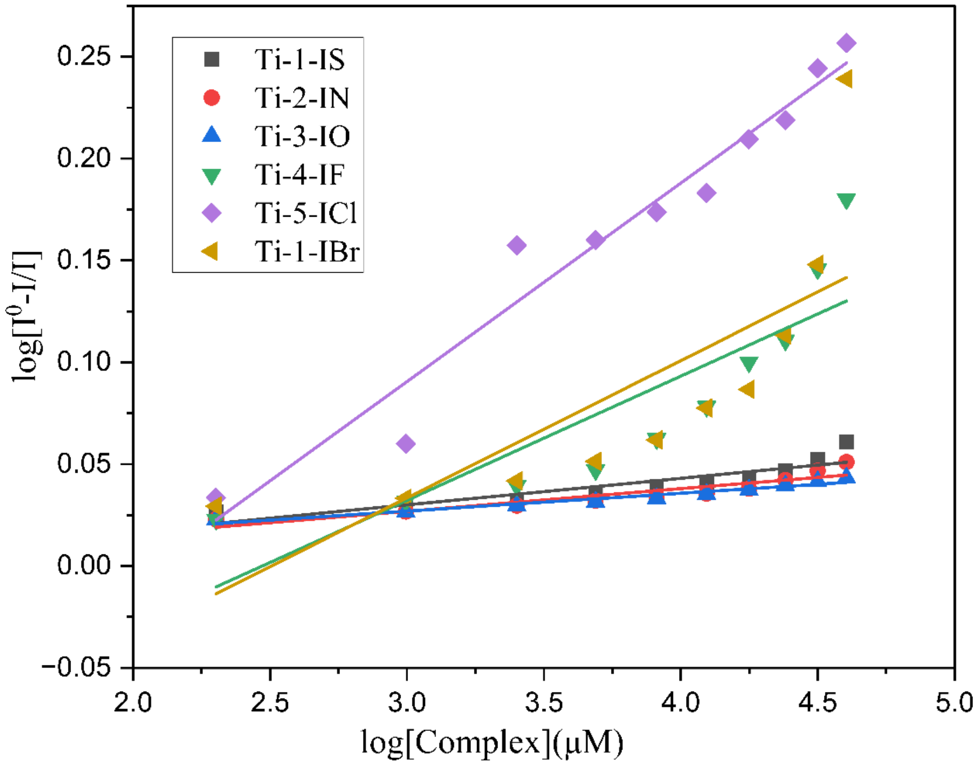

Fig.S78. Scatchard plot of log([I_0-I]/I) vs log [complex]of site marker fluorescence quenching studies of BSA+Warfarin

Table S3; The comparison of binding constants of the complexes Ti(IV) with BSA before and after the addition of site probe at 298 K. K_b binding constant [a]

COMPLEXES	BSA<br>Kb [a] (× 10^4 M^{-1})	BSA+Ibuprofen<br>Kb [a] (× 10^4 M^{-1})	BSA + Warfarin<br>Kb [a] (× 10^4 M^{-1})
**Ti-1-IS**	**0.040**	0.034	0.048
**Ti-2-IN**	**0.047**	0.015	0.069
**Ti-3-IO**	**0.036**	0.050	0.058
**Ti-4-IF**	**0.011**	0.037	0.040
**Ti-5-ICl**	**0.065**	0.035	0.016
**Ti-6-IBr**	**0.014**	0.054	0.039

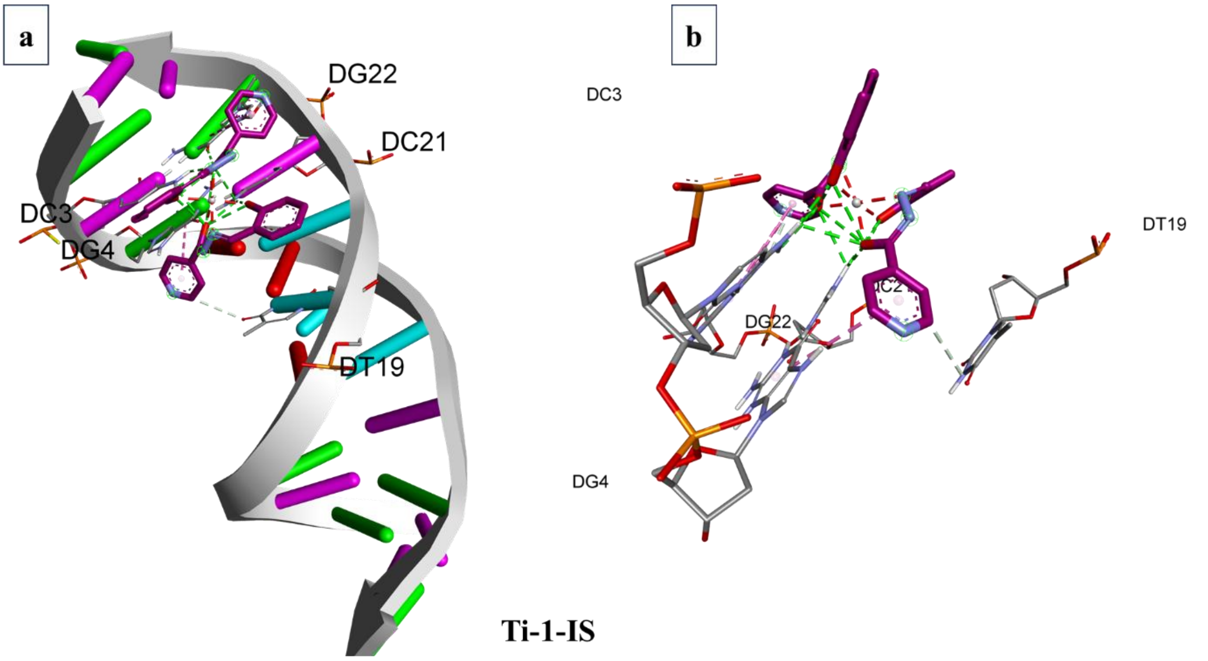

Fig.S79. Molecular docking of DNA with Ti-1-IS; purple color indicates carbon atoms, blue color indicates nitrogen, grey color indicates titanium(IV) ion, red color indicates oxygen atoms

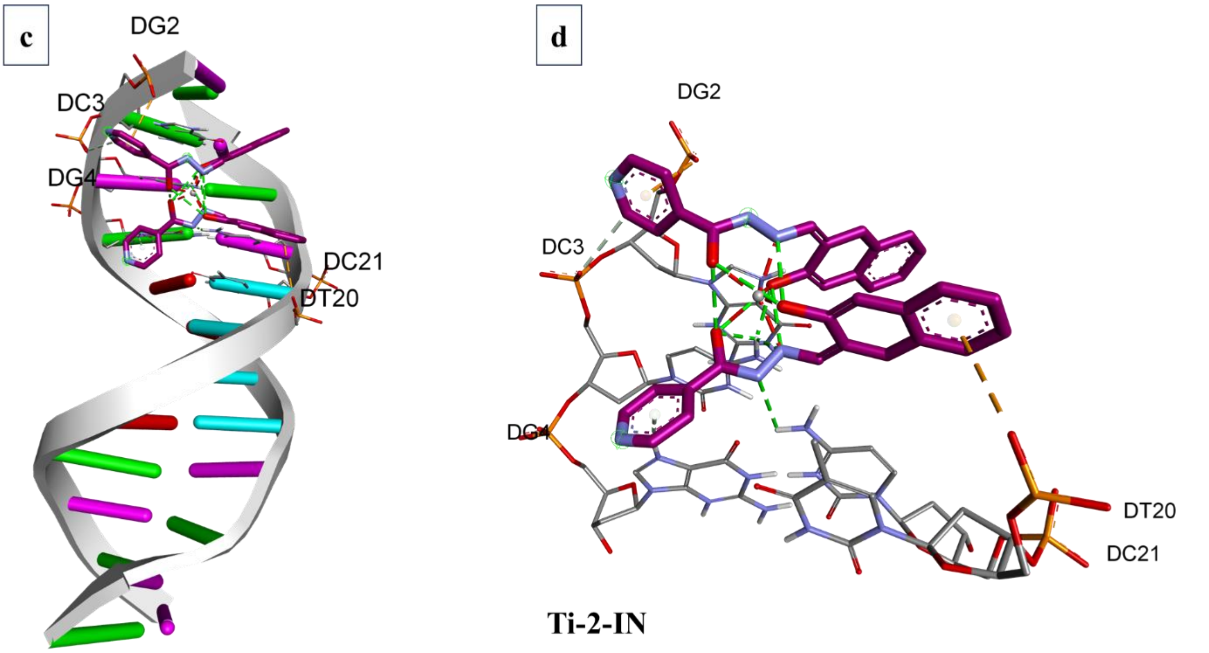

Fig.S80. Molecular docking of DNA with Ti-2-IN ; purple color indicates carbon atoms, blue color indicates nitrogen, grey color indicates titanium(IV) ion, red color indicates oxygen atoms

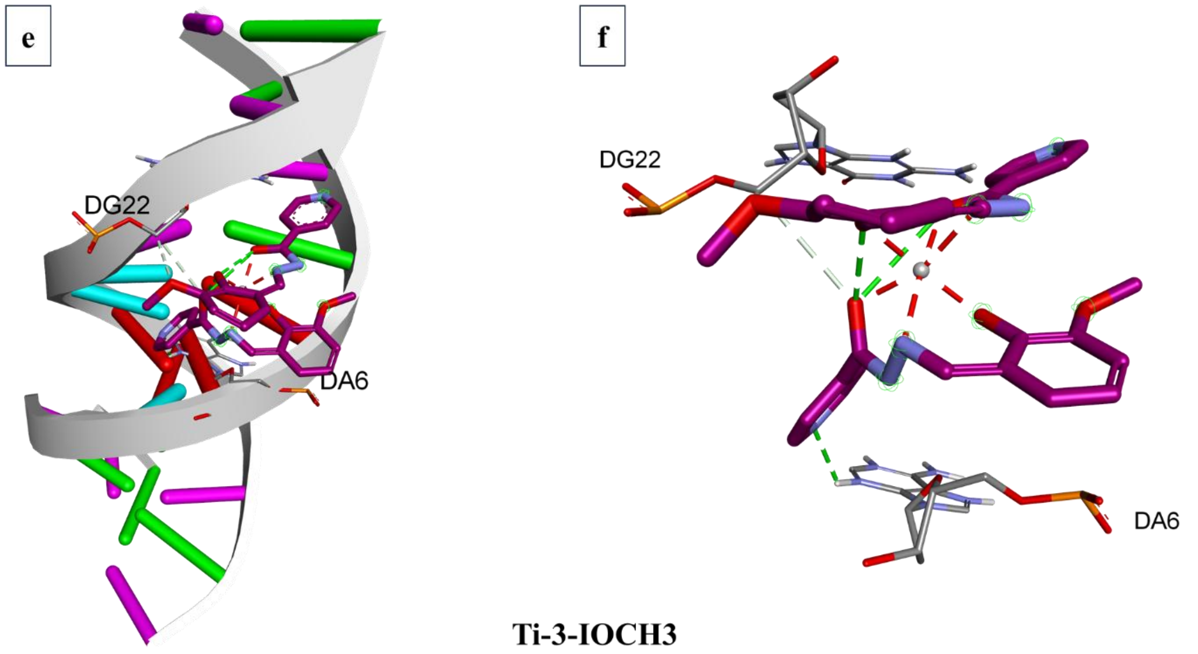

Fig.S81. Molecular docking of DNA with Ti-3-IO ; purple color indicates carbon atoms, blue color indicates nitrogen, grey color indicates titanium(IV) ion, red color indicates oxygen atoms

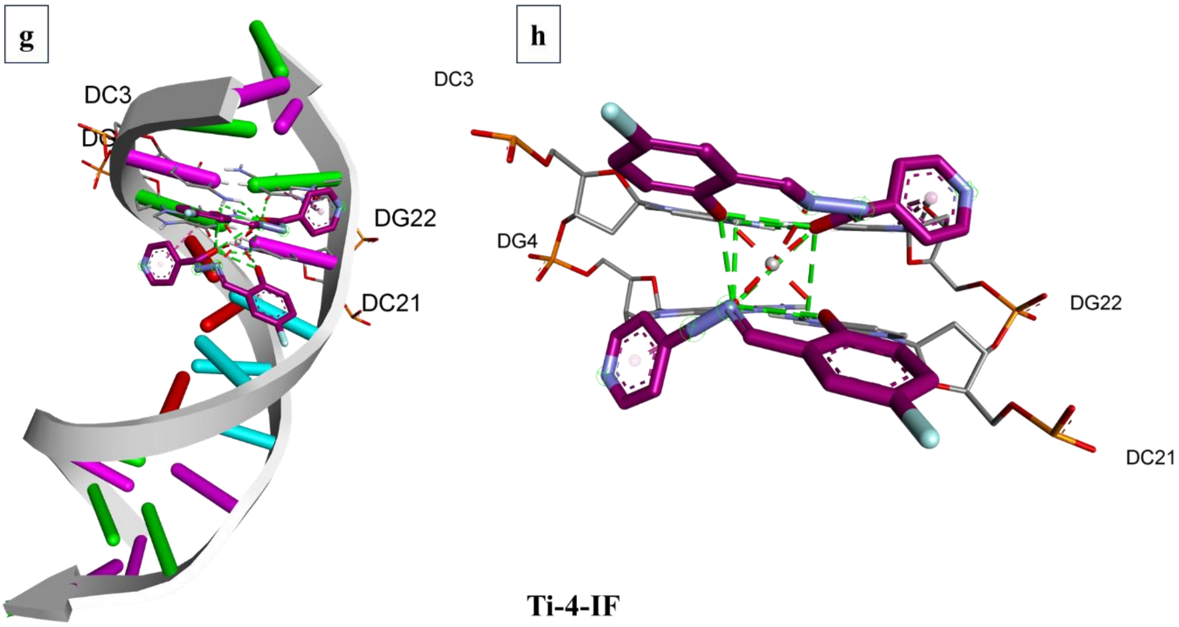

Fig.S82. Molecular docking of DNA with Ti-4-IF ; purple color indicates carbon atoms, blue color indicates nitrogen, grey color indicates titanium(IV) ion, red color indicates oxygen atoms

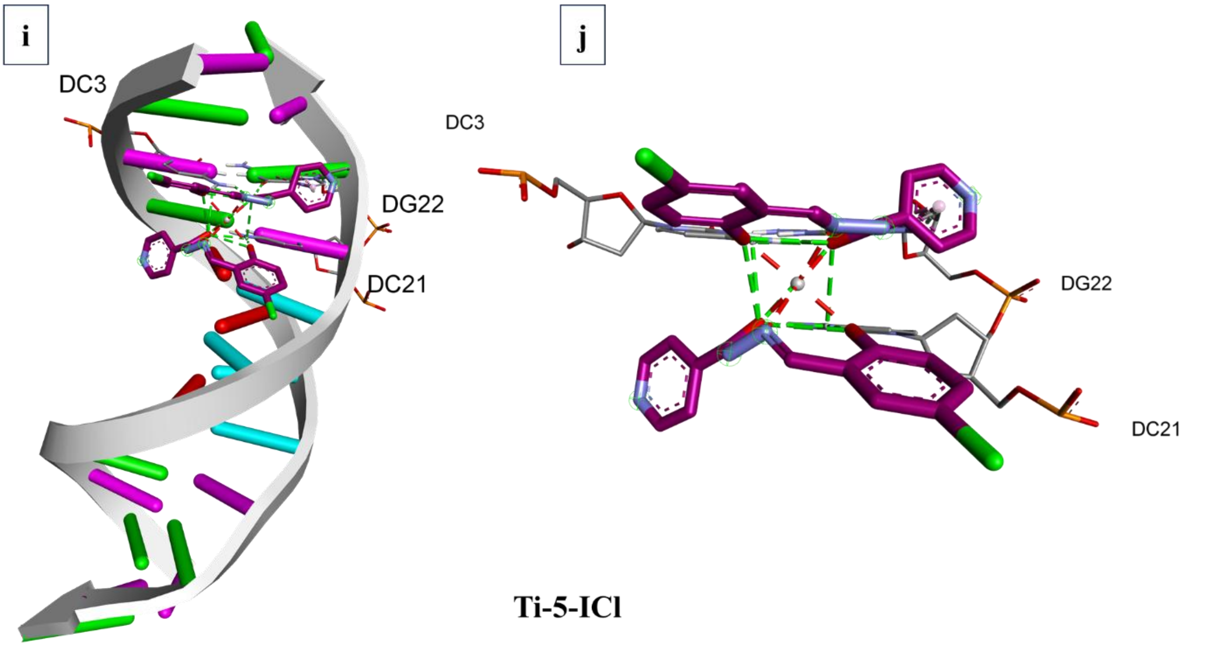

Fig.S83. Molecular docking of DNA with Ti-5-ICl ; purple color indicates carbon atoms, blue color indicates nitrogen, grey color indicates titanium(IV) ion, red color indicates oxygen atoms

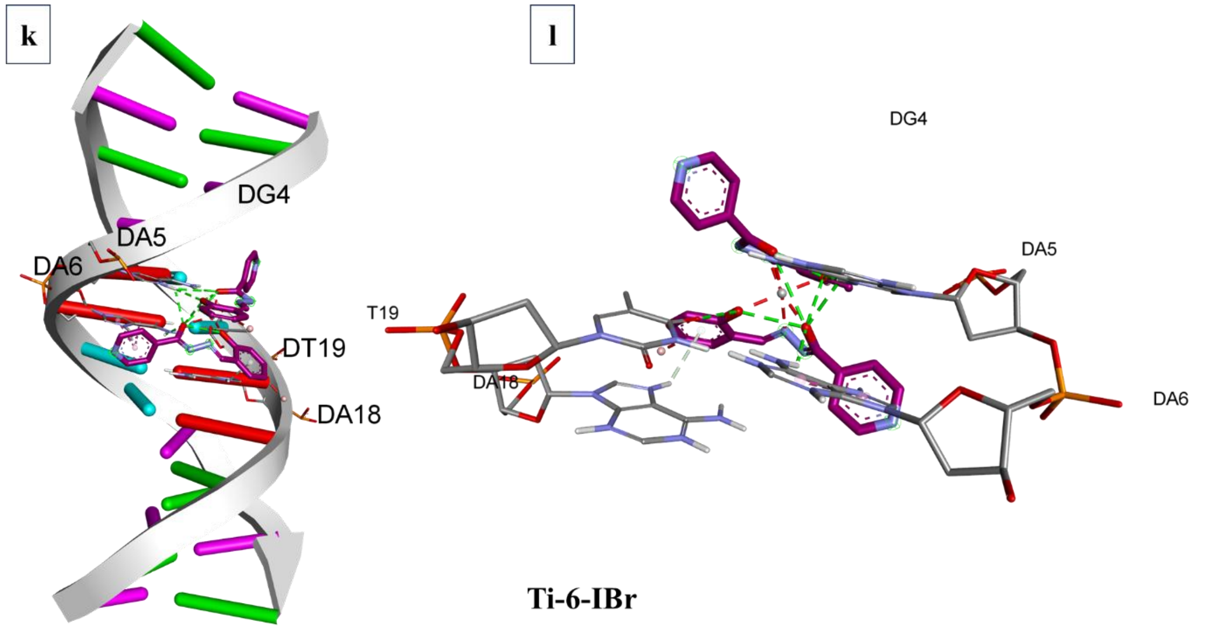

Fig.S84.Molecular docking of DNA with Ti-6-IBr ; purple color indicates carbon atoms, blue color indicates nitrogen, grey color indicates titanium(IV) ion, red color indicates oxygen atoms

Table S4; Docking scores and binding sites of DNA with Ti(IV) complexes

Complexes	Nucleotides	Docking scores in kcal/mol
Ti-1-IS	DC3, DG4, DT19, DC21, DG22	-9.3
Ti-2-IN	DG2, DG4, DC3, DC21, DT20	-10.3
Ti-3-IO	DA6, DG22	-8.4
Ti-4-IF	DC3, DG4, DC21, DG22	-9.7
Ti-5-ICl	DC21, DG22, DC3	-9.7
Ti-6-IBr	DG4,DA5,DA6,DA16, T19	-9.5

Table S5; Docking scores and binding sites of BSA with Ti(IV) complexes

Complexes	Amino acid residues	Docking scores in kcal/mol
Ti-1-IS	GLU182, ARG185, PRO117, LYS116, LEU115, LYS114, PRO516	-10.8
Ti-2-IN	SER109, LYS114, ARG427, GLU424, THR421, PRO420	-11.5
Ti-3-IO	PRO420, VAL423, ILE522, GLU424, SER109, PRO110, ARG144	-9.2
Ti-4-IF	GLU125, THR121, LEU122, LYS136, GLU140, LEU115, LYS116, ASP118	-9.8
Ti-5-ICl	ASP118, THR121, LEU122, GLU125, LYS132, LYS136, GLU140, PRO113, LEU115, LYS116, LEU115, LYS116	-9.9
Ti-6-IBr	THR121, GLU125, LYS132, LYS136, GLU140, LYS116, ASP118, LEU122	-9.7

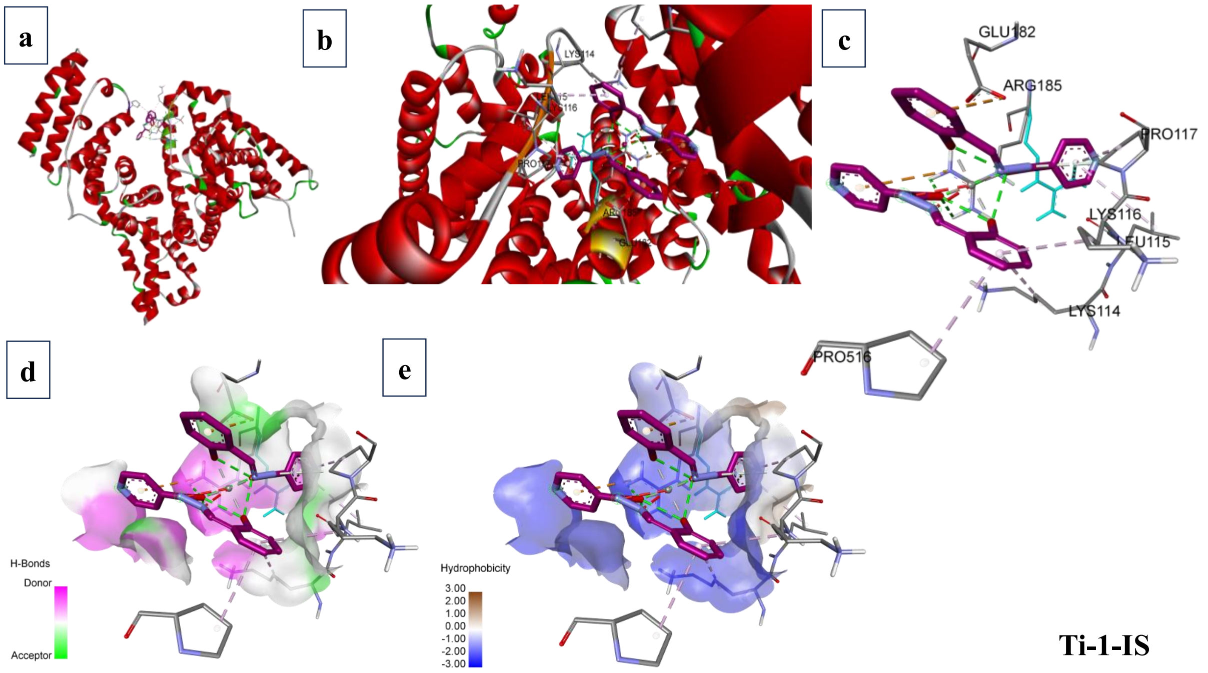

Fig.S85. Molecular docking of BSA with Ti-1-IS purple color indicates carbon atoms, blue color indicates nitrogen, grey color indicates titanium(IV) ion, red color indicates oxygen atoms

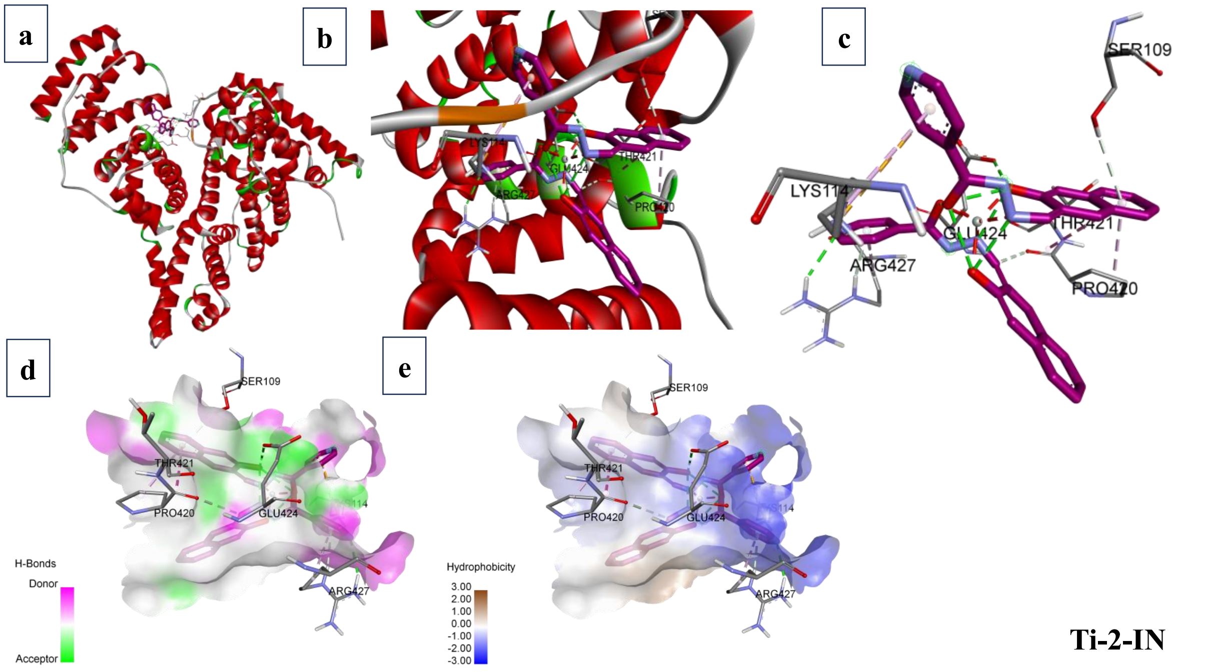

Fig.S86. Molecular docking of BSA with Ti-2-IN purple color indicates carbon atoms, blue color indicates nitrogen, grey color indicates titanium(IV) ion, red color indicates oxygen atoms

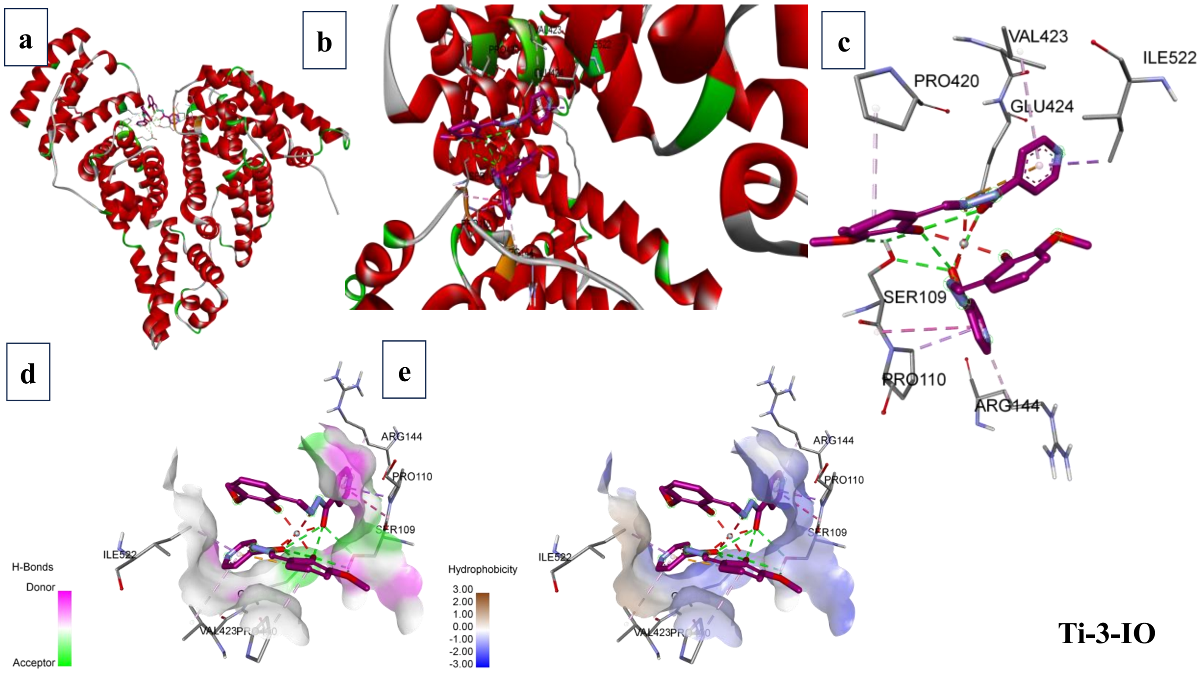

Fig.S87. Molecular docking of BSA with Ti-3-IO purple color indicates carbon atoms, blue color indicates nitrogen, grey color indicates titanium(IV) ion, red color indicates oxygen atoms

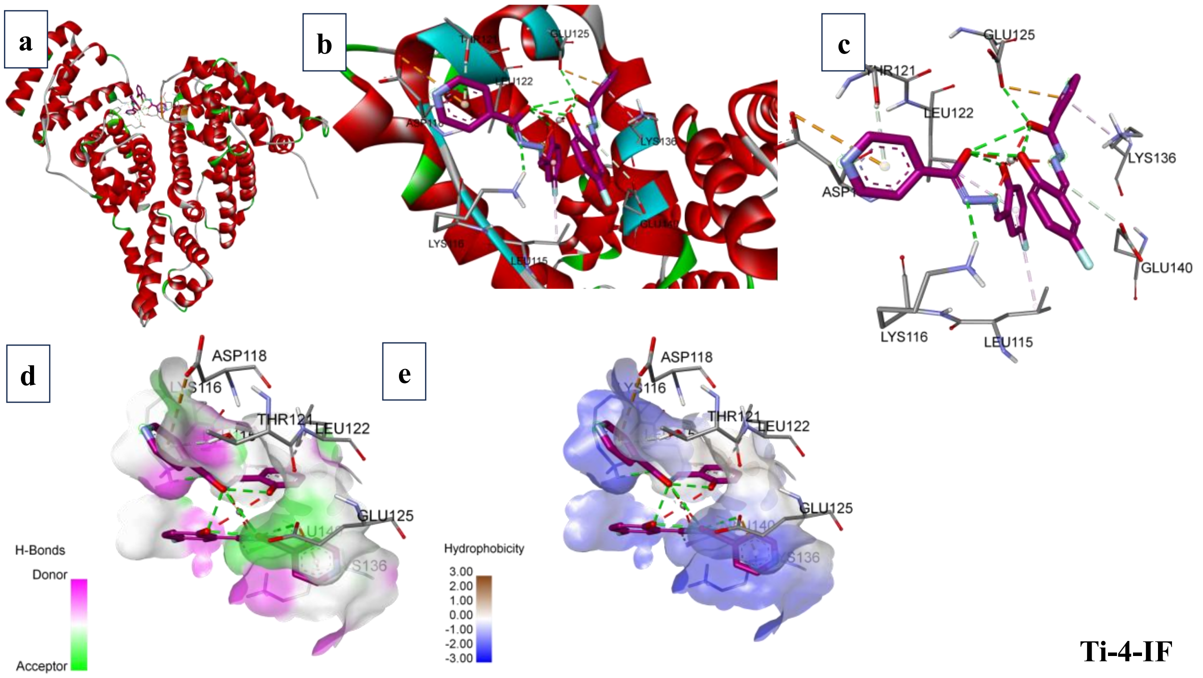

Fig.S88. Molecular docking of BSA with Ti-4-IF purple color indicates carbon atoms, blue color indicates nitrogen, grey color indicates titanium(IV) ion, red color indicates oxygen atoms

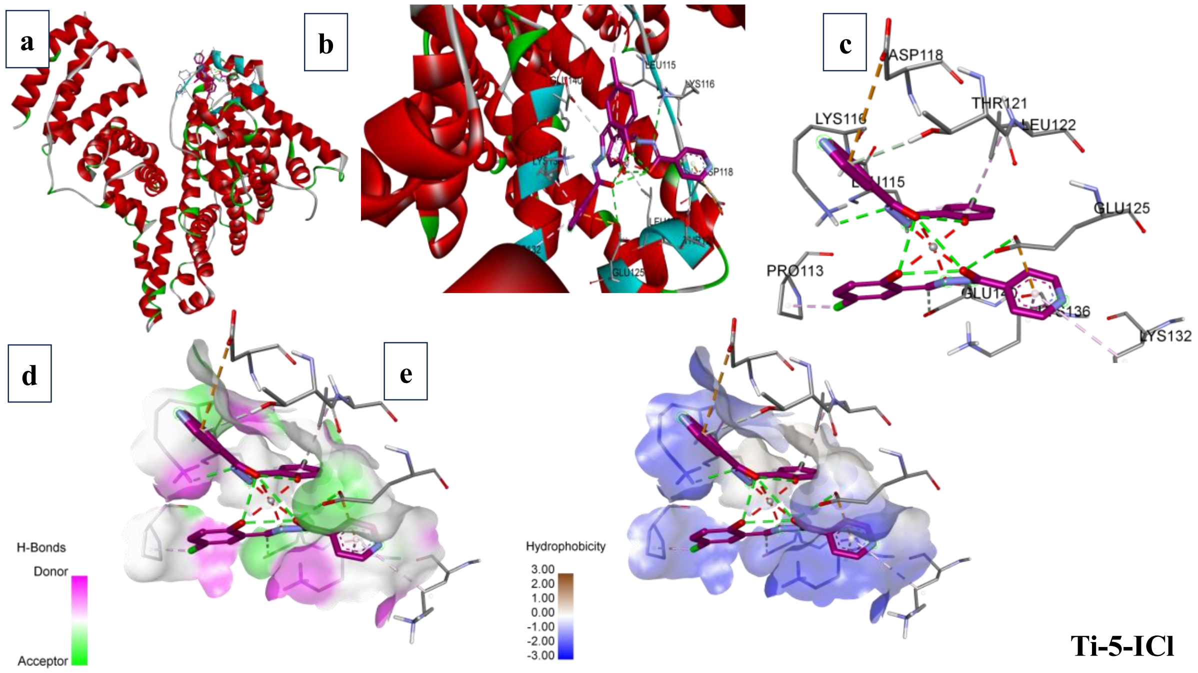

Fig.S89. Molecular docking of BSA with Ti-5-ICl purple color indicates carbon atoms, blue color indicates nitrogen, grey color indicates titanium(IV) ion, red color indicates oxygen atoms

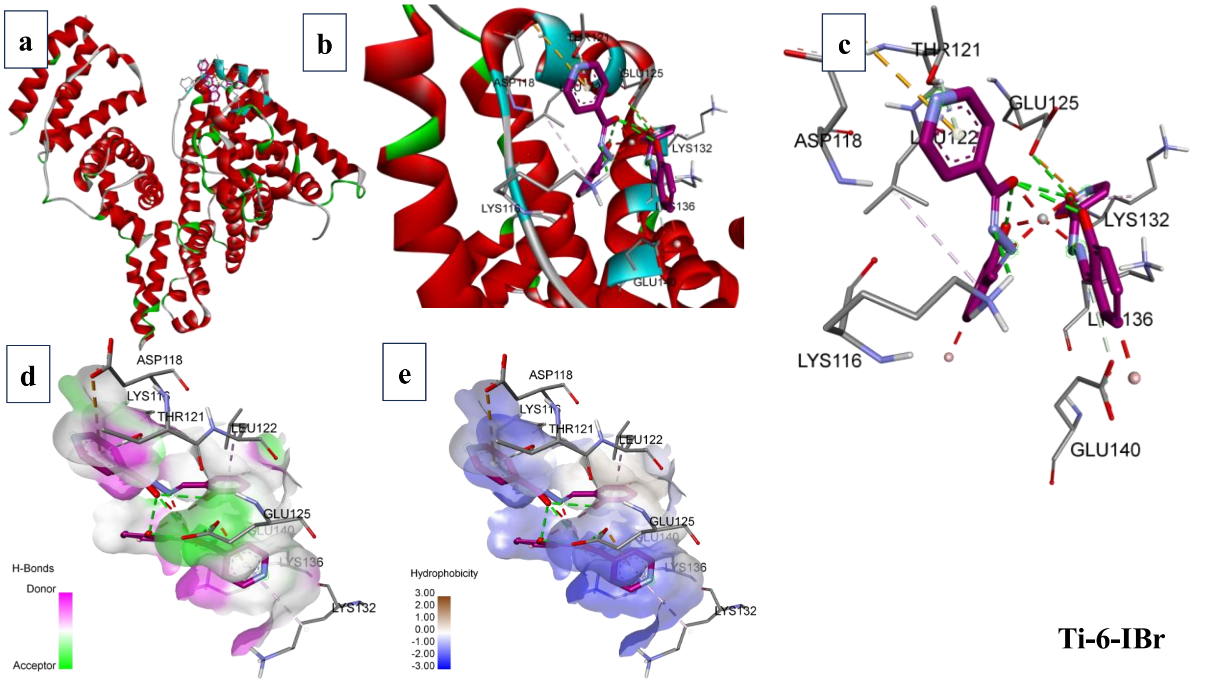

Fig.S90. Molecular docking of BSA with Ti-6-IBr purple color indicates carbon atoms, blue color indicates nitrogen, grey color indicates titanium(IV) ion, red color indicates oxygen atoms

Table S6; Bond length (Å) of Ti(IV) complexes

S. NO	Code	O-Ti	N-Ti	O-Ti
1	**Ti-1-IS**	1.997	2.886	2.032
2	**Ti-2-IN**	1.997	2.887	2.031
3	**Ti-3-IO**	2.031	2.882	2.032
4	**Ti-4-IF**	1.998	2.885	2.030
5	**Ti-5-ICl**	2.001	2.886	2.031
6	**Ti-6-IBr**	2.000	2.885	2.029

Table S7; Comparison of experimental and theoretical excitation spectral details

Code	Experimental	Theoretical Prediction			
	Abs (nm)	Abs. max (nm)	Oscillator strength (f)	Transition	Orbital Contribution
**Ti-1-IS**	339	389	0.325	$S_0 \rightarrow S9$	H→L+4 82%, H-3→L 3%,
**Ti-2-IN**	341	378	0.233	$S_0 \rightarrow S_{10}$	H→L+4 46%, H-3→L 19%,
**Ti-3-IO**	340	412	0.298	$S_0 \rightarrow S_{12}$	H→L+3 68%, H-3→L 12%,
**Ti-4-IF**	343	406	0.282	$S_0 \rightarrow S_{10}$	H→L+5 48%, H-4→L 26%,
**Ti-5-ICl**	345	443	0.316	$S_0 \rightarrow S_{11}$	H→L+3 55%, H→L+4 21%,
**Ti-6-IBr**	343	625	0.238	$S_0 \rightarrow S_{10}$	H→L+5 78%, H-4→L 6%,

*H – HOMO, L - LUMO

H→L+4 83%, H-4→L+2 4%

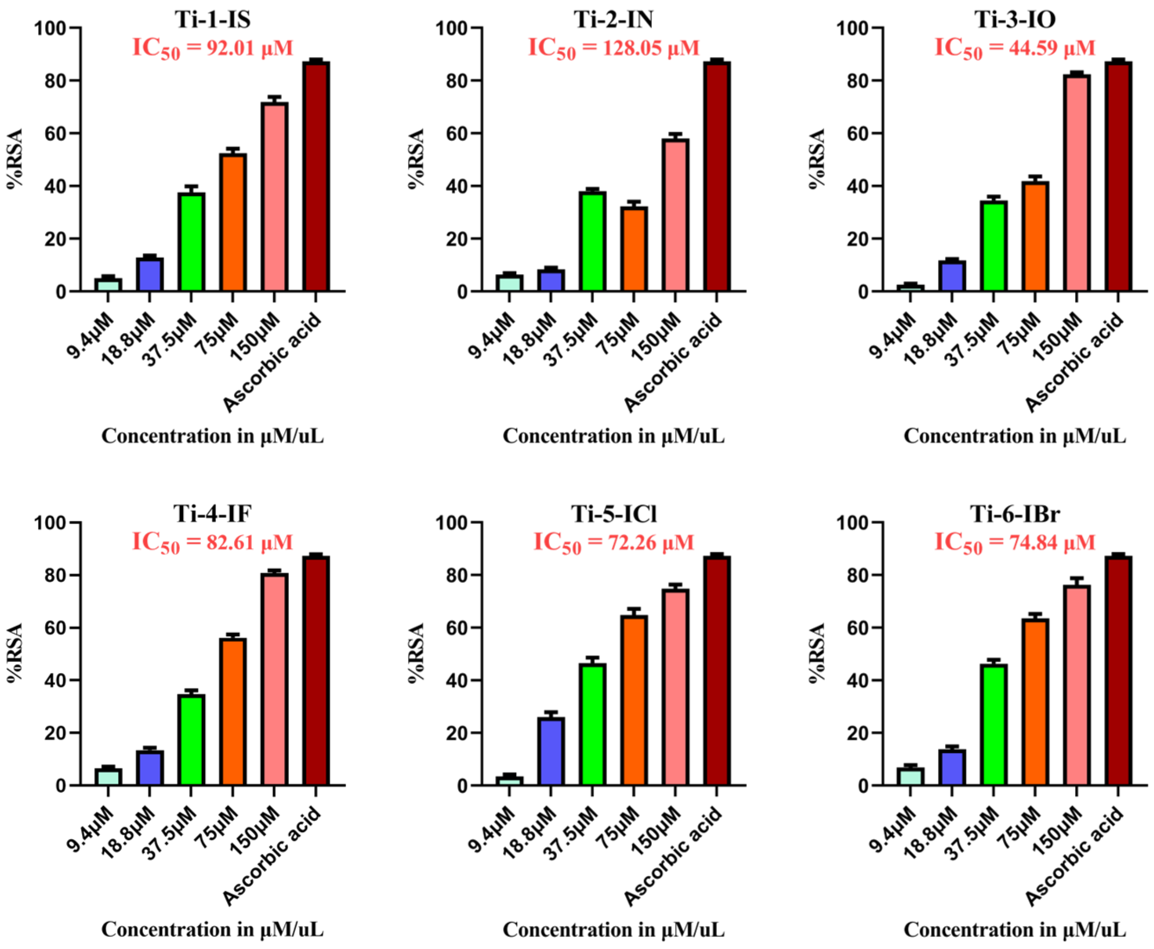

Fig.S91. DPPH assay of Ti(IV) complexes

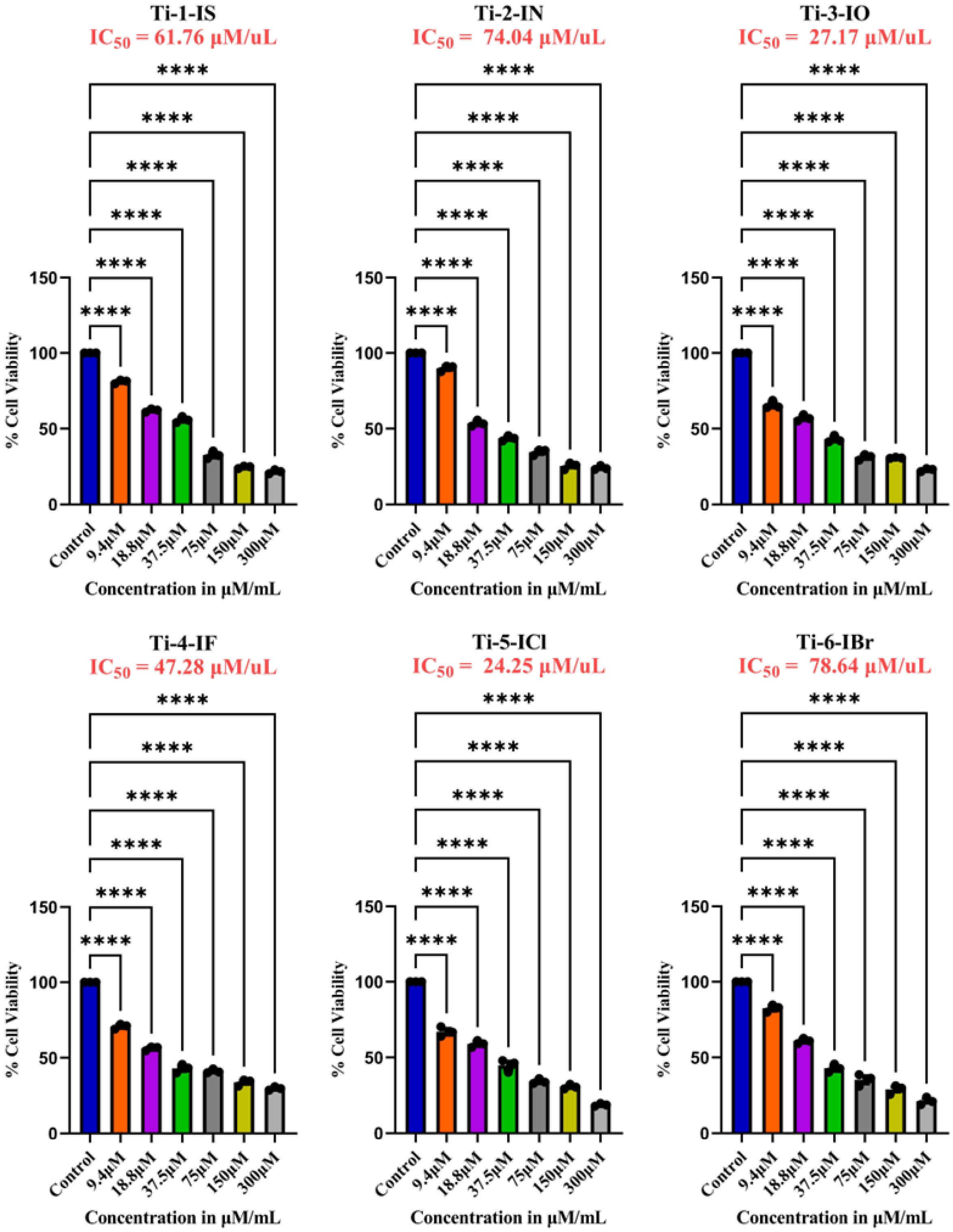

Fig.S92. MTT assay of Ti(IV) complexes on HeLa cell line

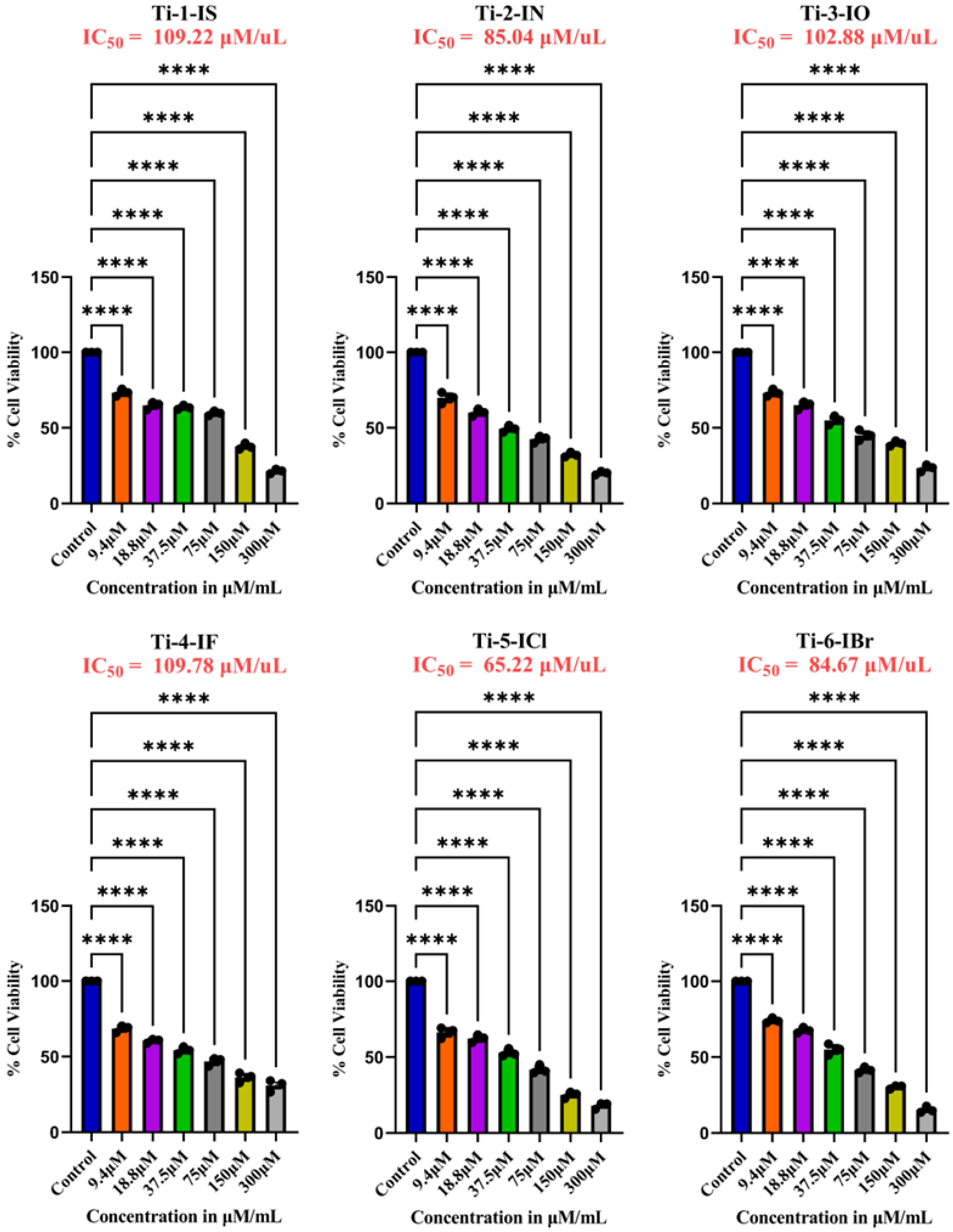

Fig.S93. MTT assay of Ti(IV) complexes on MCF7 cell line

References:

1. Subramaniyan, Mahabarathi, et al. "New bioactive titanium (IV) derivatives with their DFT, molecular docking, DNA/BSA interaction, antioxidant and in-vitro investigations." *Inorganica Chimica Acta* 571 (2024): 122191.
2. Thanigachalam, Sathish, et al. "Bioactive O^ N^ O^ Schiff base appended homoleptic titanium (iv) complexes: DFT, BSA/CT-DNA interactions, molecular docking and antitumor activity against HeLa and A549 cell lines." *RSC advances* 14.19 (2024): 13062-13082.
3. Dorairaj, Dorothy Priyanka, et al. "Ru (II)-p-Cymene complexes of furoylthiourea ligands for anticancer applications against breast cancer cells." *Inorganic Chemistry* 62.30 (2023): 11761-11774.
4. Haribabu, Jebiti, et al. "Michael addition-driven synthesis of cytotoxic palladium (ii) complexes from chromone thiosemicarbazones: investigation of anticancer activity through in vitro and in vivo studies." *New Journal of Chemistry* 47.33 (2023): 15748-15759.
5. Arunachalam, Abirami, et al. "Synthesis and Structure of Naphthoyl Thiourea-Based Binuclear Ruthenium (II) Arene Complexes: Studies on Anticancer Activity and Apoptotic Mechanism." *ChemBioChem*: e202500057.
6. Monika, Sankar, et al. "Binuclear Ruthenium (II) Complexes Featuring Arylthiourea Ligands: Synthesis, Crystal Structure and Anticancer Assessment." *Applied Organometallic Chemistry* 39.5 (2025): e70146.
7. Kar, Binoy, et al. "2-Aryl-1 H-imidazo [4, 5-f][1, 10] phenanthroline-Based Binuclear Ru (II)/Ir (III)/Re (I) Complexes as Mitochondria Targeting Cancer Stem Cell Therapeutic Agents#." *Journal of Medicinal Chemistry* 67.13 (2024): 10928-10945.
8. Das, Utpal, et al. "Exploring the phototoxicity of GSH-resistant 2-(5, 6-dichloro-1 H-benzo [d] imidazol-2-yl) quinoline-based Ir (III)-PTA complexes in MDA-MB-231 cancer cells." *Dalton Transactions* 53.14 (2024): 6459-6471.
